# Stress induced dynamic adjustment of conserved miR164:NAC module

**DOI:** 10.1002/pei3.10027

**Published:** 2020-08-10

**Authors:** Yuniet Hernandez, Kavita Goswami, Neeti Sanan‐Mishra

**Affiliations:** ^1^ Plant RNAi Biology Group International Centre for Genetic Engineering and Biotechnology New Delhi India

**Keywords:** drought stress, microRNA, NAC transcription factor, salt stress, soybean

## Abstract

**Aims, including the rationale:**

Salinity and drought are the two major stresses limiting the productivity of economically important crops such as *Glycine max* (soybean). The incidence of these stresses during the pod development stages affects the quality and quantity of seeds, which compromise the yield of soybean. The miR164:*NAC* module has been shown to play a critical role in regulating the response to salt and drought stress in several plant species. However, biological role of miR164:*NAC* module in salt stress in soybean is not fully understood.

**Methods:**

In this study, we identified 215 salt responsive miRNAs, using miScript miRNA array with a sensitive and a tolerant soybean genotype, William82 and INCASoy36, respectively. The targets of these salt regulated miRNAs were searched in the degradome datasets.

**Key results:**

It was found that four salt stress deregulated miRNAs targeted the *NAC* transcription factor and among these miR164k and miR408d showed antagonistic expression in the two soybean genotypes. The expression of miR164k was higher in salt tolerant INCASoy36 as compared to salt sensitive William82, under unstressed conditions. However under salt stress, miR164k was downregulated in INCASoy36 (−2.65 fold), whereas it was upregulated in William82 (4.68 fold). A transient co‐expression assay validated that gma‐miR164k directs the cleavage of GmNAC1 transcript. Bioinformatics analysis revealed that the regulation of NAC transcription factor family by members of miR164 family is conserved across many species. The dynamic expression profiles of miR164 and NAC‐TFs were captured in different tissues of rice, tobacco, and two soybean genotypes under drought and salt stress conditions.

**Main conclusion:**

Collectively, our results suggest that genetically determined dynamic modulation of the conserved miR164:*NAC‐TF* module may play an important role in determining the adaptive response of plants to stress.

## 1 INTRODUCTION

Drought and salinity are related stresses that may occur simultaneously in natural conditions. Drought induces non‐ionic osmotic stress while salinity exerts ionic osmotic stress triggering similar adaptive responses in plants. Over millions of years, plants have evolved diverse mechanisms that allow them to respond and cope with adverse environmental conditions. However, some of these adaptive mechanisms might have been lost in most modern cultivars owing to domestication and intensive selection of traits related to yield (Hyten et al., 2006), making crops vulnerable to harsh climate changes (Berger et al., 2012; Butcher et al., 2018). Soybean, the most widely cultivated legume in the world, has lost substantial genetic diversity through its domestication (Li et al., 2014). Given the economic importance of the crop, the main factors that negatively affect its productivity need to be identified. It is accepted as a glycophytic species that shows drastic yield reduction upon water scarcity and increased soil salt (Butcher et al., 2018; Mondani et al., 2019). Both stresses induce wide range of morphological, physiological, biochemical, and molecular alterations in soybean plants, including reduced nodulation (Miransari and Smith, 2009), decreased photosynthesis, quantitative and qualitative changes in protein synthesis and induction of stress‐responsive genes (Xu et al., 2018).

Of late, there is increase in interest to improve the tolerance of soybean genotypes to salinity and drought, by employing methods of traditional improvement and selection or genetic engineering. However, progress in this field will be ensured when the genetic basis of stress tolerance and the molecular mechanisms through which plants perceive stress and translate the stress signal are better understood. In this context, extensive efforts have been made to elucidate the gene regulatory networks underlying adaptive response to stress in soybean (Tian et al., 2017; Xu et al., 2016).

The advent of high throughput RNA‐seq technology has enabled the understanding of the complex and overlapping gene regulatory networks in response to abiotic stress (Khan et al., 2017; Zheng et al., 2016). This has revealed a comprehensive picture of the stress responsive microRNAs (miRNAs) and transcription factors (TFs) in different species (Hu et al., 2018; Khan et al., 2017; Pandey et al., 2014; Tian et al., 2017). Both miRNAs and TFs are key players that regulate coordinated expression of genes in response to a particular environmental stimulus or internal cue (Imran et al., 2018; Martinez and Walhout, 2009) to drive majority of the cellular processes. The miRNAs are small, non‐coding RNAs that mediate post‐transcriptional gene suppression in a sequence‐specific manner. TFs are preferential targets of the miRNAs and also regulate their transcription (Akdogan et al., 2016; Han et al., 2014; Peng et al., 2014; Zheng et al., 2016). Comprehension of how these two *trans*‐regulators interact with each other to intricately coordinate gene expression at the genome level is crucial to decipher the mechanism underlying abiotic stress acclimation.

Accumulated evidence demonstrates that several families of TFs, including bZIP (basic leucine zipper), MYB, SPL (SQUAMOSA promoter‐binding protein‐like), AP2/ERF (*apetala* 2/ethylene‐responsive element binding factor), MADS‐box and NAC are targeted by miR166, miR159, miR156, miR444, miR172 and miR164, respectively (Kim et al., 2009; Mittal et al., 2016). These regulatory nodes have the potential to improve abiotic stress tolerance in plants (Cui et al., 2015; Li et al., 2016; Lu et al., 2017; Zhang et al., 2018; Zhou et al., 2013). Among these, NAC protein family is one of the largest and ancient families of plant TFs. The members that comprise NAC family like CUC1/CUC2, NAC1, NTL9, and ORE1, have been involved in diverse biological processes including development, defense and abiotic stress response (Ge et al., 2017; Guo and Gan, 2006; Singh et al., 2013; Xie et al., 2000).

Expression of several *NAC* genes has been found to be induced by different stresses in both ABA‐dependent and ABA‐independent manner (Nuruzzaman et al., 2013; Puranik et al., 2012; Shao et al., 2015). Functional characterization of different NAC‐TFs in response to dehydration, salinity and osmotic stresses supports their role in enhancing tolerance (Huang et al., 2016; Jeong et al., 2013; Nakashima et al., 2012; Xu et al., 2013). AtNAC72, AtNAC109, and AtNAC55 were shown to impart drought tolerance by promoting the detoxification of aldehydes in the glyoxalase pathway (Fujita et al., 2004). Investigation into the mechanisms of other NAC‐TFs revealed that they act in a variety of ways by controlling stomatal closure (Hu et al., 2006), increasing cell membrane stability and enhancing expression of several antioxidant enzymes such as *POD* (peroxidase), *SOD* (superoxide dismutase) and *P5CS* (Pyrroline‐5‐carboxylate synthase), for increasing ROS (reactive oxygen species) scavenging ability (He et al., 2019; Hong et al., 2016; Wang et al., 2017; Xu et al., 2015). JUBGBRUNNEN1 (JUB1 or NAC042), a central regulator of plant growth (Shahnejat‐Bushehri et al., 2016) and stress response (Wu et al., 2012), confers tolerance to various osmotic and ionic stresses including salt and drought (Alshareef et al., 2019) by reducing H_2_O_2_ levels (Thirumalaikumar et al., 2018; Wu et al., 2012). In soybean, 31 *NAC* genes were screened and nine of them were found to be induced by drought stress (Tran et al., 2009). The expression of *GmNAC2/3/4* was significantly induced by osmotic pressure and *GmNAC3/4* expression was simultaneously induced by ABA, JA, and salt (Pinheiro et al., 2009). Although several advances have been achieved in the study of NAC‐TFs the intricate regulation of different NAC‐TFs under salt and drought stress remains largely unknown.

The role of miR164 in suppressing the transcripts of the development‐associated and stress‐responsive NAC transcription factors is well‐studied (Hernandez and Sanan‐Mishra, 2017). The role miR164:*NAC* module in ovule initiation, root formation and various other aspects of plant growth and development has been reported (Ge et al., 2017; Gonçalves et al., 2015; Guo et al., 2005; Wang et al., 2016). It affects plant development through regulation of auxin signaling (Fu et al., 2013; Guo et al., 2005). The tae‐miR164 regulating *TaNAC21/22* negatively modulates resistance of *Triticum aestivum* (wheat) to *Puccinia striiformis* (Feng et al., 2014). Likewise, miR164 participates in plant defense against *Verticillium dahliae* by post‐transcriptionally regulating the expression of a *NAC*‐TF (Hu et al., 2018). Comprehensive analysis of miR164:*NAC* module also showed its involvement in response to drought and salt stress in several plant species (Akdogan et al., 2016; Hernandez and Sanan‐Mishra, 2017; Pandey et al., 2014). For instance, in moso bamboo, ped‐miR164:*PeSNAC1* controls tolerance to salinity and drought through regulation of lateral root development (Wang et al., 2016). This indicates co‐evolution of miRNA and its targets to regulate specific functions (Cui et al., 2017).

miR164:NAC is the well‐studied genetic module in different species like *Arabidopsis thaliana* (arabidopsis), *Zea mays* (maize), *Nicotiana tabaccum* (tobacco), *Ammopiptanthus mongolicus* (ammopiptanthus) and *Phyllostachys edulis* (moso bamboo) (Fu et al., 2013; Gao et al., 2016; Guo et al., 2005; Li et al., 2012; Wang et al., 2016). The miR164 mediated *GmNAC1* suppression has also been reported in soybean (Damondaran et al., 2014). However, biological role of miR164:*NAC* modules in salt stress remains *incognito* in soybean. In this study, we identified a set of salt responsive miRNAs, using a sensitive and a tolerant soybean genotype. It was found that four salt stress deregulated miRNAs targeted the *NAC‐TFs* and among these miR164k and miR408d showed antagonistic expression in the two soybean genotypes. It was also shown that the miR164:*NAC‐TF* module was conserved but differentially regulated, in different plant species indicating its dynamic genotype dependent modulation under drought and salt stress conditions.

## 2 MATERIAL AND METHODS

### 2.1 Plant material and stress treatments

Seeds of soybean varieties INCASoy36 (salt tolerant) and William82 (salt sensitive), tobacco cultivar benthamiana and rice variety Pusa basmati 1 were used for this analysis. Seeds were germinated in pots (150 × 150 × 100 mm) containing vermiculate and allowed to grow in greenhouse conditions under a growth regime of 16 hr light/8 hr dark at 23°C°C ± 1°C (Soybean) or 25°C ± 1°C (Tobacco) or 28 ± 1°C (Rice). The seeds were regularly watered with NPK solution (10:10:10).

20 days old seedlings of soybean, rice, and tobacco of uniform age and height were used for the stress treatments. To provide salt stress, seedlings were treated with 200 mM NaCl solution (250 ml per pot). To mimic drought stress, seedlings were treated with solution containing 400 mM Mannitol (250 ml per pot). For all sets the stress treatments were started at same time during light hours. The leaves and roots of four individual seedlings were harvested after 3, 6, 12, 24, 48, and 96 hr of stress and frozen directly into liquid nitrogen. Unstressed healthy plants from the same batch were used as control. For miScript array, 20 days old soybean seedlings were stressed with 200 mM NaCl (250 ml per pot) for 3 hr.

### 2.2 Total RNA isolation

Total RNA was extracted from plant tissues using guanidine isothiocyanate (GITC) based protocol as described in previous reports (Mittal et al., 2013; Sharma et al. 2015). The quality of total RNA was checked by formamide denaturing gel electrophoresis. The concentration was measured using NanoDrop™ 1000 (NanoDrop Technologies).

### 2.3 miScript miRNA PCR Array analysis

336 miRNAs were screened to investigate their expression profiles in two soybean genotypes stressed with 200 mM NaCl for 3 hr. 1 μg total RNA was transcribed to cDNA using miScript Reverse Transcriptase Mix (Qiagen). PCR reactions were carried out in a final volume of 20 μl, containing 1 μl of 1:20 diluted cDNA and miScript Universal Primer. These reactions were performed in 96 well plates in accordance with the manufacturer’s protocol. The expression of miRNAs was detected and quantified in the Step One Plus Real Time PCR (Applied Biosystems). All values were normalized with respect to six endogenous RNAs: *SNORD61, SNORD68, SNORD72, SNORD95, SNORD96A,* and *RNU6B/RNU6‐2*.

### 2.4 Target gene prediction

For *in silico* validation of the cleavage site in transcripts predicted to be targeted by the miRNAs, degradome datasets were analyzed by CleaveLand tool 4.4.3 (Addo‐Quaye et al., 2008). For all further analysis, targets with *p* value ≤ .05 were considered. The predicted targets for differentially expressed miRNAs were analyzed through AgriGO (Tian et al., 2017) to identify the GO groups. Terms with *p* value ≤ .05 were considered for study.

### 2.5 Expression patterns analyses by qRT‐PCR

1μg total RNA was reverse‐transcribed to cDNA with Superscript reverse transcriptase III (Invitrogen), as per manufacturer’s specifications. For miRNA analysis, the specific stem‐loop primers (Table S1) were used while for target analysis oligo (dT) primers were used. The primer sequences of miRNAs and their target genes were designed using Vector NTI Advance 11.0. The reactions were incubated for 30 min at 16°C, followed by pulsed RT of 60 cycles at 30°C for 30 s, 42°C for 30 s, and 50°C for 1s and finally, the reactions were terminated at 70°C for 5 min. All experiments were repeated at least three times.

Stem loop qRT‐PCR was performed in the Step One Plus Real Time PCR (Applied Biosystems, USA) using Universal SYBR Green (Roche, Switzerland) according to the manufacturer’s instructions. The PCR cycle included initial incubation for at 95°C for 15 min, followed by 40 cycles of amplification at 94°C for 15 s, 58°C for 30 s, and 72°C for 30 s. 18S RNA and Actin11 were amplified as reference transcripts to normalize expression of miRNAs and target transcripts respectively. The relative expression levels of miRNAs and the NAC targets were calculated using 2^‐ΔΔCT^ method (Livak and Schmittgen, 2001). Mean of three experimental replicates was plotted and the standard deviation is shown as error bars.

### 2.6 Identification of the NAC‐TFs and their miRNA regulators

*Oryza sativa* (rice), *Nicotiana tabaccum* (tobacco), and *Glycine max* L. (soybean) protein sequences containing NAC domain were identified by PlantTFDB 4.0 (http:// planttfdb.cbi.pku.edu.cn/). The sequences were downloaded and carefully examined to confirm the presence of the NAC or NAM domain using the online domain batch search software, Pfam (http:pfam.sanger.ac.uk). After removal of redundant sequences all hits with E‐value ≤ 1.0 were retrieved. These sequences were used to predict the miRNA directed cleavage site, by analyzing complementary matching, using psRNAtarget server (release 2017) with predefined scoring schema V2 and E‐value ≤ 3 (Dai et al., 2018).

### 2.7 Phylogenetic analysis and gene duplication patterns

Multiple sequence alignment of full‐length NAC protein sequences of monocot and dicot species (rice, tobacco and soybean) was performed using ClustalW2 program with default parameters. A phylogenetic Neighbor‐joining (NJ) tree was plotted using MEGA5.05 software with 1000 bootstrap iterations.

Duplication events in NAC gene were identified using MCScanX software. NAC sequences from soybean and rice were downloaded from phytozome v12.1 and analyzed using an all‐vs‐all local BLASTP algorithm‐based search with e‐value under 1e^‐5^. Top five matches were selected and their blast outputs with coordinates of all protein coding genes were imported into MCScanX software to classify genes in singleton, dispersed, proximal, tandem, and WGD/segmental under a default criterion. Duplication patters analysis could not be performed for tobacco (*TNAC)* genes, as their localization in the chromosomes is not available.

### 2.8 Coexpression assay

The gma‐miR164k precursor and its target *GmNAC1* were amplified with the primers FP‐precursor‐NcoI/ RP‐precursor‐BglII and FP‐gmNAC1‐NcoI/ RP‐gmNAC‐BglII respectively (Table S1). A mutated version of pre‐miR164k (miR164k‐mu) with two altered nucleotides at the cleavage site of mature miRNA was also generated using specific primer sequences (Table S1). The pre‐miRNA and *GmNAC1* target were cloned in the binary vector pCAMBIA1302 under the control of 35S promoter and fused in frame with GFP.

For co‐expression assay, *Agrobacterium tumefaciens* cells containing the binary vector pC1302‐miR164k and pC1302‐miR164k‐mu were individually co‐infiltrated with pC1302‐*NAC:GFP*. Tissues were analyzed for GFP fluorescence after 3 days post‐infiltration (3dpi) using a Nikon spectral confocal microscope (Nikon A1R, Nikon Corporation), with an excitation wavelength of 488 nm. To estimate the accumulation of miR164k and *GmNAC1* transcript semi‐quantitative RT‐PCR was performed. Relative abundance was calculated as Integrated Density Values (%IDV) by normalizing the obtained values with 18S rRNA.

## 3 RESULTS

### 3.1 miScript miRNA array of two contrasting soybean genotypes under salt stress

**Figure 1:**
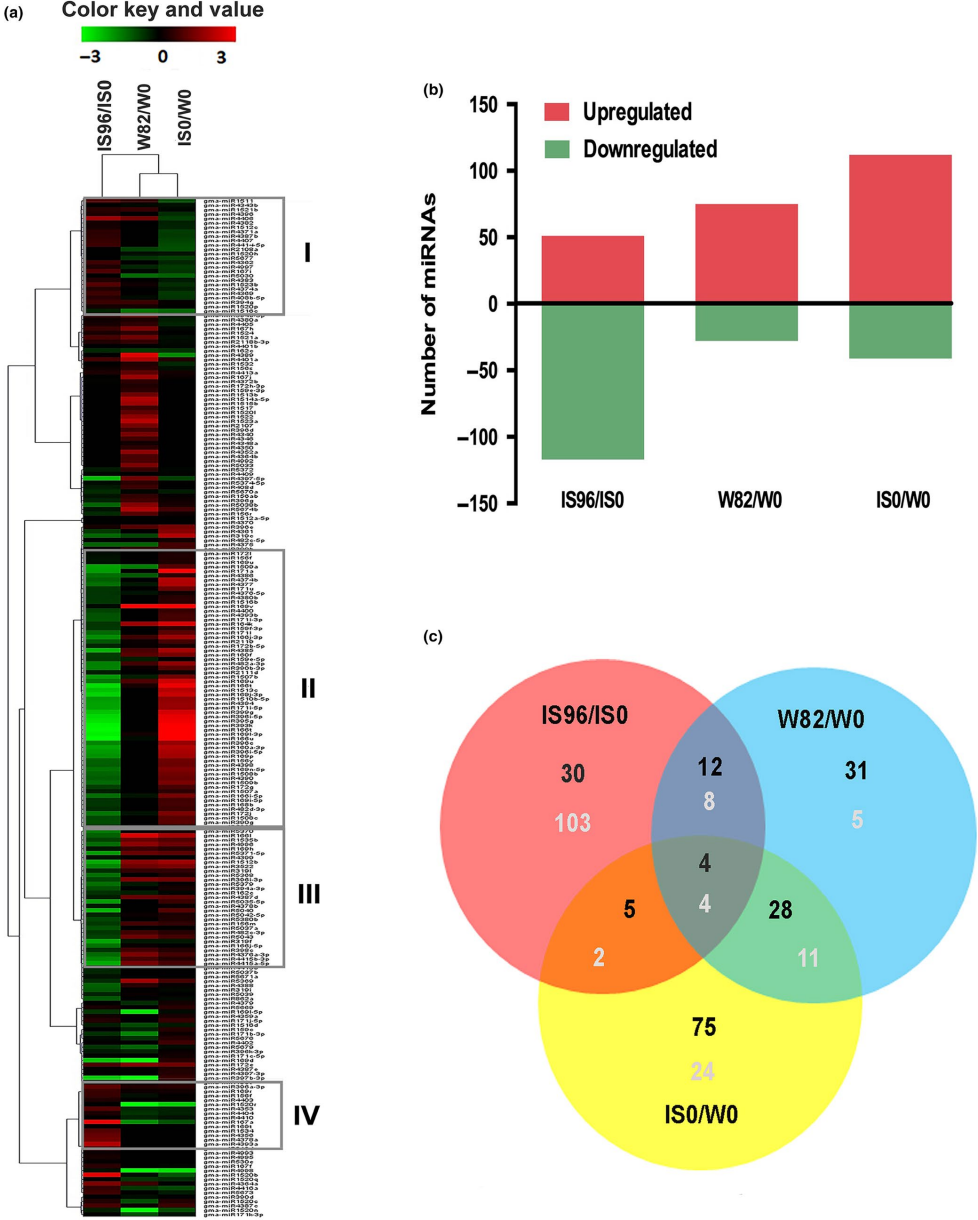
Analysis of salt stress regulated miRNAs. (a) Complete linkage hierarchical cluster analysis of 215 differentially expressed miRNAs in tolerant and sensitive soybean genotypes under salt stress and unstressed control conditions. The salt regulated 135 miRNAs could be divided into four clusters. Log_2_ Ratio is indicated on a color scale from red (high) to green (low). The name of miRNA is given next to each panel The right lane represents comparison of INCASoy36 genotype under salt stress and control (IS96:IS0); the middle lane represents comparison of William82 genotype under salt stress and control (W96:W0) and the left lane represents comparison of both genotypes under control (IS0:W0). (b) Distribution of salt deregulated miRNAs in soybean genotypes as compared to their respective control samples. (c) Venn diagram illustrating the number of unique and overlapping differentially expressed miRNAs in soybean genotypes, as compared with the control; upregulated miRNAs are shown in black color and downregulated in white color

Total RNA from leaves of unstressed (control) and salt stressed 20 day old seedlings of two soybean genotypes, the salt sensitive William82 and salt tolerant INCASoy36 were used to generate the miRNA expression profiles using miScript miRNA array (Figure 1a). To identify miRNAs that were differentially regulated by salt stress, the log_2_ values were calculated and compared with respective unstressed controls of each soybean genotype (INCASoy36 IS96:IS0 and William82 ‐W96:W0). This analysis allowed detection of the differential expression patterns of 215 gma‐miRNAs (Figure S1). These involved 103 miRNAs in William82 and 168 in INCASoy36. It was observed that greater number of miRNAs was downregulated in salt stressed tissues of INCASoy36 as compared to William82 (Figure 1b, Figure S2). Among these expression levels of 51 miRNAs were upregulated and 117 were downregulated in INCASoy36, while expression levels of 75 miRNAs were upregulated and 28 were downregulated in William82 (salt sensitive), under salt stress (Figure 1c). Largely, 55% (183) miRNAs were not expressed in William82 (salt sensitive) while 40% (136) miRNAs were not expressed in INCASoy36 (salt tolerant) genotype (Figure S2) under salt stress. These results indicated that plants have evolved a modulated profile of the salt responsive miRNAs as a part of the adaptation to salt stress.

Expression pattern of 50 miRNAs was not influenced by salt stress in William82 as against 32 miRNAs in INCASoy36 (Figure S2). Interestingly, members of conserved miRNA families whose quantitative expression was not induced (e.g. miR168b, miR171b, miR319d) or not altered (e.g. miR166t, miR390b, miR4362) by salt stress in William82 showed negative regulation in INCASoy36. Similarly, several other miRNAs such as miR156ab, miR169h, and miR482c‐3p showed contrasting expression in both soybean genotypes (Figure 1a, Table S2).

Salt stress induced> 2‐fold difference in the expression of 135 miRNAs, which could be grouped into four clusters based on their expression pattern (Figure 1a). Hierarchical cluster analysis of differentially expressed miRNAs showed that both the number and the degree of expression varied between the two soybean genotypes showing contrasting response to salt stress. Cluster I contained 26 miRNAs that expressed in salt stressed INCASoy36 but showed weak or no expression in salt stressed William82. Cluster II contained 64 miRNAs, which were repressed by salt stress in INCASoy36 but induced in William82. Cluster III contained 31 miRNAs, which were repressed by salt stress in INCASoy36 genotype and not induced or repressed in William82 as well. Cluster IV grouped 14 miRNAs, which showed contrasting expression between INCASoy36 and William82 genotypes.

### 3.2 Targets of the salt regulated miRNAs

To understand the putative regulatory functions of the salt regulated miRNAs, their corresponding targets were searched in the degradome dataset (obtained from MPSS database) using CleaveLand. This analysis identified 714 targets for 215 differentially expressed miRNAs (Table S3), which included transcription factors (TFs), enzymes, transport proteins, carriers etc.

**Figure 2:**
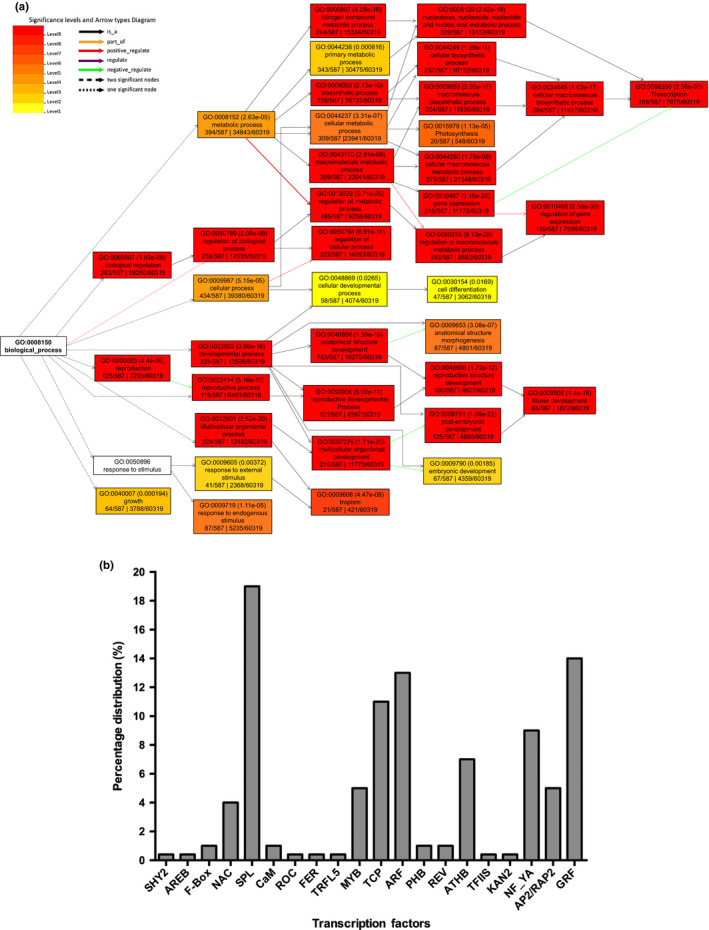
Gene ontology (GO) enrichment analyses of transcripts targeted by salt responsive miRNAs. (a) The most significantly (*p* < .05) enriched GO terms are represented. The color code indicates degree of representation of GO terms and highest representation are showed in red boxes. Specific lines are used to indicate the association of each term. Plant response to stimulus was the most significant biological process related to salt stress regulatory network. (b) Representation of transcription factor (TF) families targeted by salt responsive miRNAs. Transcripts encoding NAC‐proteins constitute 4% of the targeted TFs

To elucidate the gene regulatory networks associated with the miRNA targets GO enrichment analysis was performed. The results showed that transcripts targeted by the differentially expressed miRNAs could be classified into seven biological functions including metabolic process, biological regulation, development process, reproduction, multicellular organisms process, growth and response to stimulus. The response to external and endogenous stimulus emerged as the significant node (Figure 2a), which indicated a possible link to abiotic stress.

Among the targets, there was greater representation of TFs (Table S3), which could be classified into 21 families (Figure 2b). These included transcripts of key regulatory TF families involved in response to salt stress such as, *SPL, GRF* (Growth‐regulating factor), *MYB, NAC, ARF* (Auxin response factor)*, TCP, NF‐YA, ATHB, AP2/RAP2,* and *ZF* (Figure 2b). Among these, members of miR396 family targeted only *GRF‐TFs*, whereas the other TF families were targeted by two or more miRNAs. It was observed that *SPL* (targeted by miR156f,s,t,r,y and miR1516b), *GRF* (targeted by miR396c,e,i‐5p), *ATHB* (targeted by miR166j3p,t,u and miR1515b), *AP2/RAP2* (targeted by miR172e) and *MYB* (targeted by miR72b‐5p, miR169i‐5p, miR159e‐3p,f‐3p) TFs were controlled by salt upregulated gma‐miRNAs (Tables S2, S3). Members of TF families like *ARF, TCP, NF‐YA, ZF,* and *NAC* were modulated by large miRNA families, whose individual members were up or downregulated under salt stress (Tables S2, S3). Even though different members of a miRNA family were predicted to target the same set of transcripts, it is likely that differences in their biological roles may arise due to their varying spatio‐temporal expression patterns. The participation of these TF:miRNA regulatory nodes implied that intricate regulatory mechanisms played an important role in regulation of salt stress tolerance in soybean.

It was found that 4% (nine transcripts) of the identified targets coded for *NAC*‐TFs (Figure 2b). To understand the role of gma‐miRNAs in regulating the expression of the *NAC* transcripts, all the annotated sequences coding for gma‐NAC‐domain containing proteins were obtained. Using *in silico* degradome analysis, the complementary miRNA binding sites were identified in their transcripts. The soybean *NAC* transcripts were found to be targeted by members of four salt stress deregulated miRNAs viz. miR160a‐3p, miR164k, miR408d, and miR1514a (Table 1). The salt regulated miR408d and miR160a‐3p have been reported earlier to target the *NAC*‐TFs (Cakir et al., 2015), even though their target prediction score was very high (e‐value> 4.5).

**Table 1:** Predicted Glycine max NAC transcription factors targeted by miRNAs

miRNA	Target ID	Annotation
miR160a‐3p	Glyma20g33390	ANA053 (Arabidopsis NAC domain containing protein 53)
miR164k	Glyma06g21020	ANAC100 (Arabidopsis NAC domain containing protein 100
	Glyma06g35660	CUC2 (Cup shaped cotyledon 2)
	Glyma12g26190	CUC2 (Cup shaped cotyledon 2)
	Glyma12g35530	CUC2 (Cup shaped cotyledon 2)
miR408d	Glyma02g26480	ATAF1
miR1514a‐5p	Glyma07g05360	NTL9 (NAC transcription factor like‐9)
	Glyma07g05370	NTL9 (NAC transcription factor like‐9)
	Glyma16g01930	NTL9 (NAC transcription factor like‐9)

It was observed that under normal (unstressed) conditions, the expression levels of miR164k and miR160a‐3p were higher while those of miR1514a‐5p and miR408d were lower in salt tolerant INCASoy36 as compared to salt sensitive William82 (Table S2). Under salt stress conditions there was downregulation of miR164k and miR408d in INCASoy36 whereas both miRNAs were upregulated in William82 (Table S2). Salt stress upregulated the expression of miR1514a‐5p and downregulated the expression of miR160a‐3p in both genotypes, however, the levels were higher in William82 (Table S2). This genotype dependent differential behavior of the miRNAs under salt stress indicated an antagonistic regulation of the expression of *NAC* transcripts. Given that, many *NAC*‐TFs positively regulate plant response to stress, the miRNA mediated suppression of William82 NAC transcripts under salt stress might result in its decreased tolerance. Thus, the genetically determined positive and negative miRNA regulations seem to determine the adaptive response of soybean genotypes exhibiting contrasting tolerance to salt stress.

### 3.3 Phylogenetic analysis of conserved NAC‐TFs

To identify the conserved *NAC* transcripts that might be regulated by miRNAs across plant species, 780 sequences representing annotated *NAC* transcripts from soybean (*Glycine max*‐Gm), rice (*Oryza sativa*‐ Os), and tobacco (*Nicotiana tabaccum*‐ Nt) were downloaded from Plant Transcription Factor Database (Jin et al., 2016). These were uploaded to psRNAtarget (Dai et al., 2018) to identify the potential miRNAs that could regulate their expression at the posttranscriptional level. This analysis identified 91 *NAC*‐TFs as targets for the miRNAs (Table S4).

**Figure 3:**
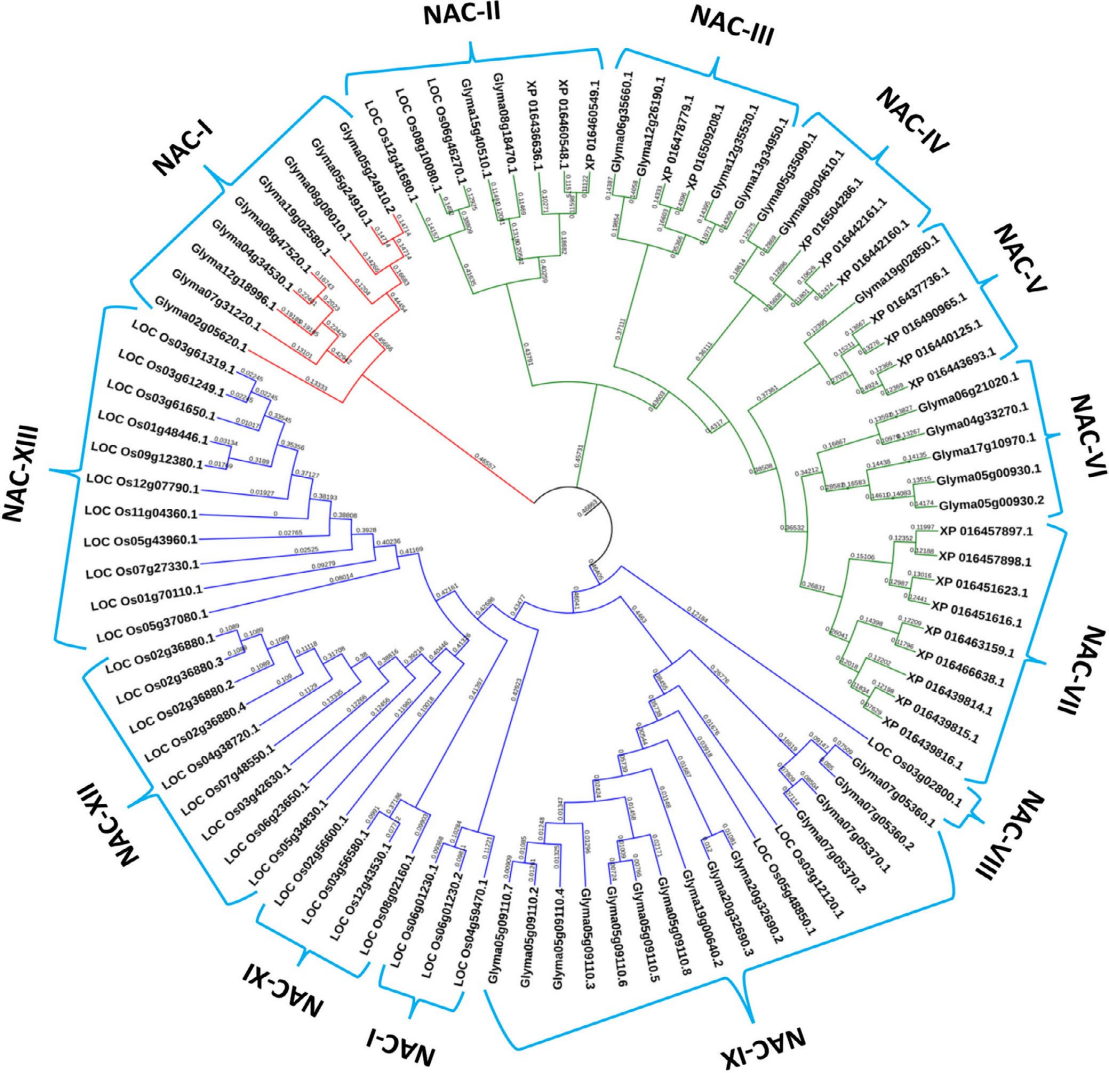
Phylogenetic tree of miRNA targeted *NAC*‐TFs from soybean, rice, and tobacco. Multiple sequence alignment of full‐length *NAC* genes was done using ClustalW2 and the phylogenetic tree was constructed using iTOL v4. These were divided into three main clades, designated as branch lines in red (Clade I), green (Clade II), and blue (Clade III). The numerical values represent branch lengths and internal tree scale

To infer the evolutionary relationship between these sequences, an unrooted phylogenetic tree was constructed. The 91 *NAC* sequences classified into three major groups or clades, which could be further divided into 13 subfamilies (Figure 3). It was seen that *NAC*‐TFs from dicot species showed closer relationship as compared to the monocots. The clade I was composed of only *GmNAC*‐TFs, which may be involved in different development process such as, vascular development (VND1/2), orientation of cell division in root stem cells (FEZ) and root conductivity (XND1) (Tan et al., 2018; Tang et al., 2018; Willemsen et al., 2008). Clade II was most diverse and it included six NAC subfamilies (NAC‐II to NAC‐VII). Interestingly, subfamily *NAC*‐II clustered with the NAC domain‐containing protein 21/22 from all three species and all members emerged as putative targets of miR164. The subfamilies *NAC*‐III to *NAC*‐V contained *GmNACs* and *NtNACs* only and were conspicuous in absence of *OsNAC* members. The subfamilies NAC‐VI and NAC‐VII grouped with members of only *GmNACs* and *NtNACs* respectively. The clade III clustered *GmNACs* and *OsNACs* in six subfamilies (NAC‐VIII to NAC‐XIII). The NAC‐VIII subfamily was integrated by a monocot member and seemed to display divergent expansion of its paralogues and soybean counterparts. Similarly, NAC‐IX subfamily showed high sequence divergence of *OsNAC*‐TFs in relation to *GmNAC*‐TFs. The NAC‐X to NAC‐XIII subfamilies comprised of only *OsNAC* members that were mostly related to the CUC2, NTL9, RD26, and VND2/VND3 proteins (Table S4), indicating that they may perform similar biological functions.

### 3.4 Identification of conserved miRNAs targeting the NAC‐TFs

Target prediction software, psRNAtarget analysis showed that three conserved nta‐miRNAs, 13 gma‐miRNAs, and 14 osa‐miRNAs targeted 21 *NtNAC*‐TFs, 35 *GmNA*C‐TFs, and 33 *OsNAC*‐TFs respectively (Table S5). This analysis also indicated that one miRNA can share sequence complementarity with different *NAC*‐TFs. For instance, osa‐miR5075 was predicted to target 11 analogous *NAC* transcripts. Likewise a single transcript could be targeted by more than one miRNAs e.g. *OsNAC* (Loc_Os01g48446) was targeted by osa‐miR820a‐c and osa‐miR2925 while *NtNAC* (XP_016451616 and XP 016413159) were targeted by nta‐miR164a‐c and nta‐miR396a‐c.

**Figure 4:**
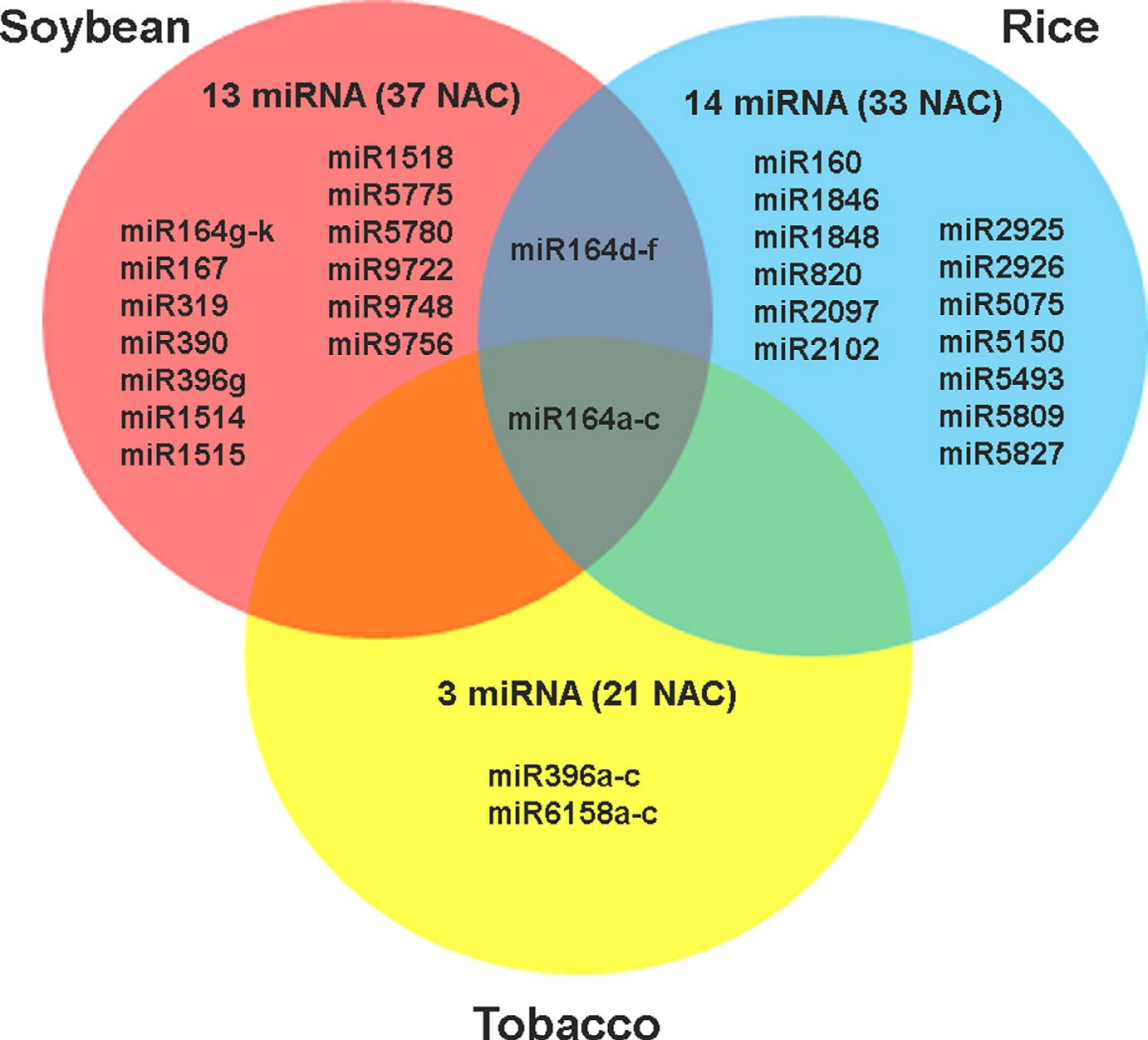
Venn diagram depicting putative miRNAs targeting *NAC*‐TF transcripts. The distribution of conserved miRNAs targeting *NAC* transcripts from soybean, rice, and tobacco, is represented

Interestingly, only miR164 emerged as the common regulator among the studied species (Figure 4). miR164 family consists of 6 members in rice, 3 members in tobacco, and 11 members in soybean. The miR164a‐c was found to target the *NAC*‐TFs in all three species, while miR164d‐f targeted *NAC*‐TFs in soybean and rice. The five members miR164g‐k were specific for *GmNAC*‐TFs (Figure 4).

To gain insight into events that may have been central to the evolution of miRNA regulated *NAC*‐TFs, the mechanisms that generate duplicate gene copies on the chromosomes were investigated. It was found that phylogenetically close *GmNAC* and *OsNAC* transcripts targeted by miRNAs were unevenly distributed across the 20 and 12 chromosomes, respectively (Table S4). Segmental duplications (90%) were identified as the major event in the evolution of *GmNAC*‐TFs, followed by tandem (7%) and dispersed (3%) duplications (Figure S3). Conversely, dispersed duplications (54%) contributed more than segmental duplications (32%) to the expansion of *OsNAC*‐TFs; although to a lesser extent, proximal and tandem duplication also contributed (7%) to their evolution (Figure S3). Two intraspecies homologous pairs (Glyma10g20830.2/Glyma12g18996.1 and Loc_Os03g61319/ Loc03g61249) seemed to be derived by segmental and tandem duplication in soybean and rice, respectively.

### 3.5 Coexpression assay to show that GmNAC1 accumulation is regulated by gma‐miR164k

**Figure 5:**
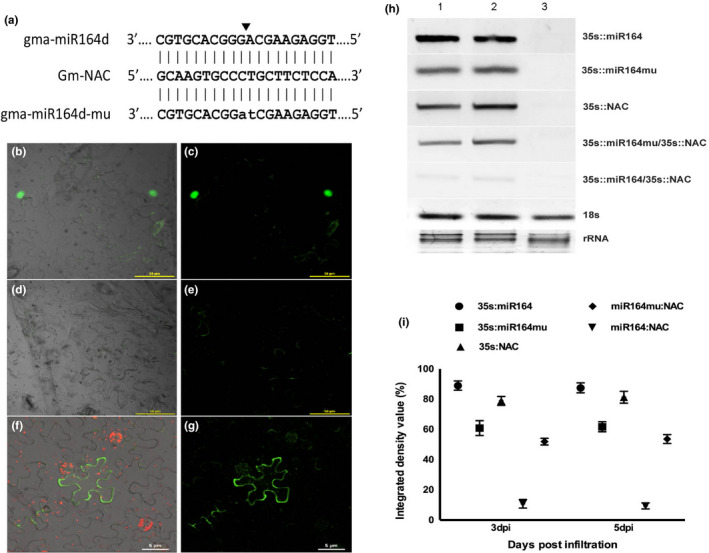
Co‐expression analysis of gma‐miR164k and *GmNAC1* constructs in *N. benthamiana*. (a) Sequences of gma‐miR164k and its target site in *GmNAC1*. The introduced mutation in gma‐miR164mu is represented in lowercase. (b, c) Nuclear localization of *GmNAC1* during interaction with gma‐miR164kmu (mutated version). (d, e) *GmNAC1* during co‐expression with gma‐miR164k. (f, g) Image of a vector control pCAMBIA1302. H) RT‐PCR performed for *GmNAC1*, gma‐miR164k and miR164kmu during co‐expression. The lanes represent samples collected at 1) 3dpi, 2) 5dpi, and 3) uninfiltrated control. Sample name is mentioned on the right side of the image. I) Accumulation of specific transcripts, as estimated by their IDV and normalized using 18S rRNA of tobacco. Error bars represent SD of three independent experiments

To validate that gma‐miR164k directs the cleavage of *GmNAC1* transcript, a transient coexpression assay was performed in *N. benthamiana* leaves. For this experiment three constructs were generated using pCAMBIA1302 and transformed independently in *A. tumefaciens* (Figure 5). The constructs pC1302‐miR164k (expressing miR164k) and pC1302‐miR164k‐mu (expressing mutated miR164k) were respectively coinfiltrated with pC1302‐*NAC:GFP* (NAC1 fused to GFP) in tobacco leaves. After three days post agroinfiltration (3 dpi), the leaves were detached and visualized under confocal microscope. GFP signal showed strong nuclear localization when miR164k‐mu and *GmNAC:GFP* were coexpressed (Figure 5b,c) but no signal was detected when *GmNAC:GFP* was co‐infiltrated with miR164k (Figure 5d,e). This confirmed that GmNAC1 was regulated by miR164k, as non‐functional (mutated) miRNA does not affect GmNAC1 expression. In controls, low intensity of GFP was spread across the nucleus and cytoplasm while brighter intensity was seen at the membrane (Figure 5f,g).

The results were further confirmed by molecular analysis of gma‐miR164k and *GmNAC1* in the co‐infiltrated tobacco leaves. RNA isolated from the co‐infiltrated regions at 3 dpi and 5 dpi were analyzed by RT‐PCR (Figure 5j) and the relative expression was determined after normalizing the band density values with respect to *18S* control (Figure 5k). The individual PCR products were cloned and sequenced for further validation. High levels of *GmNAC1* transcripts were present in leaves co‐infiltrated with miR164k‐mu (Figure 5j). However, co‐expression with gma‐miR164k caused significant decrease of *GmNAC1* expression (Figure 5j,k). The levels of *NAC* transcripts were similar where *GmNAC:GFP* was expressed alone or along with miR164k‐mu (Figure 5k).

### 3.6 Expression analysis of miR164 and its target NAC in soybean, tobacco, and rice

To elucidate whether the functional role of miR164:*NAC* is conserved, comprehensive profiling was performed in soybean, rice, and tobacco. For this analysis, homologs of NAC domain containing protein 21/22, *GmNAC11*, *OsNAC4,* and *NtNAC1* (that clustered together in the NAC subfamily IX, clade II) were selected. The expression of miRNAs and their target were determined by qRT‐PCR, in leaves and roots of 20 days old seedlings subjected to increasing duration of salt and drought stress.

**Figure 6:**
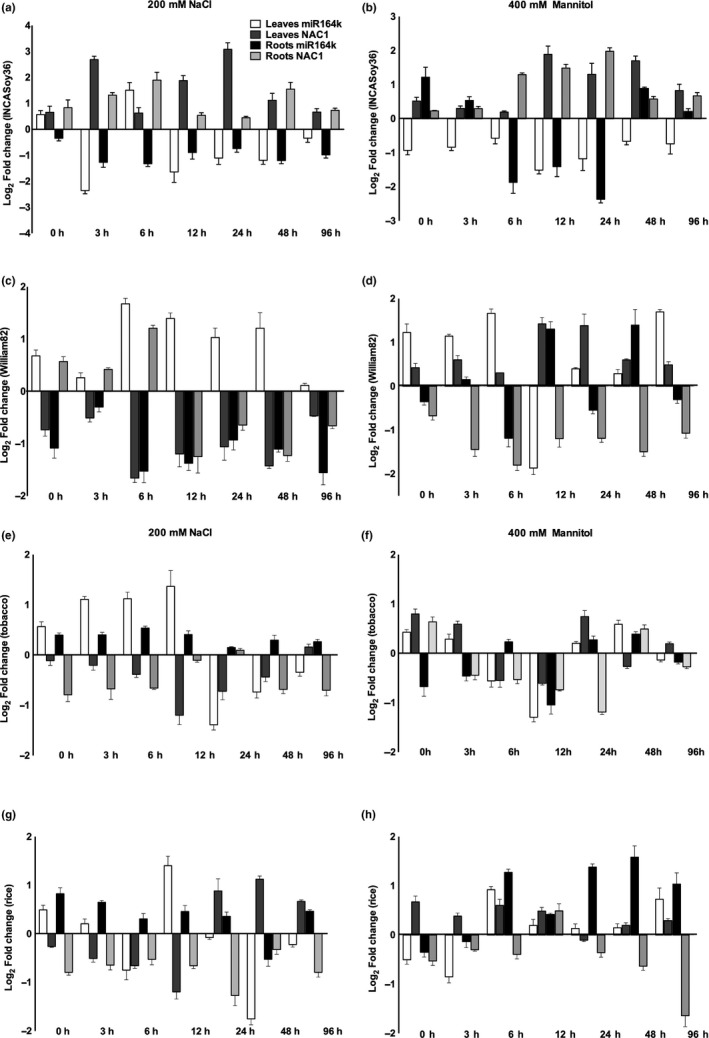
Time kinetics of gma‐miR164 and its target *NAC1* under drought and salt stress. The stress was given to the leaves and root tissues by treating with 200 mM NaCl and 400 mM mannitol, respectively, for 3, 6, 12, 24, 48, and 96 hr. The plot shows fold change of miR164 and its NAC target in INCASoy36 (a, b), William82 (c, d), tobacco (e, f) and rice (g, h) under salt and drought stress. Error bars represent SD of three independent experiments

The expression of gma‐miR164k was downregulated in the salt stressed leaves and roots of INCASoy36 (except for 6 hr salt stressed leaves), while *GmNAC1* showed an opposite pattern indicating its post‐transcriptional regulation by miR164k (Figure 6a). Under drought stress, the levels of miRNA decreased and that of the target increased in the leaf tissues. In the root tissues, the miRNA levels decreased till 24 hr stress and then recovered by 48 hr only to decrease at 96 hr of stress. An opposite pattern was observed for the target in drought stressed roots (Figure 6b).

In salt stressed William82, miR164k levels were upregulated in leaves from 6‐48 hr and downregulated in roots from 6‐96 hr (Figure 6c). The negative relationship was evident in the expression of *GmNAC1* in the leaves and up to 6 hr of stress in the roots. At later time points of stress, the expression of *GmNAC1* was downregulated in roots. Under drought stress, the expression of miR164k was upregulated in leaves till 6 hr but declined sharply at 12 hr and then slowly increased till 96 hr time point (Figure 6d). Whereas its expression in roots was downregulated till 6 hr time point followed by sharp increase from 12‐48 hr after which it dropped a little at 96 hr. The expression of *GmNAC1* showed anti‐correlation in the leaves but in roots this was evident after 12 hr of stress. This demonstrated the existence of additional complexities in regulation of transcripts. Nevertheless, the analysis allowed to capture the narrow window of miR164:*NAC* correlation and indicated an opposite regulation of the module between the salt sensitive and tolerant soybean varieties.

The expression patterns of nta‐miR164 and *NtNAC* in salt and drought stressed tobacco tissues differed from those observed in soybean genotypes. Salt stress triggered the expression of nta‐miR164 in leaves till 12 hr and it was negatively regulated thereafter (24‐96 hr of stress), while *NtNAC* expression showed an opposite expression behavior (Figure 6e). However, in tobacco roots nta‐miR164:*NtNAC* module seemed to remain unresponsive to salt stress. Under drought stress the levels of nta‐miR164 in leaves were reduced except for a small increase seen at 24‐48 hr. Interestingly, *NtNAC* levels did not show inverse correlation under drought stress and seemed to follow a rhythmic pattern of expression (Figure 6f). This suggests that other members of the miR164 and/or *NAC* family may have a role under drought stress in tobacco (Lu et al., 2017).

Rice tissues also showed specific expression pattern of osa‐miR164:*OsNAC* under salt and drought stress. Salt stress downregulated the expression of osa‐miR164 in leaves (except at 12 hr) and roots and expression of *OsNAC* generally showed opposite response (Figure 6g). Whereas after 6 hr of drought stress the expression of osa‐miR164 was induced in the leaves and roots. However, the expression of *OsNAC* showed opposite response only at early (3 hr) stages of drought stress in the leaves and late stages (beyond 24 hr) in roots (Figure 6h). These findings suggest the tissue specificity and dynamic adjustment of osa‐miR164:*OsNAC* module under different stresses.

## 4 DISCUSSION

In this study, expression of 336 miRNAs was evaluated using miScript miRNA array in two soybean genotypes showing contrasting response to salt stress. This identified 215 salt responsive miRNAs, of which 103 were differentially expressed in William82 and 168 in INCASoy36. Hierarchical cluster analysis of salt regulated miRNAs showed that the degree of expression also varied between the two soybean genotypes. Several conserved miRNAs showed contrasting expression in both genotypes and greater number of miRNAs was downregulated by salt stress in INCASoy36 as compared to William82. A significant pool of miRNAs that were negatively regulated in salt stressed INCASoy36 did not show much alteration in expression in William82. Since soybean genotypes show high genetic similarity, the results indicate that differential modulation of miRNAs may have a crucial nodal function in influencing plant adaptation or response to stress.

To elucidate the functional role of miRNAs that were differentially regulated by salt stress, their target transcripts were identified. This revealed a variety of transcripts that are involved in different biological processes related to soybean development. A large fraction of these transcripts encoded TFs and many of these were associated with response to abiotic stress. For instance, it was shown that overexpression of miR172 and miR169 downregulated the transcripts of *AP2* and *NF‐YA* TF families, respectively, to mediate transcriptional and physiological responses of soybean to salt stress (Ni et al., 2013). A role for gma‐miR399 in the regulation of root apical meristem during salt stress was also reported (Sun et al., 2016). Another report suggested the role of miR156 in the regulation of salt stress response by downregulating the *SPL*‐TF (Arshad et al., 2017). Recently it was shown that *ARF*‐TF is negatively regulated by miR160 and miR167 under salt stress. The *ARF* promoter showed enhanced expression of GUS in salt stressed roots, but no change in GUS expression was observed in salt stressed leaves, as compared with controls (Bouzroud et al., 2018). It is thus, apparent that miRNA:target nodes may act individually or synergistically to fine tune gene expression for controlling the adaptive response of soybean to salt stress.

Several reports have indicated that the multifaceted NAC proteins have facilitated the adaptation of terrestrial plants to a wide range of environmental conditions during and after their colonization of land. The versatile role of NAC‐TFs in development and adaptive stress response is well‐demonstrated in several plants species (Massange‐Sánchez et al., 2016; Yoon et al., 2008). In soybean, 269 NACs have been reported and among them 38 were differentially expressed in response to abiotic stress (Le et al., 2011). However, data on functional characterization of soybean miRNA:*NAC* module under salt stress response is scarce. In this study it was observed that salt regulated miR160a‐3p, miR164k, miR408d, and miR1514a‐5p regulate *GmNAC* family members that encode ANAC053, ANAC100, CUC2, ATAF1, and NTL9.

The NAC family has expanded and diversified in angiosperms by gene duplication, which has resulted in multiple copies of genes with high sequence similarity. In this study, 91 *NAC* transcripts from rice, tobacco, and soybean were identified as targets for miRNAs. These were phylogenetically clustered into three different clades comprising 13 subfamilies based on the similarities of their sequences. The close relationships observed in a subfamily may be due to the fact that the genes encoding its members originated by segmental duplication (Pascual et al., 2015;Wu et al., 2016; Zhuo et al., 2018). Members of a subfamily also seemed to share similar biological functions and this indicated sub‐functionalization of gene copies and their retention through purifying selection (Li et al., 2018b; Singh et al., 2018). NAC‐I and NAC‐VI subfamilies contained only the *GmNACs* that have been associated with different aspects of plant development. The subfamily NAC‐IX comprised *GmNAC* genes that encode proteins containing a transmembrane domain NTL9, which is a mediator of osmotic stress responses that affect leaf senescence (Yoon et al., 2008). These genes seem to have been derived by tandem duplications and may have acquired an additional role during evolution in soybean. The NAC‐VII grouped with members of *NtNACs* only. Subfamily NAC‐II included evolutionarily closely related members from all three species, which group mainly with NAC domain‐containing proteins 21/22 that are targeted by miR164 family. It is likely that the conserved posttranscriptional regulation has co‐evolved among the three species and may play an important role in the regulation of different aspects of plant growth, development (Podzimska‐Sroka et al., 2015; Xu et al., 2016a) and abiotic stress response (Fang et al., 2014; Nuruzzaman et al., 2010). On the other hand, *GmNAC* and *NtNAC* transcripts clustered in NAC‐IV subfamily showed sequence similarity with high bootstrap support, indicating that members of this subfamily diverged from a common ancestor. It emerged that *OsNACs* that integrated the abiotic stress‐related functional clusters NAC‐XII and NAC‐XIII showed a relatively low bootstrap value indicating that they may have also undergone duplication events, though the nature of such duplications (disperse, segmental or tandem) could not be determined. Although, it has been referred that tandem duplications may play a key role in the expansion of NAC family in rice (Nuruzzaman et al., 2010), other findings support that dispersed duplication may have contributed to the diversification and evolution of several *OsNAC* gene families (Wang et al., 2011).

Bioinformatics algorithms facilitated the identification of conserved and non‐conserved miRNAs, which could direct cleavage of *NAC* transcripts of tobacco, soybean, and rice. Surprisingly, the number of miRNA targeted *NAC* transcripts constituted a small fraction of the TFs that orchestrate the plant regulatory networks. Combining the results from clustering and GO analysis it was identified that most of the *NAC* subfamilies targeted by miRNAs are primarily involved in developmental process and abiotic stress response. This observation was consistent with previous reports for miRNAs targeting different members of large gene families (Baldrich et al., 2015; Liu et al., 2017; Shi et al., 2017). It was also observed that a single *NAC* transcript could be regulated by two different miRNAs. The miRNA pairs osa‐miR820a‐c/miR2925 and nta‐miR164a‐c/nta‐miR396a‐c targeted analogous NAC transcripts, Os01g48446 and XP016451616/XP016413159 respectively. Recently, it has been reported that the target Os01g48446 is encoded by Al‐sensitive gene (Escobar‐Sepúlveda et al., 2017) indicating that the two miRNAs may be involved in regulating its expression in response to different stresses. This exhibits, existence of fine adjustment of coordination during plant development to achieve accurate regulation of spatio‐temporal gene expression.

Although, miR164:NAC module is highly conserved across species, its regulatory functions may have diverged during the evolutionary split of soybean, rice and tobacco. These species have also undergone whole genome duplications that may have contributed to the expansion and diversification of miR164:NAC gene family. The study of miR319a locus in *Arabidopsis* and its closely related species revealed that it is conserved between *Arabidopsis* species and *Capsella rubella* but is divergent among *A. thaliana* and *Brassica oleracea* (Warthmann et al., 2008). Therefore, transgenic plants of *A. thaliana* overexpressing miR319a from *A. thaliana*, *A. lyrata,* and *Sibara virginica* showed similar aberrant phenotypes like cotyledon epinasty, crinkled leaves and siliques but, overexpression of *B. oleracea* 319a did not display development defects in leaves and flowers. These findings indicated that miR319a module might have diversified their biological functions during the course of evolution of these species. miR164:*NAC* may have followed a similar fate so its expression pattern varies in response to drought and salt stress in different plant species.

The cleavage of *GmNAC1* transcript by gma‐miR164k was validated by transient co‐expression assay performed in *N. benthamiana* leaves. It was observed that co‐infiltration of pC1302‐miR164k (expressing miR164k) with pC1302‐*NAC:GFP* (NAC fused to GFP) did not cause specific accumulation of GFP as the *NAC:GFP* transcript was cleaved by the miRNA. However, co‐infiltration of pC1302‐miR164k‐mu (expressing mutated miR164k) with pC1302‐*NAC:GFP* resulted in GFP accumulation in the nucleus as the *NAC:GFP* transcript could not be cleaved due to mutation in the miRNA. The results were further confirmed by molecular analysis of transcripts in the infiltrated regions. Similar transient assay was used to demonstrate that two members of gma‐miR171 family target *GmSCL‐6* and *GmNSP2* transcripts (Hossain et al., 2019). Co‐expression analysis was also used to confirm the cleavage activity of peu‐miR164 on both PeNAC070 and PeNAC012 (Lu et al., 2017).

The miRNAs can have different mode of action during time course of stress (Diaz et al., 2015; Lopez‐Gomollon et al., 2012; Sharma et al., 2015), thus, temporal distribution of their profiles and positive or negative correlations with the target accumulation can be used to gain insights into their behavior. The miR164:*NAC* module is conserved across rice, tobacco, and soybean, which suggests that several features of the ancestral regulatory pathway and the target gene preference have been retained during the evolution of angiosperms. To determine its functional divergence the dynamic expression patterns of miR164 and its target *NAC*‐TF were captured in soybean, tobacco, and rice tissues under salt and drought stress.

The miR164:*NAC* interaction and accumulation under salt and drought stress differed between the soybean genotypes. Both stresses induced less accumulation of gma‐miR164 targeted *GmNAC15* transcript in the salt tolerant INCASoy36 genotype as compared to the salt sensitive, William82. This result was in agreement with previous studies, which showed that *NAC* expression levels were higher in salt/drought‐tolerant genotypes than in sensitive ones (Hussain et al., 2017; Sun et al., 2018). The finding suggests that miR164:*NAC* module may play an important role in determining the stress tolerance level of a particular genotype. Recently, it has been shown that *GmNAC15* is induced by different abiotic stress like salt, drought, cold and its overexpression in soybean hairy roots enhanced salt tolerance (Li et al., 2018a). There is evidence for the negative effect of OsNAC06 on drought tolerance in rice (Fang et al., 2014), suggesting that osa‐miR164 acts as a positive regulator in response to salt stress, which confirms that miR164:NAC has functionally diverged among the species.

The expression patterns of miR164:*NAC* in salt and drought stressed tobacco and rice tissues differed from those observed in the soybean genotypes. In tobacco leaves nta‐miR164 expression was induced during early stages of salt stress to keep the levels of *NtNAC* suppressed. However, in tobacco roots the miR164:*NAC* module remained unresponsive to salt stress. Under drought stress, *NtNAC* levels seemed to be independent of miR164 regulation indicating a role for other members of the miR164 or NAC family. In salt stressed rice tissues expression of osa‐miR164 was downregulated but in drought stressed tissues the expression of osa‐miR164 was induced. The results indicate dynamic adjustment of osa‐miR164:*OsNAC* module under different stresses in a tissue and species specific manner.

The complexity in genetic regulation of miR164:*NAC* module and their downstream interacting factors in a spatio‐temporal manner, in response to specific stresses, provides a source of genetic diversity among the species and reflects on their adaptability to stress. However, in absence of experimental validations, these findings cannot be directly correlated with the limited tolerance to salinity and drought stress exhibited by the tobacco and rice genotypes (Akita and Cabuslay, 1990; Balyan et al., 2017), used in this study. Differences in the miRNA expression levels and their targets has been reported among different species and even between inbred line, indicating that miRNA:target expression is not strictly conserved (Axtell and Bartel, 2005; Hossain et al., 2019; Khan et al., 2017; Li et al., 2012). It has been shown that under stress conditions the relationship between miRNA and its target is dynamic in different tissues (Oh et al., 2017). The variation in miR164:*NAC* expression in response to salt and drought stress has been reported before, in *Populus* (Lu et al., 2017). The authors observed that expression of miR164 family is higher in populous roots than in leaves under moderate drought stress, whereas the target *NAC70* showed an opposite expression profile. The repression of these *NAC* transcripts in roots may be a recurring strategy to maintain the steady‐state levels to ensure optimum development (Cohen et al., 2006). In fact, the *Arabidopsis* lateral root development requires miR164 mediated attenuation of auxin signals through repression of *NAC1* transcripts (Guo et al., 2005). This phenomenon suggests that miRNAs are involved in regulating complex bidirectional molecular networks and their role may not be limited to the direct repression of target transcripts (Diaz et al., 2015).

## 5 CONCLUSIONS

Thus we can conclude that miRNA:*NAC* target module is operative during adaptive stress response in soybean, rice, and tobacco. Target prediction analysis showed that 13 gma‐miRNAs targeted 37 *GmNAC*‐TFs. In salt tolerant INCASoy36, expression levels of miR164k and miR160a‐3p were higher while those of miR1514a‐5p and miR408d were lower, as compared to those in salt sensitive William82. Under salt stress conditions, there was downregulation of miR164k and miR408d in INCASoy36 whereas both miRNAs were upregulated in William82. Salt stress upregulated the expression of miR1514a‐5p and downregulated the expression of miR160a‐3p in both genotypes, however, the levels were higher in William82. This genotype dependent post‐transcriptional regulation of *NAC* transcripts by the corresponding miRNAs indicates the variation in stress tolerance response exhibited by the two soybean varieties.

Although there was variability in the number of miRNAs and the *NAC* targets, the miR164:*NAC* module appeared to be conserved across species but showed dynamic expression profile under increasing duration of stress. Under stress, profiles were different in soybean, tobacco, and rice. In INCASoy36, the expression of gma‐miR164K was downregulated under salt stress and drought stress in both leaves and roots, while in William82, expression gma‐miR164k was upregulated under salt stress and drought stress in leaves but downregulated in the roots. In stressed tobacco leaves, expression of nta‐miR164 was induced at early time points but negatively regulated at longer durations of stress. The nta‐miR164/Nt*NAC* module seemed unresponsive to salt stress in the roots. The expression of osa‐miR164 in rice leaves and roots was downregulated under salt stress but upregulated under drought stress. Considering the conserved nature of miR164:*NAC* module it is suitable target for future work to understand its intricate regulation at the genome level to decipher the mechanism underlying abiotic stress response.

## AUTHOR CONTRIBUTIONS

YH conducted the experiments, analyzed the results, and wrote the manuscript. KG performed the bioinformatics analysis. NSM conceived the study and edited the manuscript. All authors reviewed the manuscript.

## CONFLICT OF INTEREST

Authors have no conflict of interest to declare.

## Supporting information

FigS1a

FigS1b

FigS2

FigS3

TableS1

TableS2

TableS3

TableS4

TableS5
