# Supplementary figures and images for: Stress induced dynamic adjustment of conserved miR164:NAC module
Source: Plant Environ Interact. 2020 Aug 10;1(2):134–51. doi: 10.1002/pei3.10027 (PMC10168063; doi:10.1002/pei3.10027)

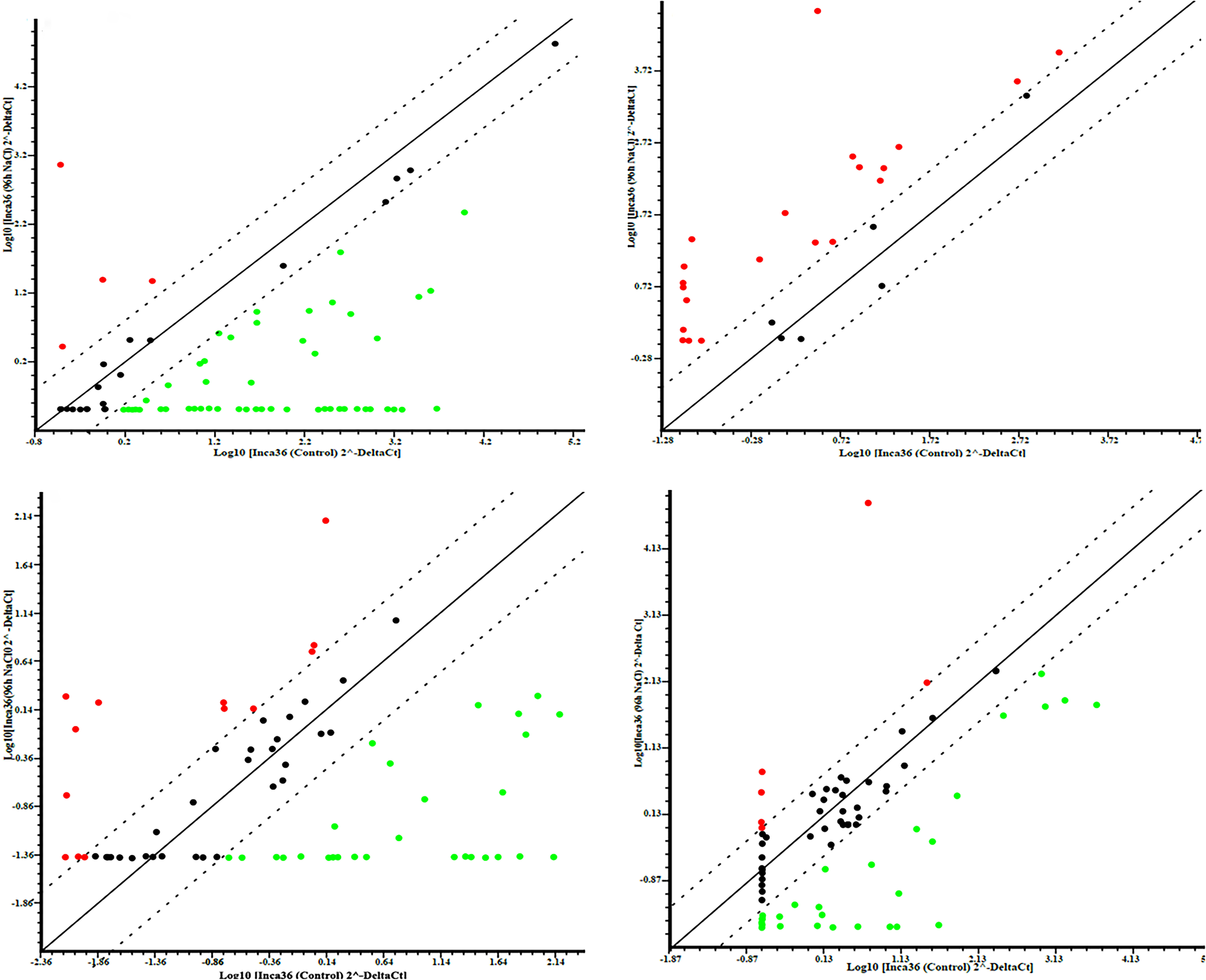

Supplement: Supplementary file 1 — FigS1a [file PEI3-1-134-s008.jpg]

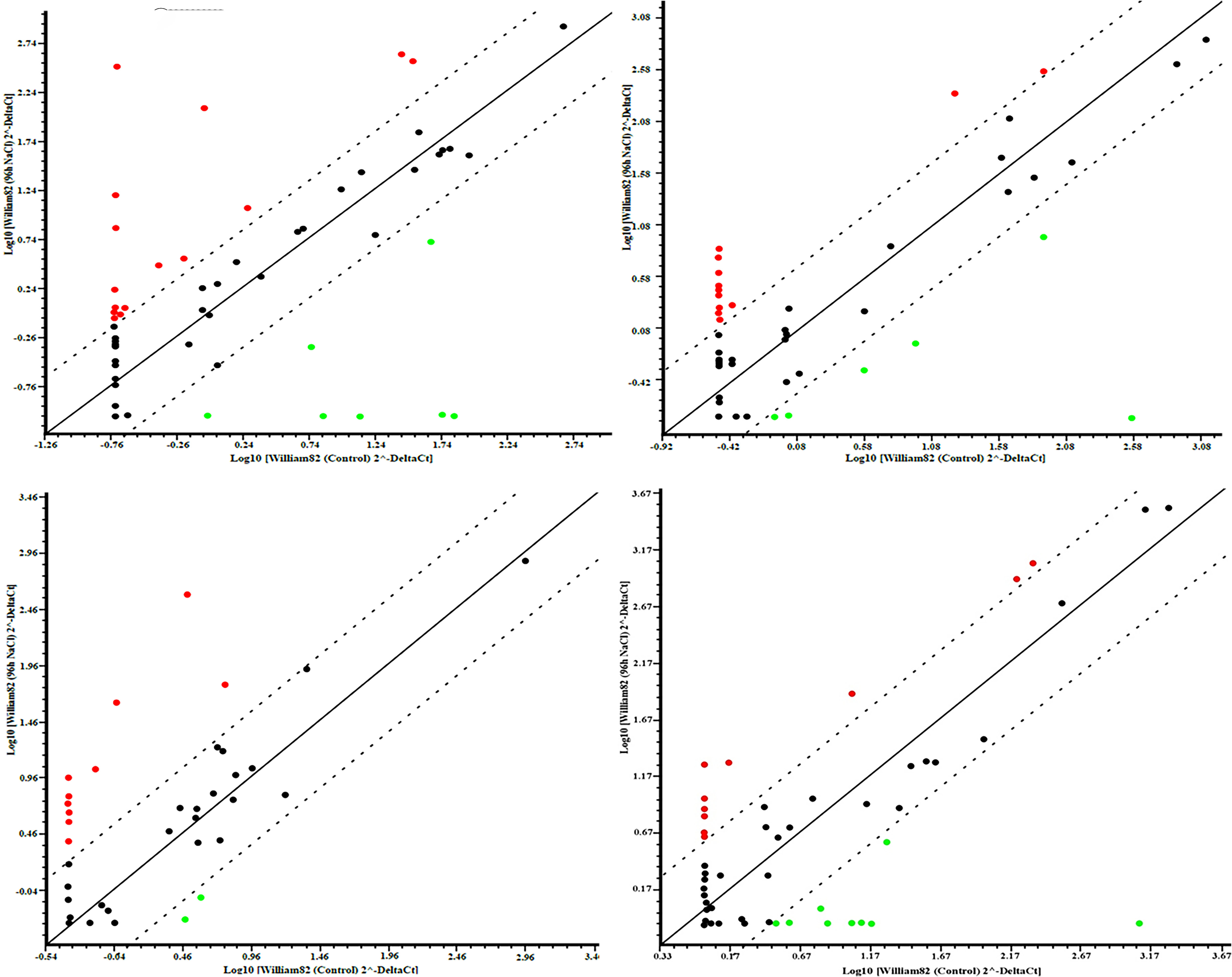

Supplement: Supplementary file 2 — FigS1b [file PEI3-1-134-s007.jpg]

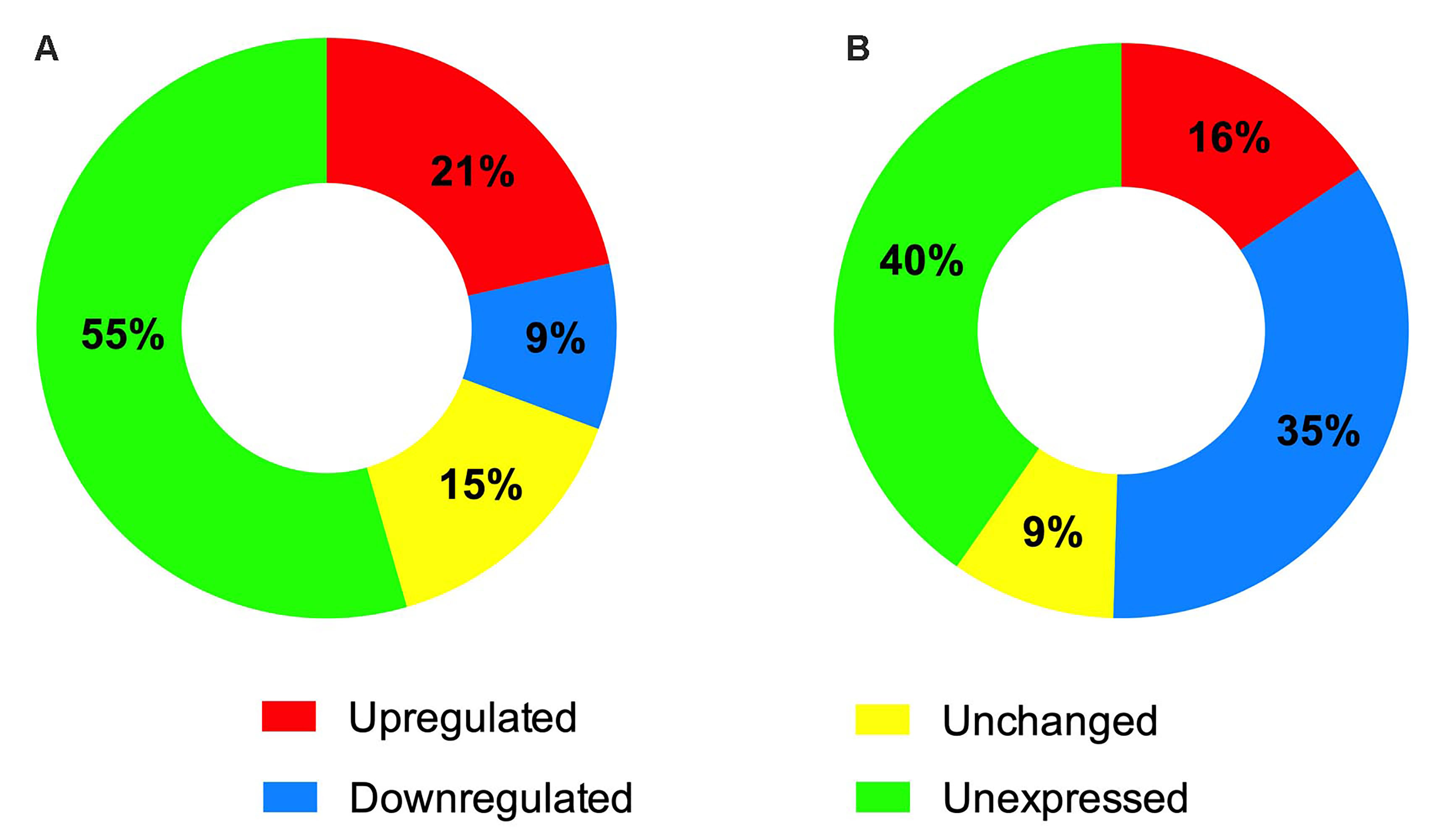

Supplement: Supplementary file 3 — FigS2 [file PEI3-1-134-s006.tif]

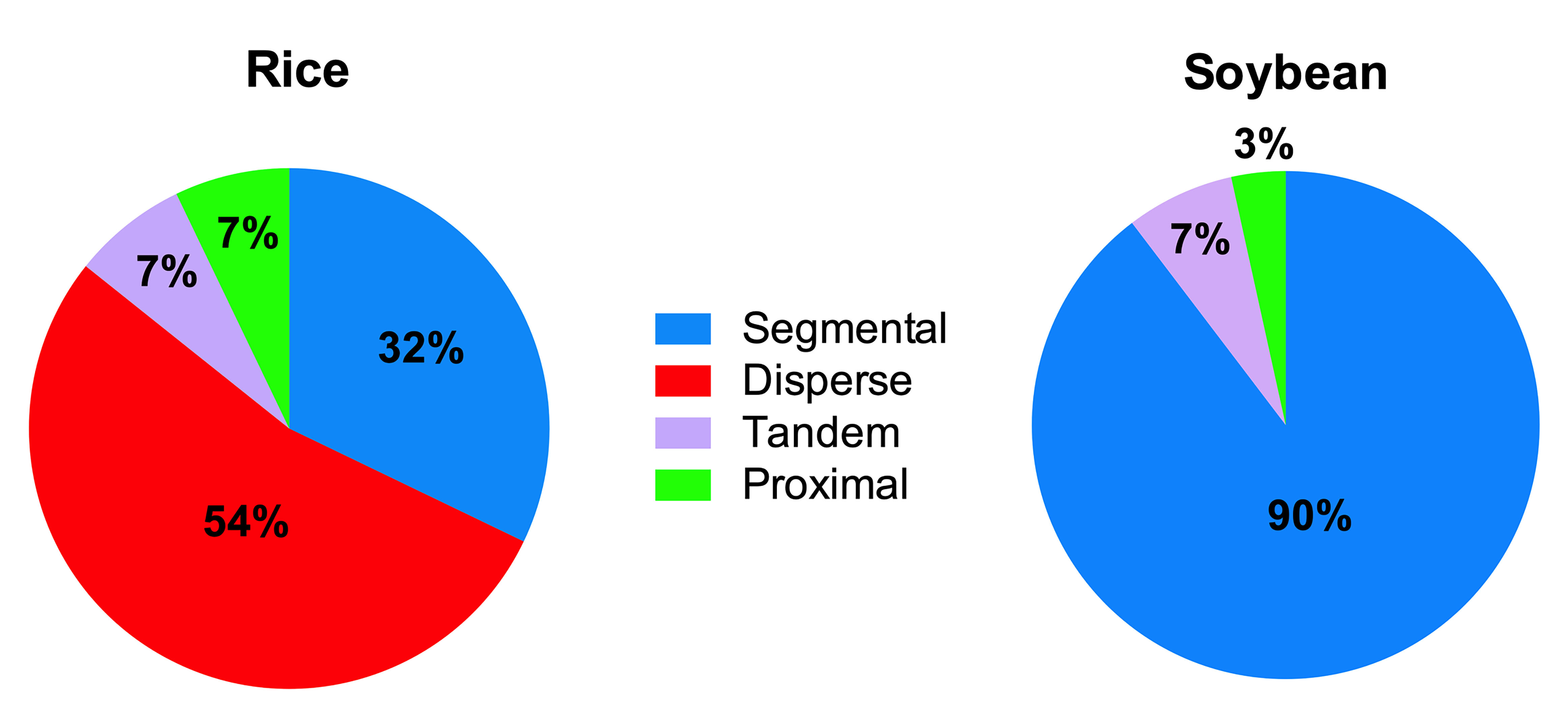

Supplement: Supplementary file 4 — FigS3 [file PEI3-1-134-s005.jpg]
