# Supplementary material for: Stress induced dynamic adjustment of conserved miR164:NAC module
Source: Plant Environ Interact. 2020 Aug 10;1(2):134–51. doi: 10.1002/pei3.10027 (PMC10168063; doi:10.1002/pei3.10027)
Supplement: Supplementary file 5 — TableS1 [file PEI3-1-134-s001.pdf]

**Table S1 Primer sequences of miRNA and their targets used for qRT-PCR.**

| miRNA Name | miRNA primers (5'→ 3') |
|---|---|
| miR164d RT | GTCGTATCCAGTGCAGGGTCCGAGGTATTCGCACTGGATACGACGCACGT |
| miR164d FP | CACGCATGGAGAAGCAGG |
| osa-miR164d RT | GTCGTATCCAGTGCAGGGTCCGAGGTATTCGCACTGGATACGACAGCACG |
| osa-miR164d FP | CACGCATGGAGAAGCAGG |
| nta-miR164b RT | GTCGTATCCAGTGCAGGGTCCGAGGTATTCGCACTGGATACGACTGCACG |
| nta-miR164b FP | CACGCATGGAGAAGCAGG |
| gma-18s FP | GTCCCTGCCCTTTGTACA |
| gma-18s RP | CACTTCACCGGACCATTC |
| osa-18s FP | CTACGTCCCTGCCCTTTGTACA |
| osa-18s RP | ACACTTCACCGGACCATTCAA |
| nta-18s FP | GATCAGATACCGTCCTAGTC |
| nta-18s RP | CCCGGAACCCAAAAACTTTG |
| **Target gene Name** | **Target gene primers (5'→ 3')** |
| GmNAC1 FP | CAAGTGTGAGCCATGGGATA |
| GmNAC1 RP | GTGGTTGAAAATGGGGTTTG |
| OsNAC4 FP | ATAAGCCGGAAAGGATTGCT |
| OsNAC4 RP | TCCCTCTTCACATTGCCTTC |
| NtNAC1 FP | GATTGGGTGTTATGCCGAGT |
| NtNAC1 RP | GGTTTTCCTCATTCCCACAA |
| gma-Actin11FP | CGGTGGTTCTATCTTGGCATC |
| gma-Actin11RP | GTCTTTCGCTTCAATAACCCTA |
| osa-Actin11 FP | CAGCCACACTGTCCCCATCTA |
| osa-Actin11 RP | AGCAAGGTCGAGACGAAGGA |
| nta-Actin FP | CCACACAGGTGTGATGGTTG |
| nta-Actin RP | CACGTCGCACTTCATGATCG |
| **Cloning primer** | **Sequence primers (5'→ 3')** |
| FPprecursor-NcoI | TATCCATGGTTAACTCCTGTTGGAGAA |
| RPprecursor-BglII | CCAAATAGATCTGGAATGAGGTAGGAA |
| FPprecursorNcoI-mut | ATCCATGGTTAACTCCTGTTGGAGAAGCCTAG |
| RPprecursorBglII-mut | GGAAGATCTTGTTGGAGTTGGGATGCACGT |
| FPgmNAC1 NcoI | ACCATGGGCAACATAAGCATGGTAGAGG |
| RPgmNAC1 BglII | AAGATCTAAAGTCTCACATCAGTAATTCCA |
