# Supplementary material for: Stress induced dynamic adjustment of conserved miR164:NAC module
Source: Plant Environ Interact. 2020 Aug 10;1(2):134–51. doi: 10.1002/pei3.10027 (PMC10168063; doi:10.1002/pei3.10027)
Supplement: Supplementary file 6 — TableS2 [file PEI3-1-134-s002.pdf]

Table S2 List of DE miRNAs in the tolerant and sensitive genotype under salt stress

Name	W96/W0	IIS0/W0	IS96/IS0
gma-miR159e-3p	1.373931503	0	0
gma-miR168b	0	3.436643982	-2.696643982
gma-miR160f	2.632518387	5.990478897	-2.95450798
gma-miR156y	0	5.59977951	-4.85977951
gma-miR164k	4.688512421	11.01437225	-2.658939514
gma-miR169u	0	1.638006592	-0.898006592
gma-miR160a-3p	0	9.013277435	-8.273277435
gma-miR172b-5p	0.213632202	1.385168457	-1.934705887
gma-miR169v	10.21289978	18.95855751	-0.533250961
gma-miR171a	0.158223724	14.34971466	-7.862766418
gma-miR166t	0	13.31744995	-12.57744995
gma-miR396a-3p	0.972566223	2.203287506	4.839753952
gma-miR396i-5p	0	8.439909363	-7.699909363
gma-miR399g	0.13371048	10.59824409	-9.858244095
gma-miR159f-3p	-0.000856781	2.384437943	-2.747373734
gma-miR156m	2.064157104	2.518062973	-2.894119415
gma-miR399h	1.667977905	3.231813812	0.903135147
gma-miR156f	0	1.610186005	-0.870186005
gma-miR166c	0.794889069	11.94571915	-11.20571915
gma-miR166u	-0.37133255	14.57749977	-13.83749977
gma-miR167a	-4.244964981	-4.290195084	11.91336426
gma-miR394a-3p	-0.046509171	1.283113861	-3.934328232
gma-miR394g	1.764863586	0.420267487	3.143445816
gma-miR396k-3p	-1.013822937	0.481508636	-1.225862656
gma-miR396i-5p	0	11.66730537	-10.92730537
gma-miR169d	-10.19360771	1.955055618	-9.75866333
gma-miR169r	0.480210876	1.029364014	2.863313522
gma-miR390g	0	4.533831024	-3.793831024
gma-miR393k	0	13.00270882	-12.26270882

Name	W96/W0	IIS0/W0	IS96/IS0
gma-miR4377	-0.71803421	8.673768845	-5.635830383
gma-miR4378a	0	0	5.621589203
gma-miR4378b	0	0.54507431	-1.18507431
gma-miR4379	-1.309758606	1.016170349	-0.160591583
gma-miR4380a	0.867765961	-1.401821289	0.761821289
gma-miR4380b	0	1.963916626	-2.603916626
gma-miR4382	0	-1.67921463	1.03921463
gma-miR4383	0.052099762	-1.247749481	1.819005508
gma-miR4385	2.219393311	8.419212189	-9.059212189
gma-miR4386	-2.649646225	2.31479248	-5.483896713
gma-miR4387b	0	-2.914573822	2.274573822
gma-miR4387c	0.471102295	3.031389084	3.658551712
gma-miR4387d	3.06204277	4.754175034	-1.482470016
gma-miR4388	1.178804932	0.469123688	-4.837006073
gma-miR4389	6.645096359	-7.03480545	0
gma-miR4390	-0.078824463	5.156011429	-4.652578812
gma-miR4393a	0	0	9.037343521
gma-miR4393b	0	3.471978989	-4.111978989
gma-miR4394	0	7.502197113	-8.142197113
gma-miR4396	0	-2.252853546	1.612853546
gma-miR4397-3p	0	1.892749634	-0.119091492
gma-miR4399	0	0.245780792	-0.885780792
gma-miR4400	0	2.675618973	-3.315618973
gma-miR4401a	5.113621292	1.795951691	3.443442841
gma-miR4402	0	3.247230377	0.342324753
gma-miR4403	0.058039246	0.362611618	1.504893799
gma-miR4404	-1.441026154	-1.696151886	1.461356659
gma-miR4405	1.391184387	-1.755241547	1.197358627
gma-miR4406	3.330532608	-3.210994873	7.86967041

gma-miR396c	0	7.530580902	-6.790580902
gma-miR482c-5p	0	1.093088531	-0.353088531
gma-miR159e-5p	-0.892538452	0.37649765	-2.948547516
gma-miR162a	-0.89092865	-0.341577148	-2.407419357
gma-miR166l	6.561193085	8.455467606	-4.791322861
gma-miR169p	0	8.083710098	-7.343710098
gma-miR169i-5p	0	3.31802597	-2.57802597
gma-miR171c-5p	0	0.874185944	-0.134185944
gma-miR395g	0	12.10972443	-11.36972443
gma-miR4376-5p	-0.561897659	0.927183533	-2.530204926
gma-miR482a-3p	2.034804916	7.261341476	-6.521341476
gma-miR482d-3p	0.049371338	2.98745575	-2.24745575
gma-miR1520n	-7.788338089	-3.832999802	-1.565338287
gma-miR159c	-1.063570404	2.105884933	0.151796188
gma-miR162c	-1.723055267	-2.405818558	-2.057796631
gma-miR166j-5p	0.373340225	0.129171753	-5.708410416
gma-miR166i-5p	-0.601631546	5.944239044	-5.582716141
gma-miR166j-3p	1.872090912	7.870270157	-7.130270157
gma-miR169h	1.685054398	2.122122192	-1.23868576
gma-miR169l-5p	-6.858248138	-0.59548912	-2.70470253
gma-miR169l-3p	0.593412018	13.9590992	-12.02017799
gma-miR169n-5p	0	6.454832458	-5.714832458
gma-miR169t	0	0	0.13044342
gma-miR169c	2.328620529	10.27919617	-6.020750198
gma-miR171b-3p	-3.982607269	0.817804718	-2.410411987
gma-miR172l	0	1.396444702	-0.656444702
gma-miR2119	0.380521393	3.635839844	-4.366039429
gma-miR319c	-2.040264511	9.069317245	-5.289753113
gma-miR390b-3p	0.339343643	4.40177002	-6.638012085
gma-miR390d	-0.42469635	0	0
gma-miR397b-3p	-9.882793808	2.774334335	-10.26712814
gma-miR4398	0	5.435713196	-4.695713196
gma-miR482c-3p	1.935227966	2.126650238	-2.66863266

gma-miR4407	0	-3.336559448	2.696559448
gma-miR4409	0	-0.216165695	-0.423834305
gma-miR4410	-0.992230835	-0.954136047	0.343062897
gma-miR4413a	2.78303772	1.124547806	1.5341716
gma-miR4414-5p	0	-3.781021271	3.141021271
gma-miR4415a-5p	3.522974548	3.699457016	-8.591558914
gma-miR4416a	-1.695302429	-3.732957993	2.518624802
gma-miR1510b-5p	0	7.152662125	-7.792662125
gma-miR1512b	4.12120491	8.044229355	-8.684229355
gma-miR1512c	0	-1.838569794	1.198569794
gma-miR1513c	0	9.040708389	-9.680708389
gma-miR1516b	0	2.150224533	-2.790224533
gma-miR1521b	1.568867264	-0.323110733	2.065255661
gma-miR1523b	0.51772934	-2.331583176	4.326020737
gma-miR1535b	3.057980118	4.07507309	-3.269783478
gma-miR167h	3.695152817	-0.441259537	2.9147258
gma-miR167i	-0.222203674	-2.669120941	4.089120407
gma-miR169j-3p	0	10.02309212	-10.66309212
gma-miR171u	0	5.303516235	-2.722477417
gma-miR171i-3p	0	3.594714966	-4.234714966
gma-miR171i-5p	0	7.570419159	-8.210419159
gma-miR171j-5p	0	2.795112457	1.922614594
gma-miR171l	0	4.584100571	-5.224100571
gma-miR172g	0	3.649841156	-3.200333099
gma-miR172j	-0.559375229	6.215585556	-5.917234879
gma-miR2111d	0	0.920699921	-1.560699921
gma-miR2118b-3p	1.534250793	-0.470024261	1.712939758
gma-miR319d	0	0	4.024143982
gma-miR319f	-0.685307465	-1.175870819	-8.298302841
gma-miR319l	1.549376526	2.51214035	-2.321858597
gma-miR319i	0	-0.415429993	-4.454570007
gma-miR3522	3.197913208	6.161726074	-6.740336609
gma-miR396g	1.948042908	0.774477081	-1.840830994

gma-miR1507a	0	2.275076294	-1.535076294
gma-miR1507b	-0.954239273	5.734287643	-7.707103882
gma-miR1508c	0	4.416101837	-3.676101837
gma-miR1508b	0	5.834431076	-5.094431076
gma-miR1509a	-3.762194061	4.386543655	-8.197914276
gma-miR1509b	0	6.141357803	-5.401357803
gma-miR1511	1.4359478	-3.693035736	3.854205475
gma-miR1512a-5p	0	0	0
gma-miR1513b	3.247545624	0	0
gma-miR1514a-5p	5.18868866	0	0
gma-miR1515b	4.709522629	0	0
gma-miR1517	1.752264404	0	0
gma-miR1520b	-0.436086273	-4.325995102	11.59918533
gma-miR1520c	-2.333406067	1.782372818	1.844153748
gma-miR1520h	-0.4956604	-1.0356604	0
gma-miR1520l	2.747156525	0	0
gma-miR1520p	0.725469971	0.237906799	1.232093201
gma-miR1520q	-1.561929321	-2.101929321	0
gma-miR1520r	-9.968928909	-10.50892891	0
gma-miR1521a	3.353569412	-0.715763702	3.633197174
gma-miR1522	4.168613815	0	0
gma-miR1523a	5.473339462	0	0
gma-miR1524	1.371576691	0.088761673	1.428437576
gma-miR1532	1.020052338	-2.025757446	0
gma-miR1534	0	0	4.192313538
gma-miR156d	-0.491799927	0.319190369	2.758658752
gma-miR167j	4.588570023	0.521676407	0.133853302
gma-miR167f	0.1408638	-0.389004364	2.274545059
gma-miR172e	3.981056595	5.590903625	3.992005692
gma-miR2107	3.475819016	0	0
gma-miR2108a	-1.957816696	-3.957201614	0
gma-miR394b-3p	3.151348495	-0.727266922	2.709308014
gma-miR396d	1.939836884	0	0

gma-miR396i-3p	3.783783951	5.671165543	-3.519519043
gma-miR398c	1.953391113	1.761560516	-2.534733963
gma-miR408d	1.832869568	0	-1.301263046
gma-miR408b-5p	-0.339776001	-2.523084564	1.975304413
gma-miR4372b	1.401846924	0	-0.256716919
gma-miR4376a-3p	3.672331848	3.499448853	-5.312201691
gma-miR4387e	0	1.62026413	0
gma-miR4397-5p	3.393342056	-3.220649643	-9.407637787
gma-miR4401b	0.045353928	-0.398433609	0.049097824
gma-miR4413b	0	0	-1.704827499
gma-miR4415b-3p	2.000037231	1.85740097	-6.72740097
gma-miR4992	2.251921692	0	0
gma-miR4993	0	0	0.758126068
gma-miR4995	0	0	1.220749664
gma-miR4996	4.206254044	5.455068665	-5.462411118
gma-miR4997	-1.138348541	-2.848348541	1.527893829
gma-miR4998	-9.759379349	-11.46937935	-0.817874146
gma-miR5030	-3.176896057	-4.886896057	0
gma-miR5033	3.696229019	0	0
gma-miR5035-5p	0	2.956754761	-7.826754761
gma-miR5037a	0	0.240144806	-4.021315765
gma-miR5037b	0	0	-1.950040054
gma-miR5037c	-0.412243805	2.043291168	-2.472838593
gma-miR5038b	3.916218796	1.26038559	-5.149510574
gma-miR5039	0.281006851	0.087411957	-1.788405609
gma-miR5040	0	4.349832611	-9.219832611
gma-miR5042-5p	0.431033173	1.425052719	-1.893084717
gma-miR5043	3.223227539	3.459627228	-3.204698753
gma-miR530e	0	0	0.441307831
gma-miR5368	0	1.4004422	-5.671723557
gma-miR5369	4.68630127	3.629980164	-2.119847488
gma-miR5370	0	1.197683411	-6.067683411
gma-miR5371-5p	3.097659149	4.6475354	-7.846921158

gma-miR396e	2.471880341	5.758283005	1.250361786
gma-miR4340	3.594633484	0	0
gma-miR4343b	-0.265309906	-0.805309906	0
gma-miR4346	1.88475647	0	0
gma-miR4348a	0.588323975	0	0
gma-miR4350	1.301203156	0	0
gma-miR4352a	4.255838776	0	0
gma-miR4353	-1.197717285	-1.737717285	4.325736389
gma-miR4356	0	0	5.103492126
gma-miR4359a	-0.107204056	1.34830986	0.854016647
gma-miR4361	0	6.626244888	-2.809305801
gma-miR4362	0.024404907	-1.887091293	3.202336655
gma-miR4364a	1.837602997	0	6.310004578
gma-miR4364b	2.170832062	0	0
gma-miR4369	0.088528214	-2.801994476	3.227023621
gma-miR4370	0	0	0
gma-miR4371a	0	-2.97035614	2.33035614
gma-miR4374a	0	-1.530506287	1.719012756
gma-miR4374b	0	8.994058456	-4.702438812
gma-miR4375	-2.907037201	3.483671036	-4.93201683

gma-miR5372	1.663044968	-0.299648209	-1.423394394
gma-miR5374-5p	1.333216705	0.257688599	-0.581818771
gma-miR5379	-0.69116684	0.547048645	-5.567338181
gma-miR5380b	0	1.564703064	-3.588896942
gma-miR5669	0.179854431	2.108120041	1.337942886
gma-miR5670a	-0.196560822	-1.379474564	-2.249135208
gma-miR5671a	0	0	-0.893714142
gma-miR5673	0	-1.443994446	3.222672272
gma-miR5674b	5.212227859	3.370578842	-1.036133957
gma-miR5676	-1.39456459	1.769788818	-0.590018463
gma-miR5677	-1.472096405	-3.182096405	0
gma-miR5679	-2.950100861	-0.610027237	-2.649047089
gma-miR862a	0	-0.454679413	-4.415320587
gma-miR1516c	-3.421103439	-5.131103439	-1.652489853
gma-miR1516d	-2.202164612	-1.221868439	-1.489094925
gma-miR156ab	1.458857574	0.436441498	-1.037005615
gma-miR156t	0	1.520937042	-0.04866333
gma-miR156s	1.346930542	-0.752500458	0.790133286
gma-miR156r	2.559365311	0.91049202	-2.075947952
gma-miR171k-3p	-2.374001465	-0.926357193	-0.068377686
