# Supplementary material for: Stress induced dynamic adjustment of conserved miR164:NAC module
Source: Plant Environ Interact. 2020 Aug 10;1(2):134–51. doi: 10.1002/pei3.10027 (PMC10168063; doi:10.1002/pei3.10027)
Supplement: Supplementary file 7 — TableS3 [file PEI3-1-134-s003.pdf]

**Table S3 miRNA targets identified by in silico degradome analysis**

| miRNA | Transcript | Pfam | KEGG EC: | KEGG_pathways | Arabidopsis best hit | Transcript annotation |
|---|---|---|---|---|---|---|
| gma-miR5036 | Glyma01g02950.1 | PF00153 | | | AT2G22500.1 | UCP5 (UNCOUPLING PROTEIN 5); binding |
| gma-miR1523a | Glyma01g03720.6 | PF00484 | 4.2.1.1 | K01673 | | |
| gma-miR171i-3p | Glyma01g18100.1 | PF03514 | | | AT4G00150.1 | scarecrow-like transcription factor 6 (SCL6) |
| gma-miR4415a-5p | Glyma01g20710.1 | PF07690,PF00854 | | | AT1G68570.1 | proton-dependent oligopeptide transport (POT) family protein |
| gma-miR171a | Glyma01g33270.1 | PF03514 | | | AT4G00150.1 | scarecrow-like transcription factor 6 (SCL6) |
| gma-miR171k-3p | Glyma01g33270.1 | PF03514 | | | AT4G00150.1 | scarecrow-like transcription factor 6 (SCL6) |
| gma-miR396i-5p | Glyma01g34650.1 | PF08879,PF11722 | | | AT2G45480.1 | AtGRF9 (GROWTH-REGULATING FACTOR 9); transcription activator |
| gma-miR171u | Glyma01g38360.1 | PF03514 | | | AT4G36710.1 | transcription factor |
| gma-miR172l | Glyma01g39520.1 | PF00847 | | K09284 | AT4G36920.1 | AP2 (APETALA 2); transcription factor |
| gma-miR172e | Glyma01g39520.2 | PF00847 | | K09284 | | |
| gma-miR396e | Glyma01g44470.1 | PF08880,PF08879 | | | AT3G13960.1 | AtGRF5 (GROWTH-REGULATING FACTOR 5); transcription activator |
| gma-miR390d | Glyma01g44730.1 | PF04570 | | | AT3G22550.1 | senescence-associated protein-related |
| gma-miR4403 | Glyma02g00260.1 | PF02309 | | | AT1G04240.1 | SHY2 (SHORT HYPOCOTYL 2); transcription factor |
| gma-miR396e | Glyma02g02940.1 | PF00010 | | | AT1G59640.1 | ZCW32; DNA binding / transcription factor |
| gma-miR393k | Glyma02g07240.1 | | | | AT3G26810.1 | AFB2 (AUXIN SIGNALING F-BOX 2); auxin binding / ubiquitin-protein ligase |
| gma-miR172h-3p | Glyma02g09600.1 | PF00847 | | | AT2G28550.1 | RAP2.7 (RELATED TO AP2.7); DNA binding / transcription factor |
| gma-miR398c | Glyma02g10710.1 | PF00479,PF02781 | 1.1.1.49 | K00036 | AT5G13110.1 | G6PD2 (GLUCOSE-6-PHOSPHATE DEHYDROGENASE 2); glucose-6-phosphate dehydrogenase |

| | | | | |
|---|---|---|---|---|
| gma-miR156s | Glyma02g13370.1 | PF03110 | AT1G69170.1 | squamosa promoter-binding protein-like 6 (SPL6) |
| gma-miR156f | Glyma02g13370.1 | PF03110 | AT1G69170.1 | squamosa promoter-binding protein-like 6 (SPL6) |
| gma-miR862b | Glyma02g16810.1 | PF00855 | AT3G63070.1 | PWWP domain-containing protein |
| gma-miR394b-3p | Glyma02g18090.1 | PF00139 | AT5G10530.1 | lectin protein kinase, putative |
| gma-miR408d | Glyma02g26480.1 | PF02365 | AT1G01720.1 | ATAF1; transcription activator/ transcription factor |
| gma-miR156s | Glyma02g30670.1 | PF03110 | AT2G42200.1 | SPL9 (SQUAMOSA PROMOTER BINDING PROTEIN-LIKE 9); transcription factor |
| gma-miR167a | Glyma02g40650.1 | PF02362,PF06507 ,PF02309 | AT5G37020.1 | ARF8 (AUXIN RESPONSE FACTOR 8); transcription factor |
| gma-miR167a | Glyma02g40650.2 | PF02362,PF06507 | | |
| gma-miR169n-5p | Glyma02g47380.3 | PF02045 | | |
| gma-miR396c | Glyma03g02500.1 | PF08880,PF08879 ,PF11722 | AT2G45480.1 | AtGRF9 (GROWTH-REGULATING FACTOR 9); transcription activator |
| gma-miR171k-3p | Glyma03g03760.1 | PF03514 | AT4G00150.1 | scarecrow-like transcription factor 6 (SCL6) |
| gma-miR171u | Glyma03g03760.1 | PF03514 | AT4G00150.1 | scarecrow-like transcription factor 6 (SCL6) |
| gma-miR4406 | Glyma03g17300.1 | PF07250,PF09118 | AT3G53950.1 | glyoxal oxidase-related |
| gma-miR408d | Glyma03g26060.1 | PF02298 | AT3G60280.1 | UCC3 (UCLACYANIN 3); copper ion binding / electron carrier |
| gma-miR2111d | Glyma03g26740.1 | PF05757 | AT4G05180.1 | PSBQ-2; calcium ion binding |
| gma-miR1507a | Glyma03g29370.1 | PF00931,PF00560 | AT3G14470.1 | disease resistance protein (NBS-LRR class), putative |
| gma-miR169v | Glyma03g36140.1 | PF02045 | AT5G06510.1 | NF-YA10 (NUCLEAR FACTOR Y, SUBUNIT A10); transcription factor |
| gma-miR169t | Glyma03g36140.2 | PF02045 | | |
| gma-miR398c | Glyma03g36190.1 | | AT3G11760.1 | unknown protein |
| gma-miR396e | Glyma03g36370.1 | PF04576 | AT5G06560.1 | unknown protein |
| gma-miR393k | Glyma03g36770.1 | PF00560,PF07723 | AT3G62980.1 | TIR1 (TRANSPORT INHIBITOR RESPONSE 1); auxin binding / protein binding / ubiquitin-protein ligase |

| | | | | | | |
|---|---|---|---|---|---|---|
| gma-miR396e | Glyma03g37700.1 | PF00515,PF07719,PF00226 | | | AT2G47440.1 | DNAJ heat shock N-terminal domain-containing protein |
| gma-miR398c | Glyma03g40280.2 | PF00080 | | | | |
| gma-miR398c | Glyma03g40280.3 | PF00080 | 1.15.1.1 | K04565 | | |
| gma-miR4416a | Glyma03g40640.1 | PF06026 | 5.3.1.6 | K01807 | AT3G04790.1 | ribose 5-phosphate isomerase-related |
| gma-miR1516d | Glyma03g40640.1 | PF06026 | 5.3.1.6 | K01807 | AT3G04790.1 | ribose 5-phosphate isomerase-related |
| gma-miR408d | Glyma03g41560.1 | PF01176 | | K03236 | AT2G04520.1 | eukaryotic translation initiation factor 1A, putative / eIF-1A, putative / eIF-4C, putative |
| gma-miR1507b | Glyma04g29220.2 | PF00931,PF00560 | | | | |
| gma-miR482c-5p | Glyma04g32400.1 | | | | AT1G74220.1 | unknown protein |
| gma-miR1510b-3p | Glyma04g39740.1 | PF01582 | | | AT5G36930.1 | disease resistance protein (TIR-NBS-LRR class), putative |
| gma-miR408d | Glyma04g42120.1 | PF02298 | | | AT2G02850.1 | ARPN (PLANTACYANIN); copper ion binding / electron carrier |
| gma-miR4996 | Glyma05g01480.1 | PF01535,PF01713 | | | AT1G74750.1 | pentatricopeptide (PPR) repeat-containing protein |
| gma-miR398c | Glyma05g04170.1 | PF00403,PF00080 | | | AT1G12520.1 | ATCCS (COPPER CHAPERONE FOR SOD1); superoxide dismutase/ superoxide dismutase copper chaperone |
| gma-miR5374-5p | Glyma05g09440.1 | PF00931,PF00560 | | | AT5G66900.1 | disease resistance protein (CC-NBS-LRR class), putative |
| gma-miR172e | Glyma05g18170.1 | PF00847 | | | AT4G36920.1 | AP2 (APETALA 2); transcription factor |
| gma-miR4416a | Glyma05g25190.2 | PF03998 | | | | |
| gma-miR167f | Glyma05g27580.1 | PF02362,PF06507,PF02309 | | | AT1G30330.1 | ARF6 (AUXIN RESPONSE FACTOR 6); transcription factor |
| gma-miR408d | Glyma05g30380.1 | PF02298 | | | AT2G02850.1 | ARPN (PLANTACYANIN); copper ion binding / electron carrier |
| gma-miR398c | Glyma05g35830.1 | PF00481 | | | AT4G26080.1 | ABI1 (ABA INSENSITIVE 1); calcium ion binding / protein serine/threonine phosphatase |
| gma-miR4377 | Glyma05g38130.1 | PF00314 | | | AT4G11650.1 | ATOSM34 (osmotin 34) |

| | | | | | | |
|---|---|---|---|---|---|---|
| gma-miR5670a | Glyma06g01470.1 | PF00076,PF07172 | | | AT2G21660.1 | CCR2 (COLD, CIRCADIAN RHYTHM, AND RNA BINDING 2); RNA binding / double-stranded DNA binding / single-stranded DNA binding |
| gma-miR408d | Glyma06g05890.1 | PF00403,PF00122 ,PF00702 | | | AT5G21930.1 | PAA2 (P-TYPE ATPASE OF ARABIDOPSIS 2); ATPase, coupled to transmembrane movement of ions, phosphorylative mechanism / copper ion transmembrane transporter |
| gma-miR408d | Glyma06g10500.1 | PF02298 | | | AT1G72230.1 | plastocyanin-like domain-containing protein |
| gma-miR408d | Glyma06g12680.1 | PF02298 | | | AT2G02850.1 | ARPN (PLANTACYANIN); copper ion binding / electron carrier |
| gma-miR396c | Glyma06g13960.1 | PF08880,PF08879 | | | AT5G53660.1 | AtGRF7 (GROWTH-REGULATING FACTOR 7); transcription activator |
| gma-miR396i-5p | Glyma06g13960.2 | PF08880,PF08879 | | | | |
| gma-miR482d-3p | Glyma06g17560.1 | PF00931,PF00560 | | | AT3G14470.1 | disease resistance protein (NBS-LRR class), putative |
| gma-miR168b | Glyma06g17680.1 | PF00403 | | | AT5G50740.1 | metal ion binding |
| gma-miR530e | Glyma06g17780.1 | PF00125,PF00808 | | | AT3G48590.1 | NF-YC1 (NUCLEAR FACTOR Y, SUBUNIT C1); DNA binding / transcription activator/ transcription factor |
| gma-miR398c | Glyma06g19680.1 | PF00412 | | | AT1G10200.1 | WLIM1; transcription factor/ zinc ion binding |
| gma-miR1511 | Glyma06g20600.1 | | | | AT1G74530.1 | unknown protein |
| gma-miR1507a | Glyma06g39720.1 | PF00931,PF00560 | | | AT3G14470.1 | disease resistance protein (NBS-LRR class), putative |
| gma-miR166t | Glyma07g01940.1 | PF00046,PF01852 ,PF08670 | | K09338 | AT1G52150.1 | ATHB-15; DNA binding / transcription factor |
| gma-miR166j-3p | Glyma07g01940.2 | PF01852 | | | | |
| gma-miR398c | Glyma07g03680.4 | PF01215,PF10276 | 1.9.3.1 | K02265 | | |
| gma-miR4413b | Glyma07g04420.2 | PF06747 | | | | |
| gma-miR171k-3p | Glyma07g09570.1 | | | | AT1G67910.1 | unknown protein |

| | | | | | | |
|---|---|---|---|---|---|---|
| gma-miR5674b | Glyma07g11500.1 | PF01535 | | | AT1G12700.1 | helicase domain-containing protein / pentatricopeptide (PPR) repeat-containing protein |
| gma-miR408d | Glyma07g13840.1 | PF02298 | | | AT3G60280.1 | UCC3 (UCLACYANIN 3); copper ion binding / electron carrier |
| gma-miR408d | Glyma07g18500.1 | PF00557 | | | AT3G51800.1 | ATG2; aminopeptidase/ metalloexopeptidase |
| gma-miR4375 | Glyma07g39640.1 | PF02985,PF00514 | | | AT4G16490.1 | binding |
| gma-miR167f | Glyma08g10550.1 | PF02362,PF06507,PF02309 | | | AT5G37020.1 | ARF8 (AUXIN RESPONSE FACTOR 8); transcription factor |
| gma-miR393k | Glyma08g12810.1 | PF07653,PF00018 | | | AT4G18060.1 | clathrin binding |
| gma-miR4409 | Glyma08g13300.2 | PF00996 | | | | |
| gma-miR408d | Glyma08g13510.1 | PF02298 | | | AT2G02850.1 | ARPN (PLANTACYANIN); copper ion binding / electron carrier |
| gma-miR1520p | Glyma08g15650.1 | PF04043,PF01095 | | | AT5G09760.1 | pectinesterase family protein |
| gma-miR164d | Glyma08g18470.1 | PF02365 | | | AT3G12977.1 | DNA binding |
| gma-miR398c | Glyma08g22410.1 | PF01215,PF10276 | 1.9.3.1 | K02265 | AT1G80230.1 | cytochrome c oxidase family protein |
| gma-miR398c | Glyma08g22410.2 | | | | | |
| gma-miR166j-3p | Glyma09g02750.1 | PF00046,PF00170,PF01852,PF08670 | | K09338 | AT2G34710.1 | PHB (PHABULOSA); DNA binding / transcription factor |
| gma-miR168b | Glyma09g29720.1 | PF08699,PF02170,PF02171 | | | AT1G48410.1 | AGO1 (ARGONAUTE 1); endoribonuclease/ miRNA binding / protein binding / siRNA binding |
| gma-miR5674b | Glyma09g30160.1 | PF01535 | | | AT1G12700.1 | helicase domain-containing protein / pentatricopeptide (PPR) repeat-containing protein |
| gma-miR5674b | Glyma09g30580.1 | PF01535 | | | AT1G12700.1 | helicase domain-containing protein / pentatricopeptide (PPR) repeat-containing protein |
| gma-miR5674b | Glyma09g30720.1 | PF01535 | | | AT1G62910.1 | pentatricopeptide (PPR) repeat-containing protein |
| gma-miR5674b | Glyma09g30940.1 | PF01535 | | | AT1G12700.1 | helicase domain-containing protein / pentatricopeptide (PPR) repeat-containing protein |
| gma-miR167j | Glyma09g34020.1 | | | | AT1G32240.1 | KAN2 (KANADI 2); DNA binding / transcription factor |

| | | | | | | |
|---|---|---|---|---|---|---|
| gma-miR4356 | Glyma09g34390.1 | PF00270,PF00271 | | | AT1G31970.1 | STRS1 (STRESS RESPONSE SUPPRESSOR 1); ATP binding / ATP-dependent helicase/ helicase/ nucleic acid binding |
| gma-miR5036 | Glyma09g38610.1 | PF02605 | | K02699 | AT4G12800.1 | PSAL (photosystem I subunit L) |
| gma-miR1508c | Glyma09g39260.1 | PF01535 | | | AT1G12700.1 | helicase domain-containing protein / pentatricopeptide (PPR) repeat-containing protein |
| gma-miR408d | Glyma10g00440.1 | PF08392,PF02797 | | | AT2G26640.1 | KCS11 (3-KETOACYL-COA SYNTHASE 11); acyltransferase/ catalytic/ transferase, transferring acyl groups other than amino-acyl groups |
| gma-miR393k | Glyma10g02630.1 | | | | AT3G62980.1 | TIR1 (TRANSPORT INHIBITOR RESPONSE 1); auxin binding / protein binding / ubiquitin-protein ligase |
| gma-miR5675 | Glyma10g03610.1 | | | | AT3G22942.1 | AGG2 (G-PROTEIN GAMMA SUBUNIT 2) |
| gma-miR396e | Glyma10g07790.1 | PF08880,PF08879 | | | AT2G36400.1 | AtGRF3 (GROWTH-REGULATING FACTOR 3); transcription activator |
| gma-miR169t | Glyma10g10240.1 | PF02045 | | | AT5G06510.1 | NF-YA10 (NUCLEAR FACTOR Y, SUBUNIT A10); transcription factor |
| gma-miR1520p | Glyma10g23630.2 | PF02893 | | | | |
| gma-miR4389 | Glyma10g34760.1 | PF00847,PF02362 | | | AT1G25560.1 | TEM1 (TEMPRANILLO 1); transcription factor |
| gma-miR160f | Glyma10g35480.1 | PF06507 | | | AT4G30080.1 | ARF16 (AUXIN RESPONSE FACTOR 16); miRNA binding / transcription factor |
| gma-miR395g | Glyma10g38760.1 | PF01747 | 2.7.7.4 | K00958 | AT3G22890.1 | APS1 (ATP SULFURYLASE 1); sulfate adenylyltransferase (ATP) |
| gma-miR4377 | Glyma10g42660.1 | PF12171,PF00096,PF08071 | | | AT2G02070.1 | AtIDD5 (Arabidopsis thaliana Indeterminate(ID)-Domain 5); nucleic acid binding / transcription factor/ zinc ion binding |
| gma-miR396e | Glyma11g01060.1 | PF08880,PF08879 | | | AT3G13960.1 | AtGRF5 (GROWTH-REGULATING FACTOR 5); transcription activator |

| | | | | | | |
|---|---|---|---|---|---|---|
| gma-miR319i | Glyma11g04210.1 | PF01373 | | | AT4G17090.1 | CT-BMY (CHLOROPLAST BETA-AMYLASE); beta-amylase |
| gma-miR172l | Glyma11g05720.1 | PF00847 | | | AT4G36920.1 | AP2 (APETALA 2); transcription factor |
| gma-miR172j | Glyma11g06830.2 | PF00179 | 6.3.2.19 | K10579 | | |
| gma-miR171k-3p | Glyma11g11760.2 | PF00137 | | | | |
| gma-miR396e | Glyma11g11820.1 | PF08880,PF08879 | | | AT3G13960.1 | AtGRF5 (GROWTH-REGULATING FACTOR 5); transcription activator |
| gma-miR172h-3p | Glyma11g15650.3 | PF00847 | | | | |
| gma-miR171i-3p | Glyma11g17490.1 | PF03514 | | | AT4G00150.1 | scarecrow-like transcription factor 6 (SCL6) |
| gma-miR160f | Glyma11g20490.1 | PF02362,PF06507 | | | AT2G28350.1 | ARF10 (AUXIN RESPONSE FACTOR 10); miRNA binding / transcription factor |
| gma-miR166t | Glyma11g20520.1 | PF00046,PF00170,PF01852,PF08670 | | K09338 | AT5G60690.1 | REV (REVOLUTA); DNA binding / lipid binding / transcription factor |
| gma-miR167a | Glyma11g31940.1 | PF02362,PF06507,PF02309 | | | AT5G37020.1 | ARF8 (AUXIN RESPONSE FACTOR 8); transcription factor |
| gma-miR4386 | Glyma11g35050.1 | PF07231,PF07014 | | | AT2G40000.1 | HSPRO2 (ARABIDOPSIS ORTHOLOG OF SUGAR BEET HS1 PRO-1 2) |
| gma-miR5037a | Glyma11g36090.1 | PF01269 | | | AT4G25630.1 | FIB2 (FIBRILLARIN 2); snoRNA binding |
| gma-miR398c | Glyma11g36670.1 | PF05694 | | | AT4G14030.1 | SBP1 (selenium-binding protein 1); selenium binding |
| gma-miR156s | Glyma11g36980.1 | PF03110 | | | AT5G43270.1 | SPL2 (SQUAMOSA PROMOTER BINDING PROTEIN-LIKE 2); DNA binding / transcription factor |
| gma-miR156f | Glyma11g36980.1 | PF03110 | | | AT5G43270.1 | SPL2 (SQUAMOSA PROMOTER BINDING PROTEIN-LIKE 2); DNA binding / transcription factor |

| | | | | | | |
|---|---|---|---|---|---|---|
| gma-miR408b-5p | Glyma12g00330.1 | PF00628,PF00097,PF02182 | | | AT1G57820.1 | VIM1 (VARIANT IN METHYLATION 1); DNA binding / chromatin binding / double-stranded methylated DNA binding / histone binding / methyl-CpG binding / methyl-CpNpG binding / methyl-CpNpN binding / ubiquitin-protein ligase |
| gma-miR396e | Glyma12g01730.1 | PF08880,PF08879 | | | AT3G13960.1 | AtGRF5 (GROWTH-REGULATING FACTOR 5); transcription activator |
| gma-miR169t | Glyma12g02790.1 | PF00160 | 5.2.1.8 | K01802 | AT2G16600.1 | ROC3; peptidyl-prolyl cis-trans isomerase |
| gma-miR164d | Glyma12g06990.1 | PF01370 | 4.2.1.46 | K01710 | AT2G28760.1 | NAD-dependent epimerase/dehydratase family protein |
| gma-miR160f | Glyma12g08110.1 | PF02362,PF06507 | | | AT2G28350.1 | ARF10 (AUXIN RESPONSE FACTOR 10); miRNA binding / transcription factor |
| gma-miR164k | Glyma12g26190.1 | PF02365 | | | AT5G53950.1 | CUC2 (CUP-SHAPED COTYLEDON 2); transcription factor |
| gma-miR156s | Glyma12g27330.1 | PF03110 | | | AT3G60030.1 | SPL12 (squamosa promoter-binding protein-like 12); transcription factor |
| gma-miR1510b-3p | Glyma12g27800.1 | PF01582 | | | AT5G17680.1 | disease resistance protein (TIR-NBS-LRR class), putative |
| gma-miR160f | Glyma12g29720.1 | PF02362,PF06507 | | | AT2G28350.1 | ARF10 (AUXIN RESPONSE FACTOR 10); miRNA binding / transcription factor |
| gma-miR159e-5p | Glyma12g34770.1 | PF00504 | | K08914 | AT5G54270.1 | LHCB3 (LIGHT-HARVESTING CHLOROPHYLL B-BINDING PROTEIN 3); structural molecule |
| gma-miR319d | Glyma12g35720.1 | PF03634 | | | AT3G15030.1 | TCP4 (TCP family transcription factor 4); transcription factor |
| gma-miR4378a | Glyma13g00520.2 | PF09425,PF06203 | | | | |
| gma-miR160f | Glyma13g02410.1 | PF02362,PF06507 | | | AT1G77850.1 | ARF17 (AUXIN RESPONSE FACTOR 17); transcription factor |
| gma-miR171b-3p | Glyma13g02840.1 | PF03514 | | | AT4G08250.1 | scarecrow transcription factor family protein |

| | | | | | | |
|---|---|---|---|---|---|---|
| gma-miR4415b-3p | Glyma13g03650.1 | PF07732,PF00394,PF07731 | | | AT5G21105.1 | L-ascorbate oxidase/ copper ion binding / oxidoreductase |
| gma-miR408d | Glyma13g17840.2 | PF00628 | | | | |
| gma-miR4996 | Glyma13g29160.1 | PF03634 | | | AT4G18390.1 | TCP family transcription factor, putative |
| gma-miR167f | Glyma13g29320.1 | PF02362,PF06507,PF02309 | | | AT1G30330.1 | ARF6 (AUXIN RESPONSE FACTOR 6); transcription factor |
| gma-miR319d | Glyma13g34690.1 | PF03634 | | | AT3G15030.1 | TCP4 (TCP family transcription factor 4); transcription factor |
| gma-miR159e-3p | Glyma13g34710.1 | | | | | |
| gma-miR160f | Glyma13g40030.1 | PF02362,PF06507 | | | AT2G28350.1 | ARF10 (AUXIN RESPONSE FACTOR 10); miRNA binding / transcription factor |
| gma-miR172h-3p | Glyma13g40470.1 | PF00847 | | | AT2G28550.1 | RAP2.7 (RELATED TO AP2.7); DNA binding / transcription factor |
| gma-miR172h-3p | Glyma13g40470.2 | | | | | |
| gma-miR172h-3p | Glyma13g40470.3 | | | | | |
| gma-miR169u | Glyma14g01360.1 | PF02045 | | | AT3G20910.1 | NF-YA9 (NUCLEAR FACTOR Y, SUBUNIT A9); specific transcriptional repressor/ transcription factor |
| gma-miR167f | Glyma14g03650.2 | PF02362,PF06507,PF02309 | | | | |
| gma-miR4415b-3p | Glyma14g04530.1 | PF07732,PF00394,PF07731 | | | AT5G21105.1 | L-ascorbate oxidase/ copper ion binding / oxidoreductase |
| gma-miR1524 | Glyma14g09670.1 | PF05922,PF00082,PF02225 | | | AT5G67360.1 | ARA12; serine-type endopeptidase |
| gma-miR166i-5p | Glyma14g10020.1 | PF00170,PF07716 | | | AT3G49760.1 | AtbZIP5 (Arabidopsis thaliana basic leucine-zipper 5); DNA binding / transcription factor |
| gma-miR396i-5p | Glyma14g10090.1 | PF08880,PF08879 | | | AT2G22840.1 | AtGRF1 (GROWTH-REGULATING FACTOR 1); transcription activator |
| gma-miR396c | Glyma14g10100.1 | PF08880,PF08879 | | | AT2G22840.1 | AtGRF1 (GROWTH-REGULATING FACTOR 1); transcription activator |
| gma-miR167a | Glyma14g38940.1 | PF02362,PF06507,PF02309 | | | AT5G37020.1 | ARF8 (AUXIN RESPONSE FACTOR 8); transcription factor |
| gma-miR398c | Glyma14g39910.2 | PF02897,PF00326 | 3.4.21.26 | K01322 | | |

| | | | | | |
|---|---|---|---|---|---|
| gma-miR167j | Glyma15g00770.2 | PF07576,PF00097,PF02148 | | | |
| gma-miR172h-3p | Glyma15g04930.1 | PF00847 | | AT2G28550.1 | RAP2.7 (RELATED TO AP2.7); DNA binding / transcription factor |
| gma-miR4416a | Glyma15g06650.2 | PF01641 | | | |
| gma-miR167f | Glyma15g09750.1 | PF02362,PF06507,PF02309 | | AT1G30330.1 | ARF6 (AUXIN RESPONSE FACTOR 6); transcription factor |
| gma-miR164k | Glyma15g40510.1 | PF02365 | | AT3G12977.1 | DNA binding |
| gma-miR393k | Glyma16g05500.1 | PF00560 | | AT3G26810.1 | AFB2 (AUXIN SIGNALING F-BOX 2); auxin binding / ubiquitin-protein ligase |
| gma-miR156m | Glyma16g05900.1 | | | | |
| gma-miR4996 | Glyma16g06100.1 | PF10539 | | AT3G27090.1 | unknown protein |
| gma-miR168b | Glyma16g34300.1 | PF08699,PF02170,PF02171 | | AT1G48410.1 | AGO1 (ARGONAUTE 1); endoribonuclease/ miRNA binding / protein binding / siRNA binding |
| gma-miR408d | Glyma17g04670.1 | | | AT1G09520.1 | protein binding / zinc ion binding |
| gma-miR4403 | Glyma17g05840.1 | PF07876 | | AT2G31670.1 | unknown protein |
| gma-miR156g | Glyma17g08840.1 | PF03110 | | AT1G69170.1 | squamosa promoter-binding protein-like 6 (SPL6) |
| gma-miR156y | Glyma17g08840.1 | PF03110 | | AT1G69170.1 | squamosa promoter-binding protein-like 6 (SPL6) |
| gma-miR172e | Glyma17g18640.2 | PF00847 | K09284 | | |
| gma-miR396i-5p | Glyma17g35090.1 | PF08880,PF08879 | | AT2G22840.1 | AtGRF1 (GROWTH-REGULATING FACTOR 1); transcription activator |
| gma-miR396i-5p | Glyma17g35100.1 | PF08880,PF08879 | | AT2G22840.1 | AtGRF1 (GROWTH-REGULATING FACTOR 1); transcription activator |
| gma-miR156s | Glyma18g00890.1 | | | | |
| gma-miR395g | Glyma18g02240.1 | PF00916,PF01740 | | AT5G10180.1 | AST68; sulfate transmembrane transporter |
| gma-miR167a | Glyma18g05330.1 | PF02362,PF06507,PF02309 | | AT5G37020.1 | ARF8 (AUXIN RESPONSE FACTOR 8); transcription factor |
| gma-miR1520c | Glyma18g11400.1 | | | AT5G24930.1 | zinc finger (B-box type) family protein |
| gma-miR5042-5p | Glyma18g13650.1 | PF00560 | | AT5G45520.1 | unknown protein |

| | | | | | | |
|---|---|---|---|---|---|---|
| gma-miR156s | Glyma18g36960.1 | PF03110 | | | AT2G42200.1 | SPL9 (SQUAMOSA PROMOTER BINDING PROTEIN-LIKE 9); transcription factor |
| gma-miR168b | Glyma19g01700.1 | PF01985 | | | AT3G01370.1 | ATCFM2 (CRM FAMILY MEMBER 2); RNA binding |
| gma-miR1510b-3p | Glyma19g07700.1 | PF00931,PF05729,PF00560 | | | AT5G36930.1 | disease resistance protein (TIR-NBS-LRR class), putative |
| gma-miR156y | Glyma19g26390.1 | PF03110 | | | AT5G50570.1 | squamosa promoter-binding protein, putative |
| gma-miR156m | Glyma19g26390.1 | PF03110 | | | AT5G50570.1 | squamosa promoter-binding protein, putative |
| gma-miR393k | Glyma19g27280.1 | PF00560 | | | AT3G26810.1 | AFB2 (AUXIN SIGNALING F-BOX 2); auxin binding / ubiquitin-protein ligase |
| gma-miR169l-3p | Glyma19g32940.1 | PF00487 | 1.14.19.- | K10256 | AT3G12120.1 | FAD2 (FATTY ACID DESATURASE 2); delta12-fatty acid dehydrogenase/ omega-6 fatty acid desaturase |
| gma-miR396e | Glyma19g33530.1 | PF03909 | | | AT1G03350.1 | BSD domain-containing protein |
| gma-miR1532 | Glyma19g34860.1 | PF01380,PF00571 | | | AT3G54690.1 | sugar isomerase (SIS) domain-containing protein / CBS domain-containing protein |
| gma-miR172l | Glyma19g36200.1 | PF00847 | | | AT4G36920.1 | AP2 (APETALA 2); transcription factor |
| gma-miR160f | Glyma19g36570.1 | PF06507 | | | AT4G30080.1 | ARF16 (AUXIN RESPONSE FACTOR 16); miRNA binding / transcription factor |
| gma-miR169v | Glyma19g38800.1 | PF02045 | | | AT5G06510.1 | NF-YA10 (NUCLEAR FACTOR Y, SUBUNIT A10); transcription factor |
| gma-miR169p | Glyma19g38800.1 | PF02045 | | | AT5G06510.1 | NF-YA10 (NUCLEAR FACTOR Y, SUBUNIT A10); transcription factor |
| gma-miR319c | Glyma19g42270.1 | PF00887,PF07646,PF01344 | | | AT3G05420.1 | ACBP4 (ACYL-COA BINDING PROTEIN 4); acyl-CoA binding |
| gma-miR398c | Glyma19g42890.3 | PF00080 | | | | |
| gma-miR4415b-3p | Glyma20g12150.1 | PF07732,PF00394,PF07731 | | | AT5G21105.1 | L-ascorbate oxidase/ copper ion binding / oxidoreductase |
| gma-miR398c | Glyma20g26270.1 | PF07731 | | | AT1G71040.1 | LPR2 (Low Phosphate Root2); copper ion binding / oxidoreductase |

| | | | | | | |
|---|---|---|---|---|---|---|
| gma-miR4380b | Glyma20g29280.1 | | | | AT5G51840.1 | unknown protein |
| gma-miR160f | Glyma20g32040.1 | PF02362,PF06507 | | | AT4G30080.1 | ARF16 (AUXIN RESPONSE FACTOR 16); miRNA binding / transcription factor |
| gma-miR1523a | Glyma01g03720.3 | PF00484 | 4.2.1.1 | K01673 | | |
| gma-miR5673 | Glyma01g28810.1 | PF00504 | | K08915 | AT5G01530.1 | chlorophyll A-B binding protein CP29 (LHCB4) |
| gma-miR1516c | Glyma01g32750.1 | PF00234 | | | AT2G38540.1 | LP1; calmodulin binding |
| gma-miR171k-3p | Glyma01g38360.1 | PF03514 | | | AT4G36710.1 | transcription factor |
| gma-miR396e | Glyma02g11630.1 | | | | AT4G34135.1 | UGT73B2 (UDP-GLUCOSYLTRANSFERASE 73B2); UDP-glucosyltransferase/ UDP-glycosyltransferase/ flavonol 3-O-glucosyltransferase/ quercetin 7-O-glucosyltransferase |
| gma-miR393k | Glyma02g17170.1 | PF00646 | | | AT3G62980.1 | TIR1 (TRANSPORT INHIBITOR RESPONSE 1); auxin binding / protein binding / ubiquitin-protein ligase |
| gma-miR156f | Glyma02g30670.1 | PF03110 | | | AT2G42200.1 | SPL9 (SQUAMOSA PROMOTER BINDING PROTEIN-LIKE 9); transcription factor |
| gma-miR169t | Glyma02g35190.1 | PF02045 | | | AT5G06510.1 | NF-YA10 (NUCLEAR FACTOR Y, SUBUNIT A10); transcription factor |
| gma-miR169l-5p | Glyma02g47380.1 | PF02045 | | | AT3G20910.1 | NF-YA9 (NUCLEAR FACTOR Y, SUBUNIT A9); specific transcriptional repressor/ transcription factor |
| gma-miR396i-5p | Glyma03g02500.1 | PF08880,PF08879 ,PF11722 | | | AT2G45480.1 | AtGRF9 (GROWTH-REGULATING FACTOR 9); transcription activator |
| gma-miR171a | Glyma03g03760.1 | PF03514 | | | AT4G00150.1 | scarecrow-like transcription factor 6 (SCL6) |
| gma-miR408d | Glyma03g26060.2 | PF02298 | | | | |
| gma-miR396c | Glyma03g30630.1 | PF03909 | | | AT1G03350.1 | BSD domain-containing protein |
| gma-miR169v | Glyma03g36140.2 | PF02045 | | | | |

| | | | | | | |
|---|---|---|---|---|---|---|
| gma-miR162c | Glyma03g42290.2 | PF04851,PF00270,PF00271,PF03368,PF02170,PF00636,PF00035 | | K01165 | | |
| gma-miR1507b | Glyma04g29220.1 | PF00931,PF00560 | | | AT3G14470.1 | disease resistance protein (NBS-LRR class), putative |
| gma-miR396c | Glyma04g40880.1 | PF08879 | | | AT5G53660.1 | AtGRF7 (GROWTH-REGULATING FACTOR 7); transcription activator |
| gma-miR160f | Glyma04g43350.1 | PF02362,PF06507 | | | AT1G77850.1 | ARF17 (AUXIN RESPONSE FACTOR 17); transcription factor |
| gma-miR156y | Glyma05g00200.1 | PF03110 | | | AT1G69170.1 | squamosa promoter-binding protein-like 6 (SPL6) |
| gma-miR1517 | Glyma05g27040.1 | PF10213 | | | AT3G18240.1 | unknown protein |
| gma-miR4362 | Glyma05g34900.1 | PF00764 | 6.3.4.5 | K01940 | AT4G24830.1 | arginosuccinate synthase family |
| gma-miR396k-3p | Glyma06g05810.1 | PF06596 | | | AT2G06520.1 | PSBX (photosystem II subunit X) |
| gma-miR1508b | Glyma06g07400.1 | PF00071,PF08477 | | K07936 | AT5G55190.1 | RAN3 (RAN GTPASE 3); GTP binding / GTPase/ protein binding |
| gma-miR396g | Glyma06g13600.2 | PF00481 | | | | |
| gma-miR171j-5p | Glyma06g48360.4 | PF02219 | 1.5.1.20 | K00297 | | |
| gma-miR1515b | Glyma07g01940.1 | PF00046,PF01852,PF08670 | | K09338 | AT1G52150.1 | ATHB-15; DNA binding / transcription factor |
| gma-miR166j-3p | Glyma07g01940.3 | PF00046,PF01852 | | | | |
| gma-miR398c | Glyma07g03680.1 | PF01215,PF10276 | 1.9.3.1 | K02265 | AT1G80230.1 | cytochrome c oxidase family protein |
| gma-miR169p | Glyma07g04050.3 | PF02045 | | | | |
| gma-miR169n-5p | Glyma07g08810.1 | PF01553,PF00036 | | | AT2G45670.1 | calcineurin B subunit-related |
| gma-miR4403 | Glyma07g16230.1 | | | | | |
| gma-miR166j-5p | Glyma07g36770.1 | PF00378 | | | AT4G16210.1 | ECHIA (ENOYL-COA HYDRATASE/ISOMERASE A); catalytic |
| gma-miR5374-5p | Glyma07g40390.2 | PF06093 | | | | |
| gma-miR4376a-3p | Glyma08g01100.2 | PF02362,PF06507,PF02309 | | | | |
| gma-miR394g | Glyma08g11030.2 | PF00646 | | | | |
| gma-miR4409 | Glyma08g13300.3 | PF00996 | | | | |
| gma-miR164k | Glyma08g18470.1 | PF02365 | | | AT3G12977.1 | DNA binding |

| | | | | | | |
|---|---|---|---|---|---|---|
| gma-miR4996 | Glyma08g23930.2 | PF11976,PF00240,PF00627,PF09280 | | | | |
| gma-miR1508c | Glyma09g07290.1 | PF01535 | | | AT1G12700.1 | helicase domain-containing protein / pentatricopeptide (PPR) repeat-containing protein |
| gma-miR169n-5p | Glyma09g07960.2 | PF02045 | | | | |
| gma-miR4376a-3p | Glyma09g38490.1 | | | | AT1G65230.1 | unknown protein |
| gma-miR4401a | Glyma10g43560.2 | | | | | |
| gma-miR5675 | Glyma11g09980.1 | PF04055,PF06968 | | | AT2G43360.1 | BIO2 (BIOTIN AUXOTROPH 2); biotin synthase |
| gma-miR172e | Glyma11g15650.1 | PF00847 | | | AT2G28550.1 | RAP2.7 (RELATED TO AP2.7); DNA binding / transcription factor |
| gma-miR1507b | Glyma11g33160.1 | PF01704 | 2.7.7.9 | K00963 | AT3G03250.1 | UGP (UDP-glucose pyrophosphorylase); UTP:glucose-1-phosphate uridylyltransferase/ nucleotidyltransferase |
| gma-miR156y | Glyma11g36980.1 | PF03110 | | | AT5G43270.1 | SPL2 (SQUAMOSA PROMOTER BINDING PROTEIN-LIKE 2); DNA binding / transcription factor |
| gma-miR396e | Glyma12g01730.2 | PF08880,PF08879 | | | | |
| gma-miR1517 | Glyma12g02790.1 | PF00160 | 5.2.1.8 | K01802 | AT2G16600.1 | ROC3; peptidyl-prolyl cis-trans isomerase |
| gma-miR172h-3p | Glyma12g07800.1 | PF00847 | | K09284 | AT2G28550.1 | RAP2.7 (RELATED TO AP2.7); DNA binding / transcription factor |
| gma-miR172h-3p | Glyma12g07800.2 | PF00847 | | K09284 | | |
| gma-miR166j-3p | Glyma12g08080.1 | PF00046,PF00170,PF01852,PF08670 | | K09338 | AT5G60690.1 | REV (REVOLUTA); DNA binding / lipid binding / transcription factor |
| gma-miR319d | Glyma12g20160.1 | PF03634 | | | AT1G53230.1 | TCP3; transcription factor |
| gma-miR156y | Glyma12g27330.1 | PF03110 | | | AT3G60030.1 | SPL12 (squamosa promoter-binding protein-like 12); transcription factor |
| gma-miR408d | Glyma12g29270.2 | PF02891 | | | | |
| gma-miR171j-5p | Glyma12g30320.1 | | | | AT2G27980.1 | protein binding / zinc ion binding |

| | | | | | | |
|---|---|---|---|---|---|---|
| gma-miR2119 | Glyma12g32580.1 | PF08802,PF00355 | 1.10.99.1 | K02636 | AT4G03280.1 | PETC (PHOTOSYNTHETIC ELECTRON TRANSFER C); electron transporter, transferring electrons from cytochrome b6/f complex of photosystem II |
| gma-miR171k-3p | Glyma13g02840.1 | PF03514 | | | AT4G08250.1 | scarecrow transcription factor family protein |
| gma-miR1520h | Glyma13g06630.1 | PF08693,PF02439,PF00069,PF07714 | | | AT3G51550.1 | FER (FERONIA); kinase/ protein kinase |
| gma-miR4995 | Glyma13g15140.2 | PF08267,PF01717 | | | | |
| gma-miR169n-5p | Glyma13g16770.3 | PF02045 | | | | |
| gma-miR160f | Glyma13g20370.1 | PF02362,PF06507 | | | AT4G30080.1 | ARF16 (AUXIN RESPONSE FACTOR 16); miRNA binding / transcription factor |
| gma-miR167f | Glyma13g29320.2 | PF02362,PF06507 | | | | |
| gma-miR171j-5p | Glyma13g37700.1 | PF12609 | | | AT4G28240.1 | wound-responsive protein-related |
| gma-miR395g | Glyma13g37880.1 | PF08802,PF00355 | 1.10.99.1 | K02636 | AT4G03280.1 | PETC (PHOTOSYNTHETIC ELECTRON TRANSFER C); electron transporter, transferring electrons from cytochrome b6/f complex of photosystem II |
| gma-miR2118b-3p | Glyma13g37990.1 | PF00036 | | | AT4G27280.1 | calcium-binding EF hand family protein |
| gma-miR169n-5p | Glyma14g01130.1 | PF00504 | | K08913 | AT2G05100.1 | LHCB2.1; chlorophyll binding |
| gma-miR408b-5p | Glyma14g01130.1 | PF00504 | | K08913 | AT2G05100.1 | LHCB2.1; chlorophyll binding |
| gma-miR396c | Glyma14g10090.1 | PF08880,PF08879 | | | AT2G22840.1 | AtGRF1 (GROWTH-REGULATING FACTOR 1); transcription activator |
| gma-miR390d | Glyma14g36840.1 | PF02365 | | | AT4G35580.1 | NTL9 (NAC transcription factor-like 9); transcription factor |
| gma-miR398c | Glyma14g39910.3 | PF02897,PF00326 | 3.4.21.26 | K01322 | | |
| gma-miR394a-3p | Glyma15g10940.1 | PF00069,PF07714 | | | AT5G19010.1 | MPK16; MAP kinase |
| gma-miR390b-3p | Glyma15g12890.1 | PF00400 | | | AT3G13300.1 | VCS (VARICOSE); nucleotide binding / protein homodimerization |
| gma-miR166i-5p | Glyma15g42740.1 | PF01754,PF01428 | | | AT3G12630.1 | zinc finger (AN1-like) family protein |
| gma-miR2118b-3p | Glyma16g01050.1 | PF00069,PF07714 | | | AT1G72540.1 | protein kinase, putative |
| gma-miR156r | Glyma16g05900.1 | | | | | |

| | | | | | |
|---|---|---|---|---|---|
| gma-miR319h | Glyma16g27940.2 | PF03656 | | | |
| gma-miR397b-3p | Glyma16g29660.1 | PF00403 | | AT1G01490.1 | heavy-metal-associated domain-containing protein |
| gma-miR4393a | Glyma17g02410.1 | | | AT3G21570.1 | unknown protein |
| gma-miR396c | Glyma17g35090.1 | PF08880,PF08879 | | AT2G22840.1 | AtGRF1 (GROWTH-REGULATING FACTOR 1); transcription activator |
| gma-miR156y | Glyma18g00890.1 | | | | |
| gma-miR169l-5p | Glyma18g07890.1 | PF02045 | | AT5G12840.1 | NF-YA1 (NUCLEAR FACTOR Y, SUBUNIT A1); transcription factor |
| gma-miR319l | Glyma18g47710.1 | PF02605 | K02699 | AT4G12800.1 | PSAL (photosystem I subunit L) |
| gma-miR4361 | Glyma18g50940.1 | PF01323 | | AT5G38900.1 | DSBA oxidoreductase family protein |
| gma-miR169j-3p | Glyma18g52780.1 | PF00022 | K10355 | AT3G12110.1 | ACT11 (actin-11); structural constituent of cytoskeleton |
| gma-miR169h | Glyma19g02370.2 | PF01145 | | | |
| gma-miR1510b-3p | Glyma19g02670.1 | PF01582,PF00931,PF00560 | | AT5G36930.1 | disease resistance protein (TIR-NBS-LRR class), putative |
| gma-miR1507a | Glyma19g05600.1 | PF00931,PF00560 | | AT3G14470.1 | disease resistance protein (NBS-LRR class), putative |
| gma-miR171j-5p | Glyma19g30180.1 | PF00656 | | AT5G04200.1 | AtMC9 (metacaspase 9); cysteine-type peptidase |
| gma-miR156s | Glyma19g32800.1 | PF03110 | | AT2G42200.1 | SPL9 (SQUAMOSA PROMOTER BINDING PROTEIN-LIKE 9); transcription factor |
| gma-miR169t | Glyma19g38800.1 | PF02045 | | AT5G06510.1 | NF-YA10 (NUCLEAR FACTOR Y, SUBUNIT A10); transcription factor |
| gma-miR393k | Glyma19g39420.1 | PF00560,PF07723 | | AT3G62980.1 | TIR1 (TRANSPORT INHIBITOR RESPONSE 1); auxin binding / protein binding / ubiquitin-protein ligase |
| gma-miR5036 | Glyma20g25360.2 | PF00481 | | | |
| gma-miR4403 | Glyma20g26300.2 | PF04199 | | | |
| gma-miR169n-5p | Glyma20g27950.1 | PF11976,PF00240,PF07708 | K04551 | AT4G05320.1 | UBQ10 (POLYUBIQUITIN 10); protein binding |
| gma-miR171i-3p | Glyma20g38140.1 | PF00561 | | AT3G05600.1 | epoxide hydrolase, putative |
| gma-miR5037c | Glyma01g32750.1 | PF00234 | | AT2G38540.1 | LP1; calmodulin binding |
| gma-miR171u | Glyma01g33270.1 | PF03514 | | AT4G00150.1 | scarecrow-like transcription factor 6 (SCL6) |

| | | | | | | |
|---|---|---|---|---|---|---|
| gma-miR1520b | Glyma01g40620.1 | PF00010 | | | AT4G37850.1 | basic helix-loop-helix (bHLH) family protein |
| gma-miR1515b | Glyma02g10860.1 | PF02617 | | K06891 | AT1G68660.1 | unknown protein |
| gma-miR4997 | Glyma02g13330.1 | PF03214 | | | AT3G08900.1 | RGP3 (REVERSIBLY GLYCOSYLATED POLYPEPTIDE 3); transferase, transferring hexosyl groups |
| gma-miR169v | Glyma02g35190.1 | PF02045 | | | AT5G06510.1 | NF-YA10 (NUCLEAR FACTOR Y, SUBUNIT A10); transcription factor |
| gma-miR398c | Glyma02g46020.5 | PF01625 | 1.8.4.11 | K07304 | | |
| gma-miR5039 | Glyma03g04380.1 | | | | AT3G51600.1 | LTP5 (Lipid transfer protein 5); lipid transporter |
| gma-miR156s | Glyma03g29900.1 | PF03110 | | | AT2G42200.1 | SPL9 (SQUAMOSA PROMOTER BINDING PROTEIN-LIKE 9); transcription factor |
| gma-miR5037b | Glyma03g30880.1 | PF01423 | | K11087 | AT4G02840.1 | small nuclear ribonucleoprotein D1, putative / snRNP core protein D1, putative / Sm protein D1, putative |
| gma-miR169t | Glyma03g36140.1 | PF02045 | | | AT5G06510.1 | NF-YA10 (NUCLEAR FACTOR Y, SUBUNIT A10); transcription factor |
| gma-miR169d | Glyma03g36140.3 | PF02045 | | | | |
| gma-miR5044 | Glyma04g01040.1 | PF00628,PF02201,PF03126,PF02213,PF00642 | | | AT2G16485.1 | DNA binding / nucleic acid binding / protein binding / zinc ion binding |
| gma-miR172b-5p | Glyma04g33720.1 | PF00249 | | K09422 | AT3G47600.1 | ATMYB94 (MYB DOMAIN PROTEIN 94); DNA binding / transcription factor |
| gma-miR171b-3p | Glyma04g43090.1 | PF03514 | | | AT4G08250.1 | scarecrow transcription factor family protein |
| gma-miR164d | Glyma05g00930.1 | PF02365 | | | AT5G61430.1 | ANAC100 (ARABIDOPSIS NAC DOMAIN CONTAINING PROTEIN 100); transcription factor |
| gma-miR4409 | Glyma05g30140.2 | PF00996 | | | | |
| gma-miR156y | Glyma05g38180.1 | PF03110 | | | AT5G50670.1 | squamosa promoter-binding protein, putative |
| gma-miR169i-5p | Glyma06g04010.1 | PF00249 | | | AT2G23290.1 | AtMYB70 (myb domain protein 70); DNA binding / transcription factor |

| | | | | | | |
|---|---|---|---|---|---|---|
| gma-miR2107 | Glyma06g07140.1 | PF07651 | | | AT2G25430.1 | epsin N-terminal homology (ENTH) domain-containing protein |
| gma-miR166t | Glyma06g09100.1 | PF00046,PF01852 ,PF08670 | | K09338 | AT4G32880.1 | ATHB-8 (HOMEOBOX GENE 8); DNA binding / transcription factor |
| gma-miR396i-5p | Glyma06g13960.1 | PF08880,PF08879 | | | AT5G53660.1 | AtGRF7 (GROWTH-REGULATING FACTOR 7); transcription activator |
| gma-miR396c | Glyma06g13960.2 | PF08880,PF08879 | | | | |
| gma-miR5676 | Glyma06g18600.1 | PF00149 | | | AT4G24730.1 | calcineurin-like phosphoesterase family protein |
| gma-miR166j-5p | Glyma06g27660.2 | | | K06693 | | |
| gma-miR319d | Glyma06g34330.1 | PF03634 | | | AT1G53230.1 | TCP3; transcription factor |
| gma-miR2111d | Glyma07g01730.1 | PF03767 | | | AT4G25150.1 | acid phosphatase, putative |
| gma-miR166j-3p | Glyma07g01950.1 | PF00046,PF01852 ,PF08670 | | K09338 | AT1G52150.1 | ATHB-15; DNA binding / transcription factor |
| gma-miR166u | Glyma07g01950.1 | PF00046,PF01852 ,PF08670 | | K09338 | AT1G52150.1 | ATHB-15; DNA binding / transcription factor |
| gma-miR1512c | Glyma07g03890.1 | PF00400,PF04053 | | | AT1G52360.1 | coatomer protein complex, subunit beta 2 (beta prime), putative |
| gma-miR4992 | Glyma07g06160.1 | PF07719,PF00515 | | | AT1G01320.1 | tetratricopeptide repeat (TPR)-containing protein |
| gma-miR4997 | Glyma07g21670.1 | PF00271 | | | AT2G01440.1 | ATP binding / ATP-dependent DNA helicase/ ATP-dependent helicase/ helicase/ nucleic acid binding |
| gma-miR5672 | Glyma07g33870.1 | PF00571 | | | AT4G36910.1 | LEJ2 (LOSS OF THE TIMING OF ET AND JA BIOSYNTHESIS 2) |
| gma-miR1523b | Glyma08g05080.1 | PF00097 | | | AT1G68070.1 | zinc finger (C3HC4-type RING finger) family protein |
| gma-miR1509a | Glyma08g08430.1 | PF03145 | | | AT5G37930.1 | seven in absentia (SINA) family protein |
| gma-miR166t | Glyma08g21610.1 | PF00046,PF01852 ,PF08670 | | K09338 | AT1G52150.1 | ATHB-15; DNA binding / transcription factor |
| gma-miR5375 | Glyma09g04710.1 | PF01636,PF02770 | 1.3.99.3 | K00249 | AT3G06810.1 | IBR3 (IBA-RESPONSE 3); acyl-CoA dehydrogenase/ oxidoreductase |
| gma-miR169n-5p | Glyma09g07960.1 | PF02045 | | | AT1G72830.1 | NF-YA3 (NUCLEAR FACTOR Y, SUBUNIT A3); transcription factor |

| | | | | | | |
|---|---|---|---|---|---|---|
| gma-miR5674b | Glyma09g30680.1 | PF01535 | | | AT1G12700.1 | helicase domain-containing protein / pentatricopeptide (PPR) repeat-containing protein |
| gma-miR2108a | Glyma09g31740.1 | | | | | |
| gma-miR408d | Glyma09g35640.1 | PF01866 | | | AT5G62030.1 | diphthamide synthesis DPH2 family protein |
| gma-miR319l | Glyma09g39120.1 | | | | AT4G01290.1 | unknown protein |
| gma-miR168b | Glyma10g00790.1 | PF02560 | 4.2.1.104 | K01725 | AT3G23490.1 | CYN (CYANASE); DNA binding / cyanate hydratase/ hydro-lyase |
| gma-miR171k-3p | Glyma10g06280.1 | | | | AT3G45020.1 | 50S ribosomal protein-related |
| gma-miR167j | Glyma10g30370.1 | | | | | |
| gma-miR4382 | Glyma10g36350.1 | PF00076 | | | AT1G71800.1 | cleavage stimulation factor, putative |
| gma-miR4387b | Glyma11g06820.1 | PF00348 | 2.5.1.10 | K00795 | AT4G36810.1 | GGPS1 (GERANYLGERANYL PYROPHOSPHATE SYNTHASE 1); farnesyltranstransferase |
| gma-miR172b-5p | Glyma11g10790.1 | PF00076 | | | AT3G18610.1 | ATRANGAP1 (RAN GTPASE-ACTIVATING PROTEIN 1); nucleic acid binding / nucleotide binding |
| gma-miR1507a | Glyma11g21200.1 | PF00931,PF00560 | | | AT3G14470.1 | disease resistance protein (NBS-LRR class), putative |
| gma-miR164d | Glyma11g35750.1 | PF04187,PF11891 | | | AT3G56140.1 | unknown protein |
| gma-miR1521b | Glyma11g35800.1 | PF07107 | | | AT3G10985.1 | SAG20 (SENESCENCE ASSOCIATED GENE 20) |
| gma-miR4394 | Glyma11g35830.1 | PF01490 | | | AT3G56200.1 | amino acid transporter family protein |
| gma-miR156t | Glyma11g36980.1 | PF03110 | | | AT5G43270.1 | SPL2 (SQUAMOSA PROMOTER BINDING PROTEIN-LIKE 2); DNA binding / transcription factor |
| gma-miR166u | Glyma12g08080.1 | PF00046,PF00170,PF01852,PF08670 | | K09338 | AT5G60690.1 | REV (REVOLUTA); DNA binding / lipid binding / transcription factor |
| gma-miR4387b | Glyma12g09540.1 | PF04810,PF04811,PF04815 | | | AT2G27460.1 | sec23/sec24 transport family protein |
| gma-miR319f | Glyma12g20160.1 | PF03634 | | | AT1G53230.1 | TCP3; transcription factor |
| gma-miR408d | Glyma12g29270.1 | PF02891 | | | AT5G60410.1 | SIZ1; DNA binding / SUMO ligase |
| gma-miR319f | Glyma12g35720.1 | PF03634 | | | AT3G15030.1 | TCP4 (TCP family transcription factor 4); transcription factor |

| | | | | | | |
|---|---|---|---|---|---|---|
| gma-miR396g | Glyma13g07610.1 | PF12338,PF00101 | 4.1.1.39 | K01602 | AT5G38410.1 | ribulose bisphosphate carboxylase small chain 3B / RuBisCO small subunit 3B (RBCS-3B) (ATS3B) |
| gma-miR169n-5p | Glyma13g16770.2 | PF02045 | | | | |
| gma-miR408d | Glyma13g17840.1 | PF00628 | | | AT1G09520.1 | protein binding / zinc ion binding |
| gma-miR160f | Glyma13g20370.2 | PF02362,PF06507 | | | | |
| gma-miR4998 | Glyma13g21960.1 | PF01754,PF01428 | | | AT3G52800.1 | zinc finger (AN1-like) family protein |
| gma-miR159e-3p | Glyma13g33450.1 | PF10496 | | K08492 | AT1G51740.1 | SYP81 (SYNTAXIN OF PLANTS 81); SNAP receptor/ protein binding |
| gma-miR167f | Glyma14g03650.1 | PF02362,PF06507 ,PF02309 | | | AT1G30330.1 | ARF6 (AUXIN RESPONSE FACTOR 6); transcription factor |
| gma-miR4396 | Glyma14g07680.1 | PF02190 | | | AT1G75460.1 | ATP-dependent protease La (LON) domain-containing protein |
| gma-miR1516d | Glyma14g09620.1 | PF02704 | | | AT5G59845.1 | gibberellin-regulated family protein |
| gma-miR396i-5p | Glyma14g10100.1 | PF08880,PF08879 | | | AT2G22840.1 | AtGRF1 (GROWTH-REGULATING FACTOR 1); transcription activator |
| gma-miR319d | Glyma14g40630.1 | | | | AT5G42480.1 | ARC6 (ACCUMULATION AND REPLICATION OF CHLOROPLASTS 6); protein binding |
| gma-miR167a | Glyma15g00770.1 | PF07576,PF00097 ,PF02148 | | | AT2G26000.1 | zinc finger (C3HC4-type RING finger) family protein |
| gma-miR5374-5p | Glyma15g16510.1 | PF00571 | | | AT1G09020.1 | SNF4 (HOMOLOG OF YEAST SUCROSE NONFERMENTING 4); protein kinase activator |
| gma-miR396i-5p | Glyma15g19460.1 | PF08880,PF08879 | | | AT3G13960.1 | AtGRF5 (GROWTH-REGULATING FACTOR 5); transcription activator |
| gma-miR5035-5p | Glyma15g40450.1 | PF04725 | | K03541 | AT1G79040.1 | PSBR (photosystem II subunit R) |
| gma-miR164d | Glyma15g40510.1 | PF02365 | | | AT3G12977.1 | DNA binding |
| gma-miR160f | Glyma16g00660.1 | PF00153 | | | AT1G72820.1 | mitochondrial substrate carrier family protein |
| gma-miR156g | Glyma16g05900.1 | | | | | |
| gma-miR4376a-3p | Glyma17g05670.1 | PF08246,PF00112 | | | AT3G45310.1 | cysteine proteinase, putative |
| gma-miR2107 | Glyma17g10240.1 | PF01535 | | | AT1G74850.1 | PTAC2 (PLASTID TRANSCRIPTIONALLY ACTIVE2) |
| gma-miR159e-5p | Glyma17g17240.1 | | | | AT1G10140.1 | unknown protein |

| | | | | | | |
|---|---|---|---|---|---|---|
| gma-miR1508b | Glyma17g23900.1 | PF00009,PF03144,PF03143 | | | AT1G07930.1 | elongation factor 1-alpha / EF-1-alpha |
| gma-miR4369 | Glyma17g35890.1 | PF00076,PF00658 | | | AT1G49760.1 | PAB8 (POLY(A) BINDING PROTEIN 8); RNA binding / translation initiation factor |
| gma-miR1509b | Glyma18g03980.2 | PF07800 | | | | |
| gma-miR408b-5p | Glyma18g06320.1 | PF10236 | | | AT1G16870.1 | mitochondrial 28S ribosomal protein S29-related |
| gma-miR319c | Glyma18g18050.1 | PF12353,PF00076 | | K03248 | AT3G11400.1 | eukaryotic translation initiation factor 3G / eIF3g |
| gma-miR168b | Glyma18g44020.1 | PF04535,PF01284 | | | AT2G38480.1 | integral membrane protein, putative |
| gma-miR398c | Glyma18g52110.1 | PF00479,PF02781 | 1.1.1.49 | K00036 | AT5G13110.1 | G6PD2 (GLUCOSE-6-PHOSPHATE DEHYDROGENASE 2); glucose-6-phosphate dehydrogenase |
| gma-miR2111d | Glyma19g18360.2 | PF02201 | | | | |
| gma-miR156r | Glyma19g26390.1 | PF03110 | | | AT5G50570.1 | squamosa promoter-binding protein, putative |
| gma-miR156t | Glyma19g32800.1 | PF03110 | | | AT2G42200.1 | SPL9 (SQUAMOSA PROMOTER BINDING PROTEIN-LIKE 9); transcription factor |
| gma-miR5037a | Glyma20g23730.1 | PF00097 | | | AT3G19950.1 | zinc finger (C3HC4-type RING finger) family protein |
| gma-miR171c-5p | Glyma01g02950.1 | PF00153 | | | AT2G22500.1 | UCP5 (UNCOUPLING PROTEIN 5); binding |
| gma-miR156ab | Glyma01g29470.1 | PF00962 | 3.5.4.6 | K01490 | AT2G38280.1 | FAC1 (EMBRYONIC FACTOR1); AMP deaminase |
| gma-miR396c | Glyma01g34650.1 | PF08879,PF11722 | | | AT2G45480.1 | AtGRF9 (GROWTH-REGULATING FACTOR 9); transcription activator |
| gma-miR394b-3p | Glyma01g37430.1 | PF00067 | 1.14.-.- | K09755 | AT4G36220.1 | FAH1 (FERULIC ACID 5-HYDROXYLASE 1); ferulate 5-hydroxylase/ monooxygenase |
| gma-miR1507a | Glyma02g03010.1 | PF00931,PF00560 | | | AT3G14470.1 | disease resistance protein (NBS-LRR class), putative |
| gma-miR169p | Glyma02g35190.1 | PF02045 | | | AT5G06510.1 | NF-YA10 (NUCLEAR FACTOR Y, SUBUNIT A10); transcription factor |
| gma-miR156t | Glyma03g29900.1 | PF03110 | | | AT2G42200.1 | SPL9 (SQUAMOSA PROMOTER BINDING PROTEIN-LIKE 9); transcription factor |

| | | | | | |
|---|---|---|---|---|---|
| gma-miR172l | Glyma03g33470.1 | PF00847 | | AT4G36920.1 | AP2 (APETALA 2); transcription factor |
| gma-miR408d | Glyma03g40250.2 | | | | |
| gma-miR167f | Glyma03g42000.1 | PF07959,PF00288,PF08544 | | AT1G01220.1 | GHMP kinase-related |
| gma-miR1507a | Glyma04g29220.1 | PF00931,PF00560 | | AT3G14470.1 | disease resistance protein (NBS-LRR class), putative |
| gma-miR164d | Glyma04g33270.1 | PF02365 | | AT5G61430.1 | ANAC100 (ARABIDOPSIS NAC DOMAIN CONTAINING PROTEIN 100); transcription factor |
| gma-miR396i-5p | Glyma04g40880.1 | PF08879 | | AT5G53660.1 | AtGRF7 (GROWTH-REGULATING FACTOR 7); transcription activator |
| gma-miR393k | Glyma05g29700.1 | PF07653,PF00018 | | AT4G18060.1 | clathrin binding |
| gma-miR166t | Glyma05g30000.1 | PF00046,PF00170,PF01852,PF08670 | K09338 | AT2G34710.1 | PHB (PHABULOSA); DNA binding / transcription factor |
| gma-miR4409 | Glyma05g34570.1 | PF00333,PF03719 | K02981 | AT3G57490.1 | 40S ribosomal protein S2 (RPS2D) |
| gma-miR166u | Glyma05g35700.1 | PF00903 | | AT1G67280.1 | lactoylglutathione lyase, putative / glyoxalase I, putative |
| gma-miR319l | Glyma06g03820.2 | | | | |
| gma-miR5038b | Glyma06g05770.1 | PF00795 | | AT5G12040.1 | carbon-nitrogen hydrolase family protein |
| gma-miR395g | Glyma06g11150.1 | PF00916,PF01740 | | AT5G10180.1 | AST68; sulfate transmembrane transporter |
| gma-miR4375 | Glyma06g18080.1 | | | AT3G48500.1 | RNA binding |
| gma-miR4996 | Glyma06g20160.1 | PF01535 | | AT1G18900.1 | pentatricopeptide (PPR) repeat-containing protein |
| gma-miR1524 | Glyma06g35710.1 | PF00847 | | AT3G15210.1 | ERF4 (ETHYLENE RESPONSIVE ELEMENT BINDING FACTOR 4); DNA binding / protein binding / transcription factor/ transcription repressor |
| gma-miR2108a | Glyma07g04950.3 | PF00847 | | | |
| gma-miR167h | Glyma07g05870.1 | PF11789,PF04564,PF00514,PF02985 | | AT1G20780.1 | SAUL1 (SENESCENCE-ASSOCIATED E3 UBIQUITIN LIGASE 1); ubiquitin-protein ligase |

| | | | | | | |
|---|---|---|---|---|---|---|
| gma-miR169l-3p | Glyma07g14010.1 | PF04116,PF12076 | | | AT2G37700.1 | catalytic/ iron ion binding / oxidoreductase |
| gma-miR4993 | Glyma07g37790.1 | PF00168 | | | AT1G09070.1 | SRC2 (SOYBEAN GENE REGULATED BY COLD-2); protein binding |
| gma-miR5676 | Glyma07g39890.1 | PF03765,PF00650 | | | AT5G47730.1 | SEC14 cytosolic factor, putative / polyphosphoinositide-binding protein, putative |
| gma-miR167f | Glyma08g10550.2 | PF02362,PF06507 ,PF02309 | | | | |
| gma-miR2118b-3p | Glyma08g12860.1 | | | | AT5G46400.1 | PRP39-2 |
| gma-miR482a-3p | Glyma08g47740.1 | PF01417 | | | AT5G11710.1 | epsin N-terminal homology (ENTH) domain-containing protein / clathrin assembly protein-related |
| gma-miR4364b | Glyma09g01230.1 | PF00324 | | | AT1G17120.1 | CAT8 (CATIONIC AMINO ACID TRANSPORTER 8); basic amino acid transmembrane transporter/ cationic amino acid transmembrane transporter |
| gma-miR398c | Glyma09g06750.1 | PF00013 | | | AT4G26000.1 | PEP (PEPPER); RNA binding / nucleic acid binding |
| gma-miR169n-5p | Glyma09g07960.3 | PF02045 | | | | |
| gma-miR4413b | Glyma09g39260.1 | PF01535 | | | AT1G12700.1 | helicase domain-containing protein / pentatricopeptide (PPR) repeat-containing protein |
| gma-miR169p | Glyma10g10240.1 | PF02045 | | | AT5G06510.1 | NF-YA10 (NUCLEAR FACTOR Y, SUBUNIT A10); transcription factor |
| gma-miR166i-5p | Glyma10g38560.1 | | | | AT5G51840.1 | unknown protein |
| gma-miR4992 | Glyma11g02530.1 | PF00230 | | K09872 | AT1G01620.1 | PIP1C (PLASMA MEMBRANE INTRINSIC PROTEIN 1C); water channel |
| gma-miR169j-3p | Glyma11g03130.1 | PF03479 | | | AT4G22810.1 | DNA-binding protein-related |
| gma-miR1521b | Glyma11g04480.1 | | | | AT3G57990.1 | unknown protein |
| gma-miR4387c | Glyma11g06820.1 | PF00348 | 2.5.1.10 | K00795 | AT4G36810.1 | GGPS1 (GERANYLGERANYL PYROPHOSPHATE SYNTHASE 1); farnesyltranstransferase |
| gma-miR5672 | Glyma11g21020.1 | PF03018 | | | AT1G65870.1 | disease resistance-responsive family protein |

| | | | | | | |
|---|---|---|---|---|---|---|
| gma-miR5675 | Glyma12g04890.1 | PF00083,PF07690 | | | AT3G18830.1 | ATPLT5 (POLYOL TRANSPORTER 5); D-ribose transmembrane transporter/ D-xylose transmembrane transporter/ carbohydrate transmembrane transporter/ galactose transmembrane transporter/ glucose transmembrane transporter/ glycerol transmembrane transporter/ mann |
| gma-miR5674b | Glyma12g11560.1 | | | | AT4G28100.1 | unknown protein |
| gma-miR156r | Glyma12g27330.1 | PF03110 | | | AT3G60030.1 | SPL12 (squamosa promoter-binding protein-like 12); transcription factor |
| gma-miR396a-3p | Glyma12g28570.1 | PF02485 | | | AT1G10280.1 | unknown protein |
| gma-miR169d | Glyma13g16770.1 | PF02045 | | | AT1G72830.1 | NF-YA3 (NUCLEAR FACTOR Y, SUBUNIT A3); transcription factor |
| gma-miR1532 | Glyma13g23120.1 | PF02148,PF00443 | | | AT5G10790.1 | UBP22 (UBIQUITIN-SPECIFIC PROTEASE 22); ubiquitin thiolesterase/ ubiquitin-specific protease/ zinc ion binding |
| gma-miR4998 | Glyma13g28090.1 | PF01738 | | | AT3G23600.1 | dienelactone hydrolase family protein |
| gma-miR408b-5p | Glyma13g37700.1 | PF12609 | | | AT4G28240.1 | wound-responsive protein-related |
| gma-miR5374-5p | Glyma14g10900.1 | PF12146,PF00561 | | | AT5G11910.1 | esterase/lipase/thioesterase family protein |
| gma-miR408d | Glyma14g35530.1 | PF02298 | | | AT1G72230.1 | plastocyanin-like domain-containing protein |
| gma-miR4416a | Glyma15g06650.1 | PF01641 | | | AT4G21860.1 | MSRB2 (methionine sulfoxide reductase B 2); peptide-methionine-(S)-S-oxide reductase |
| gma-miR169n-5p | Glyma15g18970.1 | PF02045 | | | AT1G72830.1 | NF-YA3 (NUCLEAR FACTOR Y, SUBUNIT A3); transcription factor |
| gma-miR1516b | Glyma15g21860.1 | PF03110 | | | AT3G60030.1 | SPL12 (squamosa promoter-binding protein-like 12); transcription factor |
| gma-miR1514a-5p | Glyma16g01940.3 | | | | | |
| gma-miR390b-3p | Glyma16g04190.2 | PF00257 | | | | |
| gma-miR5372 | Glyma16g04950.2 | PF00722 | 2.4.1.207 | K08235 | | |

| | | | | | | |
|---|---|---|---|---|---|---|
| gma-miR1508c | Glyma16g32420.1 | PF01535 | | | AT1G12700.1 | helicase domain-containing protein / pentatricopeptide (PPR) repeat-containing protein |
| gma-miR156s | Glyma17g08840.1 | PF03110 | | | AT1G69170.1 | squamosa promoter-binding protein-like 6 (SPL6) |
| gma-miR172e | Glyma17g18640.1 | PF00847 | | K09284 | AT4G36920.1 | AP2 (APETALA 2); transcription factor |
| gma-miR169v | Glyma18g05890.1 | PF00234 | | | AT4G33355.1 | lipid binding |
| gma-miR169u | Glyma18g09170.1 | PF00931,PF00560 | | | AT3G07040.1 | RPM1 (RESISTANCE TO P. SYRINGAE PV MACULICOLA 1); nucleotide binding / protein binding |
| gma-miR166i-5p | Glyma18g29620.1 | PF00226 | | | AT5G64360.1 | DNAJ heat shock N-terminal domain-containing protein |
| gma-miR1521a | Glyma19g01050.3 | PF00484 | 4.2.1.1 | K01673 | | |
| gma-miR1510b-3p | Glyma19g07680.1 | PF01582,PF00931 ,PF00560 | | | AT1G72840.1 | ATP binding / protein binding / transmembrane receptor |
| gma-miR482d-3p | Glyma19g32150.1 | PF00931,PF00560 | | | AT3G14470.1 | disease resistance protein (NBS-LRR class), putative |
| gma-miR398c | Glyma19g42890.2 | PF00080 | 1.15.1.1 | K04565 | | |
| gma-miR172g | Glyma20g18980.2 | PF00108,PF02803 | | | | |
| gma-miR1510b-5p | Glyma20g35150.1 | PF00023,PF00097 | | | AT4G14365.1 | zinc finger (C3HC4-type RING finger) family protein / ankyrin repeat family protein |
| gma-miR169h | Glyma01g04530.1 | PF00098,PF00249 | | | AT1G70000.1 | DNA-binding family protein |
| gma-miR396i-5p | Glyma01g04610.2 | PF00010 | | | | |
| gma-miR171u | Glyma01g18100.1 | PF03514 | | | AT4G00150.1 | scarecrow-like transcription factor 6 (SCL6) |
| gma-miR396c | Glyma01g42670.1 | PF00314 | | | AT4G11650.1 | ATOSM34 (osmotin 34) |
| gma-miR5030 | Glyma01g44240.1 | PF00501 | | | AT1G20560.1 | AAE1 (ACYL ACTIVATING ENZYME 1); AMP binding |
| gma-miR1510b-3p | Glyma02g04750.1 | PF01582,PF00931 | | | AT5G17680.1 | disease resistance protein (TIR-NBS-LRR class), putative |
| gma-miR4414-5p | Glyma02g06610.1 | PF04678 | | | AT2G23790.1 | unknown protein |
| gma-miR5371-3p | Glyma02g09240.1 | PF04564 | | | AT5G09800.1 | U-box domain-containing protein |
| gma-miR4359a | Glyma02g16840.1 | PF00917,PF00651 | | | AT3G06190.1 | BPM2 (BTB-POZ AND MATH DOMAIN 2); protein binding |

| | | | | | | |
|---|---|---|---|---|---|---|
| gma-miR156g | Glyma02g30670.1 | PF03110 | | | AT2G42200.1 | SPL9 (SQUAMOSA PROMOTER BINDING PROTEIN-LIKE 9); transcription factor |
| gma-miR156t | Glyma02g30670.1 | PF03110 | | | AT2G42200.1 | SPL9 (SQUAMOSA PROMOTER BINDING PROTEIN-LIKE 9); transcription factor |
| gma-miR4353 | Glyma02g38410.1 | PF00561 | | | AT3G10870.1 | MES17 (METHYL ESTERASE 17); hydrolase/ hydrolase, acting on ester bonds / methyl indole-3-acetate esterase |
| gma-miR169n-5p | Glyma02g47380.1 | PF02045 | | | AT3G20910.1 | NF-YA9 (NUCLEAR FACTOR Y, SUBUNIT A9); specific transcriptional repressor/ transcription factor |
| gma-miR399h | Glyma03g31950.1 | PF00083,PF07690 | | K08176 | AT3G54700.1 | carbohydrate transmembrane transporter/ phosphate transmembrane transporter/ sugar:hydrogen symporter |
| gma-miR166t | Glyma03g34950.2 | PF00274 | 4.1.2.13 | K01623 | | |
| gma-miR530e | Glyma03g41900.1 | | | | AT1G01260.1 | basic helix-loop-helix (bHLH) family protein |
| gma-miR4406 | Glyma03g42310.1 | PF00504 | | K08908 | AT3G61470.1 | LHCA2; chlorophyll binding |
| gma-miR167f | Glyma04g10970.2 | | | | | |
| gma-miR1507a | Glyma04g29220.2 | PF00931,PF00560 | | | | |
| gma-miR156s | Glyma04g37390.1 | PF03110 | | | AT5G50670.1 | squamosa promoter-binding protein, putative |
| gma-miR1510b-3p | Glyma05g09440.1 | PF00931,PF00560 | | | AT5G66900.1 | disease resistance protein (CC-NBS-LRR class), putative |
| gma-miR5374-5p | Glyma05g09440.2 | PF00931,PF00560 | | | | |
| gma-miR1510b-3p | Glyma05g09440.2 | PF00931,PF00560 | | | | |
| gma-miR4353 | Glyma05g30600.1 | PF06298 | | K02723 | AT1G67740.1 | PSBY |
| gma-miR156y | Glyma05g38180.5 | PF03110 | | | | |
| gma-miR164k | Glyma06g21020.1 | PF02365 | | | AT5G61430.1 | ANAC100 (ARABIDOPSIS NAC DOMAIN CONTAINING PROTEIN 100); transcription factor |
| gma-miR4372b | Glyma06g29540.1 | PF00684 | | | AT3G47650.1 | bundle-sheath defective protein 2 family / bsd2 family |

| | | | | | | |
|---|---|---|---|---|---|---|
| gma-miR319f | Glyma06g34330.1 | PF03634 | | | AT1G53230.1 | TCP3; transcription factor |
| gma-miR164k | Glyma06g35660.1 | PF02365 | | | AT5G53950.1 | CUC2 (CUP-SHAPED COTYLEDON 2); transcription factor |
| gma-miR156f | Glyma06g39800.1 | PF00582 | | | AT5G54430.1 | universal stress protein (USP) family protein |
| gma-miR169v | Glyma07g04050.2 | PF02045 | | | | |
| gma-miR1514a-5p | Glyma07g05360.1 | PF02365 | | | AT4G35580.1 | NTL9 (NAC transcription factor-like 9); transcription factor |
| gma-miR1514a-5p | Glyma07g05370.1 | PF02365 | | | AT4G35580.1 | NTL9 (NAC transcription factor-like 9); transcription factor |
| gma-miR4996 | Glyma07g31310.1 | PF00264,PF12142,PF12143 | | | | |
| gma-miR156r | Glyma07g31880.1 | PF03110 | | | AT2G33810.1 | SPL3 (SQUAMOSA PROMOTER BINDING PROTEIN-LIKE 3); DNA binding / transcription factor |
| gma-miR156y | Glyma08g01450.1 | PF03110 | | | AT5G50670.1 | squamosa promoter-binding protein, putative |
| gma-miR169r | Glyma08g21550.1 | | | | AT1G79760.1 | DTA4 (DOWNSTREAM TARGET OF AGL15-4) |
| gma-miR166u | Glyma08g21620.1 | PF00046,PF00170,PF01852,PF08670 | | K09338 | AT1G52150.1 | ATHB-15; DNA binding / transcription factor |
| gma-miR1513b | Glyma08g27950.1 | PF00646 | | | AT3G23880.1 | F-box family protein |
| gma-miR5372 | Glyma09g02600.1 | PF00141 | 1.11.1.7 | K00430 | AT2G38380.1 | peroxidase 22 (PER22) (P22) (PRXEA) / basic peroxidase E |
| gma-miR1515b | Glyma09g02920.1 | PF00271,PF03368,PF02170,PF00636,PF00035 | | | AT3G03300.1 | DCL2 (DICER-LIKE 2); ATP binding / ATP-dependent helicase/ RNA binding / double-stranded RNA binding / helicase/ nucleic acid binding / ribonuclease III |
| gma-miR396c | Glyma09g07990.1 | PF08880,PF08879 | | | AT3G13960.1 | AtGRF5 (GROWTH-REGULATING FACTOR 5); transcription activator |
| gma-miR5033 | Glyma09g34310.2 | PF01399 | | K03039 | | |
| gma-miR4387e | Glyma09g36760.1 | PF03517 | | | AT5G62290.1 | nucleotide-sensitive chloride conductance regulator (ICln) family protein |

| | | | | | | |
|---|---|---|---|---|---|---|
| gma-miR399h | Glyma10g04230.1 | PF00083,PF07690 | | K08176 | AT2G38940.1 | ATPT2 (ARABIDOPSIS THALIANA PHOSPHATE TRANSPORTER 2); carbohydrate transmembrane transporter/ inorganic phosphate transmembrane transporter/ phosphate transmembrane transporter/ sugar:hydrogen symporter |
| gma-miR5042-5p | Glyma10g05090.1 | PF01960 | 2.3.1.1,2.3.1.3 | K00620,K00642 | AT2G37500.1 | arginine biosynthesis protein ArgJ family |
| gma-miR160f | Glyma10g06080.1 | PF02362,PF06507 | | | AT4G30080.1 | ARF16 (AUXIN RESPONSE FACTOR 16); miRNA binding / transcription factor |
| gma-miR2108a | Glyma10g13450.1 | PF00139 | | | AT5G10530.1 | lectin protein kinase, putative |
| gma-miR171i-3p | Glyma11g06980.1 | PF03514 | | | AT4G36710.1 | transcription factor |
| gma-miR171c-5p | Glyma11g11700.1 | PF06094 | | | AT3G02910.1 | unknown protein |
| gma-miR171u | Glyma11g17490.1 | PF03514 | | | AT4G00150.1 | scarecrow-like transcription factor 6 (SCL6) |
| gma-miR1535b | Glyma11g30740.1 | PF00249 | | | AT1G15720.1 | TRFL5 (TRF-LIKE 5); DNA binding / transcription factor |
| gma-miR4394 | Glyma11g34410.1 | PF00481 | | | AT3G11410.1 | PP2CA (ARABIDOPSIS THALIANA PROTEIN PHOSPHATASE 2CA); protein binding / protein serine/threonine phosphatase |
| gma-miR164k | Glyma12g35530.1 | PF02365 | | | AT5G53950.1 | CUC2 (CUP-SHAPED COTYLEDON 2); transcription factor |
| gma-miR159e-3p | Glyma13g04030.1 | PF00249 | | | AT5G06100.1 | MYB33 (MYB DOMAIN PROTEIN 33); DNA binding / transcription factor |
| gma-miR396i-5p | Glyma13g16920.1 | PF08880,PF08879 | | | AT3G13960.1 | AtGRF5 (GROWTH-REGULATING FACTOR 5); transcription activator |

| | | | | | | |
|---|---|---|---|---|---|---|
| gma-miR5034 | Glyma13g23260.1 | PF01458 | | K07033 | AT4G04770.1 | ATABC1 (ATP BINDING CASSETTE PROTEIN 1); ATPase, coupled to transmembrane movement of substances / protein binding / transporter |
| gma-miR4996 | Glyma13g25260.1 | PF00264,PF12142 ,PF12143 | | | | |
| gma-miR159f-3p | Glyma13g25720.1 | | | | AT3G11440.1 | MYB65 (MYB DOMAIN PROTEIN 65); DNA binding / transcription factor |
| gma-miR319h | Glyma13g29160.1 | PF03634 | | | AT4G18390.1 | TCP family transcription factor, putative |
| gma-miR1516b | Glyma13g31060.3 | PF05564 | | | | |
| gma-miR319f | Glyma13g34690.1 | PF03634 | | | AT3G15030.1 | TCP4 (TCP family transcription factor 4); transcription factor |
| gma-miR164d | Glyma13g34950.1 | PF02365 | | | AT5G53950.1 | CUC2 (CUP-SHAPED COTYLEDON 2); transcription factor |
| gma-miR862b | Glyma14g06220.1 | PF00462 | | | AT5G58530.1 | glutaredoxin family protein |
| gma-miR1520q | Glyma14g07360.1 | PF03330,PF01357 | | | AT2G28950.1 | ATEXPA6 (ARABIDOPSIS THALIANA EXPANSIN A6) |
| gma-miR3522 | Glyma14g07470.1 | PF05282 | | | AT1G66510.1 | AAR2 protein family |
| gma-miR398c | Glyma14g39910.1 | PF02897,PF00326 | 3.4.21.26 | K01322 | AT1G76140.1 | serine-type endopeptidase/ serine-type peptidase |
| gma-miR319f | Glyma15g09910.1 | PF03634 | | | AT4G18390.1 | TCP family transcription factor, putative |
| gma-miR159e-3p | Glyma15g35860.1 | PF00249 | | | AT3G11440.1 | MYB65 (MYB DOMAIN PROTEIN 65); DNA binding / transcription factor |
| gma-miR862a | Glyma16g00210.1 | | 2.1.1.12 | K08247 | AT5G49810.1 | MMT; S-adenosylmethionine-dependent methyltransferase |
| gma-miR396e | Glyma16g00970.1 | PF08880,PF08879 | | | AT3G13960.1 | AtGRF5 (GROWTH-REGULATING FACTOR 5); transcription activator |
| gma-miR1514a-5p | Glyma16g01930.2 | | | | | |
| gma-miR390b-3p | Glyma16g29400.1 | | | | AT3G16520.1 | UDP-glucoronosyl/UDP-glucosyl transferase family protein |
| gma-miR396e | Glyma17g05800.1 | PF08879 | | | AT3G13960.1 | AtGRF5 (GROWTH-REGULATING FACTOR 5); transcription activator |

| | | | | | | |
|---|---|---|---|---|---|---|
| gma-miR169n-5p | Glyma17g05920.1 | PF02045 | | | AT1G17590.1 | NF-YA8 (NUCLEAR FACTOR Y, SUBUNIT A8); transcription factor |
| gma-miR164d | Glyma17g10970.1 | PF02365 | | | AT5G61430.1 | ANAC100 (ARABIDOPSIS NAC DOMAIN CONTAINING PROTEIN 100); transcription factor |
| gma-miR168b | Glyma17g14160.1 | PF03634 | | | AT2G45680.1 | TCP family transcription factor, putative |
| gma-miR1510b-3p | Glyma17g20860.1 | PF00931,PF00560 | | | AT5G66900.1 | disease resistance protein (CC-NBS-LRR class), putative |
| gma-miR396c | Glyma17g35100.1 | PF08880,PF08879 | | | AT2G22840.1 | AtGRF1 (GROWTH-REGULATING FACTOR 1); transcription activator |
| gma-miR394g | Glyma18g00870.1 | PF00646 | | | AT1G27340.1 | F-box family protein |
| gma-miR156t | Glyma18g00890.1 | | | | | |
| gma-miR862a | Glyma18g07190.1 | PF05340 | | | AT3G46990.1 | unknown protein |
| gma-miR5675 | Glyma18g40730.1 | | | | AT3G49910.1 | 60S ribosomal protein L26 (RPL26A) |
| gma-miR4375 | Glyma18g46830.1 | | | | AT2G45990.1 | unknown protein |
| gma-miR4375 | Glyma18g47710.1 | PF02605 | | K02699 | AT4G12800.1 | PSAL (photosystem I subunit L) |
| gma-miR156g | Glyma19g26390.1 | PF03110 | | | AT5G50570.1 | squamosa promoter-binding protein, putative |
| gma-miR396c | Glyma19g30350.1 | PF02915 | 1.14.13.81 | K04035 | AT3G56940.1 | CRD1 (COPPER RESPONSE DEFECT 1); DNA binding / magnesium-protoporphyrin IX monomethyl ester (oxidative) cyclase |
| gma-miR5037b | Glyma19g33700.1 | PF01423 | | K11087 | AT4G02840.1 | small nuclear ribonucleoprotein D1, putative / snRNP core protein D1, putative / Sm protein D1, putative |
| gma-miR159e-3p | Glyma19g40720.1 | | | | AT2G47160.1 | BOR1 (REQUIRES HIGH BORON 1); anion exchanger/ boron transporter |
| gma-miR159f-3p | Glyma20g11040.1 | PF00249 | | | AT5G06100.1 | MYB33 (MYB DOMAIN PROTEIN 33); DNA binding / transcription factor |
| gma-miR4376a-3p | Glyma20g24990.2 | PF00828 | | K02876 | | |
| gma-miR395g | Glyma20g28980.1 | PF01747 | 2.7.7.4 | K00958 | AT3G22890.1 | APS1 (ATP SULFURYLASE 1); sulfate adenylyltransferase (ATP) |

| | | | | | | |
|---|---|---|---|---|---|---|
| gma-miR1512b | Glyma20g39080.1 | PF09598,PF04774 | | | AT4G16830.1 | nuclear RNA-binding protein (RGGA) |
| gma-miR5372 | Glyma01g00860.2 | PF01217 | | | | |
| gma-miR1515b | Glyma01g36660.1 | PF00023 | | | AT5G66055.1 | AKRP (ANKYRIN REPEAT PROTEIN); protein binding |
| gma-miR4387a | Glyma02g06120.1 | PF00977 | 5.3.1.16 | K01814 | AT2G36230.1 | APG10 (ALBINO AND PALE GREEN 10); 1-(5-phosphoribosyl)-5-[(5-phosphoribosylamino)methylideneamino]imidazole-4-carboxamide isomerase |
| gma-miR5370 | Glyma02g11100.1 | PF01501 | | | AT1G13250.1 | GATL3 (Galacturonosyltransferase-like 3); polygalacturonate 4-alpha-galacturonosyltransferase/ transferase, transferring glycosyl groups / transferase, transferring hexosyl groups |
| gma-miR167a | Glyma02g18250.1 | PF08711 | | | AT5G05140.1 | transcription elongation factor-related |
| gma-miR167a | Glyma02g18250.2 | PF08711 | | | | |
| gma-miR167a | Glyma02g18250.3 | PF08711 | | | | |
| gma-miR171l | Glyma02g37610.1 | | | | AT1G09750.1 | chloroplast nucleoid DNA-binding protein-related |
| gma-miR171u | Glyma02g40840.1 | PF00282 | 4.1.1.15 | K01580 | AT5G17330.1 | GAD; calmodulin binding / glutamate decarboxylase |
| gma-miR1515b | Glyma02g42020.1 | PF00097 | | | AT5G05830.1 | zinc finger (C3HC4-type RING finger) family protein |
| gma-miR4382 | Glyma02g42360.2 | PF03226 | | | | |
| gma-miR4382 | Glyma02g42360.3 | PF03226 | | | | |
| gma-miR5037a | Glyma02g42600.1 | | | | | |
| gma-miR4397-5p | Glyma03g32620.4 | PF00400 | | | | |
| gma-miR2118b-3p | Glyma03g33710.1 | PF07719,PF00515,PF00226 | | | AT5G03160.1 | ATP58IPK (ARABIDOPSIS HOMOLOG OF MAMALLIAN P58IPK); binding / heat shock protein binding |
| gma-miR396e | Glyma03g35010.1 | PF08880,PF08879 | | | AT2G36400.1 | AtGRF3 (GROWTH-REGULATING FACTOR 3); transcription activator |

| | | | | | |
|---|---|---|---|---|---|
| gma-miR171c-5p | Glyma04g01040.1 | PF00628,PF02201,PF03126,PF02213,PF00642 | | AT2G16485.1 | DNA binding / nucleic acid binding / protein binding / zinc ion binding |
| gma-miR159f-3p | Glyma04g15150.1 | PF00249 | | AT5G06100.1 | MYB33 (MYB DOMAIN PROTEIN 33); DNA binding / transcription factor |
| gma-miR5371-3p | Glyma04g36710.1 | PF02364 | | AT2G36850.1 | GSL8 (GLUCAN SYNTHASE-LIKE 8); 1,3-beta-glucan synthase/ transferase, transferring glycosyl groups |
| gma-miR156f | Glyma05g00200.1 | PF03110 | | AT1G69170.1 | squamosa promoter-binding protein-like 6 (SPL6) |
| gma-miR1523b | Glyma05g02010.1 | | | AT3G48020.1 | unknown protein |
| gma-miR4376a-3p | Glyma05g24470.1 | PF00403 | | AT4G33520.1 | PAA1 (P-TYPE ATP-ASE 1); ATPase, coupled to transmembrane movement of ions, phosphorylative mechanism / copper ion transmembrane transporter |
| gma-miR159e-3p | Glyma05g27370.1 | PF03634 | | AT4G18390.1 | TCP family transcription factor, putative |
| gma-miR394g | Glyma05g28050.1 | PF00646 | | AT1G27340.1 | F-box family protein |
| gma-miR166j-5p | Glyma05g37580.1 | PF00097 | | AT3G61460.1 | BRH1 (BRASSINOSTEROID-RESPONSIVE RING-H2); protein binding / zinc ion binding |
| gma-miR156y | Glyma05g38180.4 | PF03110 | | | |
| gma-miR4353 | Glyma06g09500.1 | PF00091,PF03953 | K07374 | AT1G50010.1 | TUA2; structural constituent of cytoskeleton |
| gma-miR319d | Glyma06g15180.1 | PF01926,PF02824 | | AT4G39520.1 | GTP-binding protein, putative |
| gma-miR319d | Glyma06g15180.2 | PF01926,PF02824 | | | |
| gma-miR167a | Glyma06g18070.1 | PF00439 | | AT5G63320.1 | unknown protein |
| gma-miR5371-5p | Glyma06g23620.1 | PF01535 | | AT5G55740.1 | CRR21 (chlororespiratory reduction 21) |
| gma-miR5036 | Glyma06g39690.1 | PF01412 | | AT5G54310.1 | AGD5 (ARF-GAP domain 5); ARF GTPase activator/ DNA binding / zinc ion binding |
| gma-miR159f-3p | Glyma06g47000.1 | PF00249 | | AT3G11440.1 | MYB65 (MYB DOMAIN PROTEIN 65); DNA binding / transcription factor |

| | | | | | |
|---|---|---|---|---|---|
| gma-miR166u | Glyma07g01940.1 | PF00046,PF01852,PF08670 | K09338 | AT1G52150.1 | ATHB-15; DNA binding / transcription factor |
| gma-miR166t | Glyma07g01940.3 | PF00046,PF01852 | | | |
| gma-miR396e | Glyma07g04290.1 | PF08880,PF08879 | | AT3G13960.1 | AtGRF5 (GROWTH-REGULATING FACTOR 5); transcription activator |
| gma-miR1514a-5p | Glyma07g05360.2 | PF02365 | | | |
| gma-miR156f | Glyma07g07870.1 | PF02178,PF03479 | | AT2G45850.1 | DNA-binding family protein |
| gma-miR396c | Glyma07g32230.1 | PF08263,PF00560,PF00069,PF07714 | K00924 | AT1G28440.1 | HSL1 (HAESA-Like 1); ATP binding / kinase/ protein serine/threonine kinase |
| gma-miR166t | Glyma08g13110.1 | PF00046,PF00170,PF01852,PF08670 | K09338 | AT2G34710.1 | PHB (PHABULOSA); DNA binding / transcription factor |
| gma-miR166t | Glyma08g13110.2 | PF00046,PF00170,PF01852 | K09338 | | |
| gma-miR166u | Glyma08g21610.1 | PF00046,PF01852,PF08670 | K09338 | AT1G52150.1 | ATHB-15; DNA binding / transcription factor |
| gma-miR166j-3p | Glyma08g21620.1 | PF00046,PF00170,PF01852,PF08670 | K09338 | AT1G52150.1 | ATHB-15; DNA binding / transcription factor |
| gma-miR166j-3p | Glyma08g21620.2 | PF00046,PF00170,PF01852,PF08670 | K09338 | | |
| gma-miR166u | Glyma08g21620.2 | PF00046,PF00170,PF01852,PF08670 | K09338 | | |
| gma-miR862b | Glyma08g40360.1 | PF02181 | | AT1G59910.1 | formin homology 2 domain-containing protein / FH2 domain-containing protein |
| gma-miR396c | Glyma09g00410.1 | PF01253 | | AT1G71350.1 | eukaryotic translation initiation factor SUI1 family protein |
| gma-miR5371-3p | Glyma09g01250.1 | PF02298 | | AT3G27200.1 | plastocyanin-like domain-containing protein |
| gma-miR4376-5p | Glyma09g31590.1 | PF04842 | | AT1G48840.1 | unknown protein |
| gma-miR396g | Glyma10g03390.1 | PF00190,PF07883 | | AT3G22640.1 | PAP85; nutrient reservoir |

| | | | | | | |
|---|---|---|---|---|---|---|
| gma-miR482a-3p | Glyma10g35960.1 | PF00364,PF02817,PF00198 | 2.3.1.12 | K00627 | AT1G34430.1 | EMB3003 (embryo defective 3003); acyltransferase/ dihydrolipoyllysine-residue acetyltransferase/ protein binding |
| gma-miR5371-5p | Glyma11g04420.1 | PF04133 | | | AT1G32410.1 | vacuolar protein sorting 55 family protein / VPS55 family protein |
| gma-miR4378a | Glyma11g09330.2 | PF00368 | 1.1.1.34 | K00021 | | |
| gma-miR394g | Glyma11g36960.1 | PF00646 | | | AT1G27340.1 | F-box family protein |
| gma-miR1515b | Glyma12g12180.1 | PF00481 | | | AT4G03415.1 | catalytic/ protein serine/threonine phosphatase |
| gma-miR2118b-3p | Glyma12g28730.2 | PF00069 | | | | |
| gma-miR319d | Glyma12g33640.1 | PF03634 | | | AT3G15030.1 | TCP4 (TCP family transcription factor 4); transcription factor |
| gma-miR482d-3p | Glyma12g34690.1 | PF00931,PF00560 | | | AT4G27220.1 | disease resistance protein (NBS-LRR class), putative |
| gma-miR172j | Glyma12g34940.1 | PF12584 | | | AT5G54440.1 | unknown protein |
| gma-miR1520l | Glyma13g01580.1 | PF00335 | | | AT2G23810.1 | TET8 (TETRASPANIN8) |
| gma-miR159f-3p | Glyma13g04030.1 | PF00249 | | | AT5G06100.1 | MYB33 (MYB DOMAIN PROTEIN 33); DNA binding / transcription factor |
| gma-miR396c | Glyma13g16920.1 | PF08880,PF08879 | | | AT3G13960.1 | AtGRF5 (GROWTH-REGULATING FACTOR 5); transcription activator |
| gma-miR1515b | Glyma13g20520.1 | | | | AT2G36895.1 | unknown protein |
| gma-miR3522 | Glyma13g20940.1 | PF00230 | | | AT2G36830.1 | GAMMA-TIP (GAMMA TONOPLAST INTRINSIC PROTEIN); water channel |
| gma-miR4387b | Glyma13g23190.1 | PF03662 | | | AT5G34940.1 | AtGUS3 (Arabidopsis thaliana glucuronidase 3); beta-glucuronidase |
| gma-miR159e-5p | Glyma13g38340.1 | | | | | |
| gma-miR169l-5p | Glyma14g01360.1 | PF02045 | | | AT3G20910.1 | NF-YA9 (NUCLEAR FACTOR Y, SUBUNIT A9); specific transcriptional repressor/ transcription factor |
| gma-miR160f | Glyma14g33730.1 | PF02362,PF06507 | | | AT1G77850.1 | ARF17 (AUXIN RESPONSE FACTOR 17); transcription factor |

| | | | | | | |
|---|---|---|---|---|---|---|
| gma-miR167i | Glyma14g37210.1 | PF01255 | | | AT5G58770.1 | dehydrodolichyl diphosphate synthase, putative / DEDOL-PP synthase, putative |
| gma-miR393k | Glyma15g08350.2 | PF11721,PF00560 | | | | |
| gma-miR159e-3p | Glyma15g08630.1 | | | | AT2G34010.1 | unknown protein |
| gma-miR5372 | Glyma15g13500.1 | PF00141 | 1.11.1.7 | K00430 | AT2G38380.1 | peroxidase 22 (PER22) (P22) (PRXEA) / basic peroxidase E |
| gma-miR1514a-5p | Glyma15g13580.1 | PF04499 | | | AT1G30470.1 | SIT4 phosphatase-associated family protein |
| gma-miR167f | Glyma15g16650.1 | PF02469 | | | AT3G46550.1 | SOS5 (salt overly sensitive 5); polysaccharide binding / protein binding |
| gma-miR319l | Glyma15g35860.1 | PF00249 | | | AT3G11440.1 | MYB65 (MYB DOMAIN PROTEIN 65); DNA binding / transcription factor |
| gma-miR159f-3p | Glyma15g35860.1 | PF00249 | | | AT3G11440.1 | MYB65 (MYB DOMAIN PROTEIN 65); DNA binding / transcription factor |
| gma-miR1514a-5p | Glyma16g01930.1 | PF02365 | | | AT4G35580.1 | NTL9 (NAC transcription factor-like 9); transcription factor |
| gma-miR4404 | Glyma16g04240.3 | PF08267,PF01717 | | | | |
| gma-miR5370 | Glyma16g07280.1 | | | | AT5G14390.1 | unknown protein |
| gma-miR171l | Glyma17g31440.1 | | | | AT1G12310.1 | calmodulin, putative |
| gma-miR5675 | Glyma18g00630.2 | PF00722,PF06955 | 2.4.1.207 | K08235 | | |
| gma-miR1523b | Glyma18g02340.1 | PF01269 | | | AT4G25630.1 | FIB2 (FIBRILLARIN 2); snoRNA binding |
| gma-miR482d-3p | Glyma18g14400.1 | PF00400 | | | AT5G08560.1 | transducin family protein / WD-40 repeat family protein |
| gma-miR156t | Glyma18g36960.1 | PF03110 | | | AT2G42200.1 | SPL9 (SQUAMOSA PROMOTER BINDING PROTEIN-LIKE 9); transcription factor |
| gma-miR156m | Glyma19g02020.1 | | | | AT1G74860.1 | unknown protein |

| | | | | | | |
|---|---|---|---|---|---|---|
| gma-miR167f | Glyma19g33690.1 | PF00782,PF01331,PF03919 | | | AT5G01290.1 | mRNA guanylyltransferase/ phosphatase/ polynucleotide 5\'-phosphatase/ protein tyrosine phosphatase/ protein tyrosine/serine/threonine phosphatase |
| gma-miR396c | Glyma19g34250.1 | PF00690,PF00122,PF00702,PF00689 | 3.6.3.8 | K01537 | AT3G63380.1 | calcium-transporting ATPase, plasma membrane-type, putative / Ca(2+)-ATPase, putative (ACA12) |
| gma-miR396e | Glyma19g37740.1 | PF08880,PF08879 | | | AT2G36400.1 | AtGRF3 (GROWTH-REGULATING FACTOR 3); transcription activator |
| gma-miR396e | Glyma19g37740.2 | PF08880,PF08879 | | | | |
| gma-miR172j | Glyma20g24850.2 | PF00627,PF09280 | | | | |
| gma-miR4993 | Glyma20g25760.1 | | | | AT1G15100.1 | RHA2A; protein binding / ubiquitin-protein ligase/ zinc ion binding |
| gma-miR395g | Glyma20g28980.2 | PF01747 | 2.7.7.4 | K00958 | | |
| gma-miR160a-3p | Glyma20g33390.1 | PF02365 | | | AT3G10500.1 | anac053 (Arabidopsis NAC domain containing protein 53); transcription factor |
| gma-miR169h | Glyma20g36010.1 | PF00628 | | | AT4G10600.1 | PHD finger family protein |
| gma-miR396e | Glyma01g35540.1 | PF03759 | | | AT1G31650.1 | ROPGEF14; Rho guanyl-nucleotide exchange factor |
| gma-miR172g | Glyma03g39940.1 | | | | AT1G03220.1 | extracellular dermal glycoprotein, putative / EDGP, putative |
| gma-miR4378b | Glyma04g03120.1 | PF01301 | | | AT4G36360.1 | BGAL3 (beta-galactosidase 3); beta-galactosidase/ catalytic/ cation binding / sugar binding |
| gma-miR166t | Glyma04g09000.1 | PF01852,PF08670 | | | AT4G32880.1 | ATHB-8 (HOMEOBOX GENE 8); DNA binding / transcription factor |
| gma-miR159e-3p | Glyma04g15150.1 | PF00249 | | | AT5G06100.1 | MYB33 (MYB DOMAIN PROTEIN 33); DNA binding / transcription factor |
| gma-miR5375 | Glyma04g40180.1 | PF08263,PF00560,PF07714,PF00069 | | | AT5G58300.1 | leucine-rich repeat transmembrane protein kinase, putative |
| gma-miR5369 | Glyma05g26100.1 | PF00004 | | | AT2G34560.1 | katanin, putative |
| gma-miR5676 | Glyma05g30170.2 | | | | | |

| | | | | | | |
|---|---|---|---|---|---|---|
| gma-miR171c-5p | Glyma06g04370.1 | PF03763 | | | AT4G36970.1 | remorin family protein |
| gma-miR1524 | Glyma06g12720.2 | | | | | |
| gma-miR5371-3p | Glyma06g47220.1 | PF00170,PF07716 | | | AT3G56850.1 | AREB3 (ABA-RESPONSIVE ELEMENT BINDING PROTEIN 3); DNA binding / transcription activator/ transcription factor |
| gma-miR399g | Glyma07g28940.1 | PF03181 | | | AT5G25610.1 | RD22; nutrient reservoir |
| gma-miR1516d | Glyma08g11260.1 | | | | AT3G57450.1 | unknown protein |
| gma-miR4387e | Glyma08g11490.2 | PF00464 | 2.1.2.1 | K00600 | | |
| gma-miR5380b | Glyma08g12270.1 | PF08246,PF00112 | | | AT1G09850.1 | XBCP3 (xylem bark cysteine peptidase 3); cysteine-type endopeptidase/ cysteine-type peptidase |
| gma-miR5380b | Glyma08g12280.1 | PF08246,PF00112 | | | AT1G09850.1 | XBCP3 (xylem bark cysteine peptidase 3); cysteine-type endopeptidase/ cysteine-type peptidase |
| gma-miR1515b | Glyma09g27640.1 | PF00069 | | | AT4G25390.1 | protein kinase family protein |
| gma-miR169j-3p | Glyma10g38830.1 | PF08423 | | K10872 | AT3G22880.1 | DMC1 (DISRUPTION OF MEIOTIC CONTROL 1); ATP binding / DNA binding / DNA-dependent ATPase/ damaged DNA binding / nucleoside-triphosphatase/ nucleotide binding / protein binding |
| gma-miR159e-3p | Glyma10g39170.1 | PF00190 | | | AT3G22640.1 | PAP85; nutrient reservoir |
| gma-miR4415a-5p | Glyma10g39420.1 | PF03765 | | | AT1G05370.1 | unknown protein |
| gma-miR5371-3p | Glyma10g43840.1 | | | | AT5G13560.1 | unknown protein |
| gma-miR5037a | Glyma11g11430.1 | PF06911 | | | AT2G17840.1 | ERD7 (EARLY-RESPONSIVE TO DEHYDRATION 7) |
| gma-miR394b-3p | Glyma12g00350.1 | PF00919,PF04055 | | | AT1G72090.1 | radical SAM domain-containing protein / TRAM domain-containing protein |
| gma-miR1523b | Glyma12g10270.1 | PF00515,PF07719 | | | AT5G21990.1 | tetratricopeptide repeat (TPR)-containing protein |
| gma-miR5043 | Glyma13g02840.1 | PF03514 | | | AT4G08250.1 | scarecrow transcription factor family protein |
| gma-miR399h | Glyma13g20210.4 | PF00097 | | | | |

| | | | | | | |
|---|---|---|---|---|---|---|
| gma-miR4415a-5p | Glyma13g23790.4 | PF00389,PF02826 | 1.2.1.2 | K00122 | | |
| gma-miR396k-3p | Glyma13g42310.1 | PF01477,PF00305 | | | AT1G55020.1 | LOX1; lipoxygenase |
| gma-miR4997 | Glyma14g02550.1 | | | | AT5G55060.1 | unknown protein |
| gma-miR171j-5p | Glyma14g26410.1 | PF00228 | | | | |
| gma-miR482a-3p | Glyma15g18730.4 | PF11705 | | | | |
| gma-miR482d-3p | Glyma15g42510.1 | PF00590 | 2.1.1.107 | K02302 | AT5G40850.1 | UPM1 (UROPHORPHYRIN METHYLASE 1); uroporphyrin-III C-methyltransferase |
| gma-miR156f | Glyma17g02620.1 | | | | AT4G15640.1 | unknown protein |
| gma-miR5672 | Glyma17g03020.1 | PF00225 | | | AT3G16630.1 | KINESIN-13A; ATP binding / microtubule motor |
| gma-miR1517 | Glyma17g20200.1 | PF02234 | | | AT1G49620.1 | ICK5; cyclin binding / cyclin-dependent protein kinase inhibitor |
| gma-miR394g | Glyma18g00870.2 | PF00646 | | | | |
| gma-miR1509a | Glyma18g03980.2 | PF07800 | | | | |
| gma-miR1510b-3p | Glyma19g07650.1 | PF01582,PF00931,PF05729,PF00560 | | | AT5G36930.1 | disease resistance protein (TIR-NBS-LRR class), putative |
| gma-miR5672 | Glyma19g40410.1 | PF00326 | | | AT2G47390.1 | serine-type endopeptidase/ serine-type peptidase |
| gma-miR5371-3p | Glyma19g41440.1 | | | | AT1G56700.1 | pyrrolidone-carboxylate peptidase family protein |
| gma-miR162c | Glyma19g45060.1 | PF04851,PF00270,PF00271,PF03368,PF02170,PF00636,PF00035 | | K01165 | AT1G01040.1 | DCL1 (DICER-LIKE 1); ATP-dependent helicase/ double-stranded RNA binding / protein binding / ribonuclease III |
| gma-miR162c | Glyma19g45060.2 | PF04851,PF00270,PF00271,PF03368,PF02170,PF00636,PF00035 | | K01165 | | |
| gma-miR319i | Glyma20g28640.1 | PF00190,PF07883 | | | AT3G22640.1 | PAP85; nutrient reservoir |
| gma-miR319d | Glyma20g28650.1 | PF00190,PF07883 | | | AT3G22640.1 | PAP85; nutrient reservoir |
| gma-miR319d | Glyma20g28660.1 | PF00190,PF07883 | | | AT3G22640.1 | PAP85; nutrient reservoir |
| gma-miR172j | Glyma02g36360.1 | PF04641 | | | AT5G58020.1 | unknown protein |
| gma-miR4387e | Glyma02g47560.1 | PF00504 | | K08913 | AT2G05100.1 | LHCB2.1; chlorophyll binding |
| gma-miR4387e | Glyma02g47560.2 | PF00504 | | K08913 | | |

| | | | | | | |
|---|---|---|---|---|---|---|
| gma-miR4382 | Glyma03g34080.1 | PF00664,PF00005 | | | AT2G36910.1 | ABCB1 (ATP BINDING CASSETTE SUBFAMILY B1); ATPase, coupled to transmembrane movement of substances / auxin efflux transmembrane transporter/ calmodulin binding |
| gma-miR167h | Glyma04g37030.1 | PF00227 | 3.4.25.1 | K02737 | AT3G26340.1 | 20S proteasome beta subunit E, putative |
| gma-miR156s | Glyma05g00200.1 | PF03110 | | | AT1G69170.1 | squamosa promoter-binding protein-like 6 (SPL6) |
| gma-miR1517 | Glyma05g10100.1 | PF00160,PF00515 ,PF07719 | | | AT2G15790.1 | SQN (SQUINT); peptidyl-prolyl cis-trans isomerase |
| gma-miR5035-5p | Glyma05g22060.1 | PF05922,PF00082 ,PF02225 | | | AT5G67360.1 | ARA12; serine-type endopeptidase |
| gma-miR164k | Glyma06g15100.2 | | | | | |
| gma-miR2108a | Glyma07g04950.4 | PF00847 | | | | |
| gma-miR5369 | Glyma08g28130.1 | | | | AT5G38840.1 | forkhead-associated domain-containing protein / FHA domain-containing protein |
| gma-miR482d-3p | Glyma08g41670.1 | PF00400 | | | AT5G08560.1 | transducin family protein / WD-40 repeat family protein |
| gma-miR396i-5p | Glyma09g07990.1 | PF08880,PF08879 | | | AT3G13960.1 | AtGRF5 (GROWTH-REGULATING FACTOR 5); transcription activator |
| gma-miR4998 | Glyma09g09050.1 | PF04043,PF01095 | 3.1.1.11 | K01051 | AT3G43270.1 | pectinesterase family protein |
| gma-miR396g | Glyma09g37520.2 | PF01992 | 3.6.3.14 | K02146 | | |
| gma-miR5036 | Glyma10g05750.1 | PF01569 | 3.6.1.43 | K07252 | AT5G03080.1 | phosphatidic acid phosphatase-related / PAP2-related |
| gma-miR394g | Glyma10g35100.1 | PF08246,PF00112 | | | AT4G39090.1 | RD19 (RESPONSIVE TO DEHYDRATION 19); cysteine-type endopeptidase/ cysteine-type peptidase |
| gma-miR390d | Glyma10g35510.1 | PF04389 | | | AT5G20660.1 | 24 kDa vacuolar protein, putative |
| gma-miR172j | Glyma10g38150.1 | PF00173 | | | AT5G52240.1 | MSBP1 (membrane steroid binding protein 1); steroid binding |
| gma-miR171k-3p | Glyma11g11760.1 | PF00137 | 3.6.3.14 | K02155 | AT4G38920.1 | ATVHA-C3 (VACUOLAR-TYPE H(+)-ATPASE C3); ATPase |
| gma-miR4382 | Glyma11g33470.1 | PF05450 | | | AT3G52640.1 | nicastrin-related |

| | | | | |
|---|---|---|---|---|
| gma-miR156f | Glyma12g12330.1 | PF05147 | AT2G20770.1 | GCL2 (GCR2-LIKE 2); catalytic |
| gma-miR2119 | Glyma13g09530.1 | PF08240,PF00107 | AT1G77120.1 | ADH1 (ALCOHOL DEHYDROGENASE 1); alcohol dehydrogenase |
| gma-miR5675 | Glyma13g17760.1 | PF04855 | AT3G17590.1 | BSH (BUSHY GROWTH); chromatin binding / protein binding |
| gma-miR166j-5p | Glyma13g18900.1 | | AT5G43970.1 | TOM22-V (TRANSLOCASE OF OUTER MEMBRANE 22-V); P-P-bond-hydrolysis-driven protein transmembrane transporter |
| gma-miR4380a | Glyma13g21960.1 | PF01754,PF01428 | AT3G52800.1 | zinc finger (AN1-like) family protein |
| gma-miR5669 | Glyma13g41370.1 | PF07244,PF01103 | AT3G46740.1 | TOC75-III (TRANSLOCON AT THE OUTER ENVELOPE MEMBRANE OF CHLOROPLASTS 75-III); P-P-bond-hydrolysis-driven protein transmembrane transporter |
| gma-miR4380a | Glyma14g11530.1 | PF00139,PF00069 ,PF07714 | AT5G10530.1 | lectin protein kinase, putative |
| gma-miR2119 | Glyma14g24860.1 | PF08240,PF00107 | AT1G77120.1 | ADH1 (ALCOHOL DEHYDROGENASE 1); alcohol dehydrogenase |
| gma-miR396c | Glyma15g19460.1 | PF08880,PF08879 | AT3G13960.1 | AtGRF5 (GROWTH-REGULATING FACTOR 5); transcription activator |
| gma-miR5672 | Glyma16g00260.1 | PF02485 | AT1G10280.1 | unknown protein |
| gma-miR1510b-5p | Glyma16g06100.1 | PF10539 | AT3G27090.1 | unknown protein |
| gma-miR1517 | Glyma16g32890.1 | PF03178 | AT5G51660.1 | CPSF160; nucleic acid binding |
| gma-miR319d | Glyma17g00700.1 | PF02798,PF00043 | AT2G02380.1 | ATGSTZ2; catalytic/ glutathione transferase |
| gma-miR1520q | Glyma17g05200.1 | PF00109,PF02801 | AT1G74960.1 | FAB1 (FATTY ACID BIOSYNTHESIS 1); 3-oxoacyl-[acyl-carrier-protein] synthase/ fatty-acid synthase |
| gma-miR395g | Glyma17g10050.1 | PF02704 | AT5G14920.1 | gibberellin-regulated family protein |
| gma-miR395g | Glyma17g10050.2 | PF02704 | | |

| | | | | | |
|---|---|---|---|---|---|
| gma-miR1523a | Glyma19g32920.1 | PF03760 | | AT5G06760.1 | late embryogenesis abundant group 1 domain-containing protein / LEA group 1 domain-containing protein |
| gma-miR319d | Glyma19g40550.1 | PF05691 | | AT4G01970.1 | AtSTS (Arabidopsis thaliana stachyose synthase); galactinol-raffinose galactosyltransferase/ hydrolase, hydrolyzing O-glycosyl compounds |
| gma-miR172g | Glyma19g42490.1 | | | AT1G03220.1 | extracellular dermal glycoprotein, putative / EDGP, putative |
