# Supplementary material for: Stress induced dynamic adjustment of conserved miR164:NAC module
Source: Plant Environ Interact. 2020 Aug 10;1(2):134–51. doi: 10.1002/pei3.10027 (PMC10168063; doi:10.1002/pei3.10027)
Supplement: Supplementary file 8 — TableS4 [file PEI3-1-134-s004.pdf]

Table S4: List of gene IDs for the sequences using in phylogenetic tree and gene duplication analysis

Phytozome ID	PTFD ID	Start Position	End Position	Duplication Type	At ortholog locus	At locus description	Function	References
Glyma.02g050100	Glyma02g05620.1	4538456	4541907	Segmental	AT4G36160.1	VND2	Vascular development	Tan et al 2018
Glyma.04g167200	Glyma04g33270.1	41938604	41940695	Segmental	AT5G61430.1	ANAC100	Leave senescence; Regulation of anthocyanin accumulation	Zhou et al 2015; Podzimska-Sroka et al. 2015
Glyma.04g175800	Glyma04g34530.1	43891286	43891596	Disperse	AT5G13180.1	VND2	Vascular development	Tan et al 2018
Glyma.05g002700	Glyma05g09110.8	192322	197211	Segmental	AT3G01600.1	ANAC044	Stress signals and leads to G2 arrest	Takahashi et al 2018
Glyma.05g025500	Glyma05g00930.1	2217936	2219956	Segmental	AT5G61430.1	ANAC100	Leave senescence; Regulation of anthocyanin accumulation	Zhou et al 2015; Podzimska-Sroka et al. 2015
Glyma.05g120500	Glyma05g24910.2	31355106	31357014	Segmental	AT5G64530.1	XND1	Xylem formation and aquaporin activity	Tang et al 2018
Glyma.05g234200	Glyma05g35090.1	41133563	41137142	Segmental	AT3G18400.1	ANAC058	Hypersensitive to NO for hypocotyl elongation of etiolated seedlings	Castillo et al.2018
Glyma.06g195500	Glyma06g21020.1	17486456	17488546	Segmental	AT5G61430.1	ANAC100	Leave senescence; Regulation of anthocyanin accumulation	Zhou et al 2015; Podzimska-Sroka et al. 2015
Glyma.06g236000	Glyma06g35660.1	38358744	38361292	Segmental	AT5G53950.1	CUC2	Shoot apical meristem and cotyledons formation	Aida et al. 1999
Glyma.07g048000	Glyma07g05360.1	4026951	4029750	Tandem	AT4G35580.1	NTL9	Mediator of osmotic stress responses that affect leaf senescence	Yoon et al. 2008
Glyma.07g048100	Glyma07g05370.1	4042093	4044551	Tandem	AT4G35580.1	NTL9	Mediator of osmotic stress responses that affect leaf senescence	Yoon et al. 2008
Glyma.07g192900	Glyma07g31220.1	36076875	36079545	Segmental	AT1G61110.1	ANAC025		

Glyma.08g041500	Glyma08g04610.1	3290821	3294321	Segmental	AT3G18400.1	ANAC058	Hypersensitive to NO for hypocotyl elongation of etiolated seedlings	
Glyma.08g075300	Glyma08g08010.1	5759118	5761050	Segmental	AT5G64530.1	XND1	Xylem formation and aquaporin activity	Tang et al 2018
Glyma.08g173400	Glyma08g18470.1	13802696	13807486	Segmental	AT1G56010.2	NAC21/22	Leave senescence, Strip rust resistence, lateral root development	Xu et al 2014;Podzimska-Sroka et al. 2015, Xie et al 2000
Glyma.08g360200	Glyma08g47520.1	47210855	47212576	Segmental	AT5G13180.1	VND2	Positive regulator of seed germination under salinity,Vascular development, developmental and stress response	Tan et al 2018; Melo et al 2018
Glyma.12g161700	Glyma12g26190.1	30307162	30309990	Segmental	AT5G53950.1	CUC2	Shoot apical meristem and cotyledons formation	Aida et al. 1999
Glyma.12g160100	Glyma12g18996.1	35155970	35163466	Segmental	AT5G13180.1	VND2	Vascular development	Tan et al 2018
Glyma.12g226500	Glyma12g35530.1	38629959	38632784	Segmental	AT5G53950.1	CUC2	Shoot apical meristem and cotyledons formation	Aida et al. 1999
Glyma.13g274300	Glyma13g34950.1	37580356	37583227	Segmental	AT5G53950.1	CUC2	Shoot apical meristem and cotyledons formation	Aida et al. 1999
Glyma.15g254000	Glyma15g40510.1	48295488	48300322	Segmental	AT1G56010.2	NAC21/22	Leave senescence, Strip rust resistence, lateral root development	Xu et al 2014;Podzimska-Sroka et al. 2015, Xie et al 2000
Glyma.17g101500	Glyma17g10970.1	7978357	7980301	Segmental	AT5G61430.1	ANAC100	Leave senescence; Regulation of anthocyanin accumulation	Zhou et al 2015; Podzimska-Sroka et al. 2015
Glyma.19g002900	Glyma19g00640.2	187517	192326	Segmental	AT3G01600.1	ANAC044	Stress signals and leads to G2 arrest.	Takahashi et al 2018
Glyma.19g021900	Glyma19g02580.1	2444946	2448347	Segmental	AT1G26870.1	FEZ	Orientation of cell division in root stem cells	Willemsen et al 2008
Glyma.19g024500	Glyma19g02850.1	2893626	2895846	Segmental	AT5G61430.1	ANAC100	Regulation of anthocyanin accumulation	Zhou et al 2015

Glyma.20g185800	Glyma20g32690.2	42438478	42443399	Segmental	AT1G25580.1	ANAC008		
LOC_Os01g48446.1	LOC_Os01g48446.1	27780948	27782266	Disperse	AT3G04070.2	ANAC47	Drought tolerance, Meiosis	Shim et al. 2018
LOC_Os01g70110.1	LOC_Os01g70110.1	40572819	40574020	Segmental	AT5G13180.1	VND2	Vascular development	Tan et al 2018
LOC_Os02g36880.1	LOC_Os02g36880.1	22258833	22260681	Segmental	AT5G61430.1	ANAC100	Leave senescence; Regulation of anthocyanin accumulation	Zhou et al 2015; Podzimska-Sroka et al. 2015
LOC_Os02g56600.1	LOC_Os02g56600.1	34672992	34676421	Disperse	AT4G28530.1	ANAC074	Leave senescence	Podzimska-Sroka et al. 2015
LOC_Os03g02800.1	LOC_Os03g02800.1	1089162	1093720	Disperse	AT1G65910.1	ANAC028		
LOC_Os03g12120.1	LOC_Os03g12120.1	6358095	6365595	Disperse	AT5G14490.1	ANAC085	Panicle, severel drought, Stress signals and leads to G2 arrest	Nuruzzaman et al. 2012; Takahashi et al 2018
LOC_Os03g42630.1	LOC_Os03g42630.1	23734580	23736564	Disperse	AT3G18400.1	ANAC058	Hypersensitive to NO for hypocotyl elongation of etiolated seedlings	Castillo et al.2018
LOC_Os03g56580.1	LOC_Os03g56580.1	32234311	32236602	Disperse	AT2G43000.1	ANAC042	Regulation of Camalexin Biosynthesis	Saga et al. 2012
LOC_Os03g61249.1	LOC_Os03g61249.1	34783894	34784844	Proximal	AT4G27410.2	RD26	Mediates crosstalk between drought and BR signalling.	Ye et al. 2017
LOC_Os03g61319.1	LOC_Os03g61319.1	34814137	34815087	Proximal	AT4G27410.2	RD26	Mediates crosstalk between drought and BR signalling.	Ye et al. 2017
LOC_Os03g61650.1	LOC_Os03g61650.1	34945841	34946935	Disperse	AT5G22380.1	ANAC090	Negatively regulates leaf senescence in Arabidopsis	Kim et al. 2018
LOC_Os04g38720.1	LOC_Os04g38720.1	22994552	22996740	Segmental	AT5G07680.2	ANAC080	Negatively regulate drought resistance in rice	Fang et al 2014
LOC_Os04g59470.1	LOC_Os04g59470.1	35358602	35359459	Segmental	AT5G66300.1	VND3	Vascular development	Tan et al 2018
LOC_Os05g34830.1	LOC_Os05g34830.1	20678001	20680343	Segmental	AT1G01720.1	ATAF1	Cold, drought , submergence	Nuruzzaman et al. 2010
LOC_Os05g37080.1	LOC_Os05g37080.1	21668094	21669730	Segmental	AT5G22380.1	ANAC090	Negatively regulates leaf senescence in Arabidopsis	Kim et al. 2018

LOC_Os05g43960.1	LOC_Os05g43960.1	25580132	25581765	Disperse	AT2G02450.2	ANAC035	Regulate the kinase activity of full-length phototropin2 from Arabidopsis	Oide et al. 2017
LOC_Os05g48850.1	LOC_Os05g48850.1	28003165	28005001	Segmental	AT4G28500.1	SND2	Secondary cell wall biosynthesis in Arabidopsis fibres and vessels	Hussey et al. 2011
LOC_Os06g01230.1	LOC_Os06g01230.1	153391	156618	Disperse	AT4G35580.1	NTL9	Mediator of osmotic stress responses that affect leaf senescence	Yoon et al. 2008
LOC_Os06g23650.1	LOC_Os06g23650.1	13804017	13805815	Disperse	AT5G53950.1	CUC2	Shoot apical meristem and cotyledons formation	Aida et al. 1999
LOC_Os06g46270.1	LOC_Os06g46270.1	28037569	28041909	Segmental	AT1G56010.2	ANAC21/22	negatively regulate drought resistance in rice	Fang et al 2014
LOC_Os07g27330.1	LOC_Os07g27330.1	15899431	15902146	Tandem	AT5G13180.1	VNI2	Vascular development	Tan et al 2018
LOC_Os07g48550.1	LOC_Os07g48550.1	29040433	29042297	Disperse	AT5G18270.2	ANAC087	Cold, drought , submergence	Nuruzzaman et al. 2010
LOC_Os08g02160.1	LOC_Os08g02160.1	732023	734595	Disperse	AT2G02450.2	ANAC035	Regulate the kinase activity of full-length phototropin2 from Arabidopsis	Oide et al. 2017
LOC_Os08g10080.1	LOC_Os08g10080.1	5846863	5850647	Segmental	AT1G56010.2	ANAC21/22	negatively regulate drought resistance in rice	Fang et al 2014
LOC_Os09g12380.1	LOC_Os09g12380.1	7077212	7078851	Disperse	AT3G55210.1	ANAC063		
LOC_Os11g04360.1	LOC_Os11g04360.1	1811713	1813623	Tandem				
LOC_Os12g07790.1	LOC_Os12g07790.1	3936377	3938092	Disperse	AT3G55210.1	ANAC063		
LOC_Os12g41680.1	LOC_Os12g41680.1	25800944	25806553	Disperse	AT1G56010.2	ANAC21/22	Cold, drought , submergence, negatively regulate drought resistance in rice	Fang et al 2014; Nuruzzaman et al. 2010
LOC_Os12g43530.1	LOC_Os12g43530.1	27007586	27009616	Disperse	AT2G43000.1	ANAC042	Regulation of Camalexin Biosynthesis	Saga et al. 2012
XP_016413159.1	XP_016413159.1	2376775	2379696					

XP_016436636.1	XP_016436636.1	85947	91119	AT1G56010.2	ANAC21/22	Leave senescence, Strip rust resistence, lateral root development	Xu et al 2014;Podzimska-Sroka et al. 2015, Xie et al 2000
XP_016437736.1	XP_016437736.1	55244	57792	AT5G61430.1	ANAC100	Leave senescence; Regulation of anthocyanin accumulation	Zhou et al 2015; Podzimska-Sroka et al. 2015
XP_016439814.1	XP_016439814.1	57771	60574	AT5G61430.1	ANAC100	Leave senescence; Regulation of anthocyanin accumulation	Zhou et al 2015; Podzimska-Sroka et al. 2015
XP_016439815.1	XP_016439815.1	57771	60574	AT5G61430.1	ANAC100	Leave senescence; Regulation of anthocyanin accumulation	Zhou et al 2015; Podzimska-Sroka et al. 2015
XP_016439816.1	XP_016439816.1	58114	60574	AT5G61430.1	ANAC100	Leave senescence; Regulation of anthocyanin accumulation	Zhou et al 2015; Podzimska-Sroka et al. 2015
XP_016440125.1	XP_016440125.1	59824	61660	AT5G61430.1	ANAC100	Leave senescence; Regulation of anthocyanin accumulation	Zhou et al 2015; Podzimska-Sroka et al. 2015
XP_016442160.1	XP_016442160.1	62112	64987	AT3G18400.1	ANAC58	Hypersensitive to NO for hypocotyl elongation of etiolated seedlings	Castillo et al.2018
XP_016442161.1	XP_016442161.1	62110	64987	AT3G18400.1	ANAC58	Hypersensitive to NO for hypocotyl elongation of etiolated seedlings	Castillo et al.2018
XP_016443696.1	XP_016443696.1	60247	61568				
XP_016451616.1	XP_016451616.1	55789	57537	AT5G61430.1	ANAC100	Leave senescence; Regulation of anthocyanin accumulation	Zhou et al 2015; Podzimska-Sroka et al. 2015

XP_016451623.1	XP_016451623.1	55789	57537	AT5G61430.1	ANAC100	Leave senescence; Regulation of anthocyanin accumulation	Zhou et al 2015; Podzimska-Sroka et al. 2015
XP_016457897.1	XP_016457897.1	11983	13550	AT5G61430.1	ANAC100	Leave senescence; Regulation of anthocyanin accumulation	Zhou et al 2015; Podzimska-Sroka et al. 2015
XP_016457898.1	XP_016457898.1	11983	13549	AT5G61430.1	ANAC100	Leave senescence; Regulation of anthocyanin accumulation	Zhou et al 2015; Podzimska-Sroka et al. 2015
XP_016460548.1	XP_016460548.1	35211	40741	AT1G56010.2	ANAC21/22	Leave senescence, Strip rust resistence, lateral root development	Xu et al 2014;Podzimska-Sroka et al. 2015, Xie et al 2000
XP_016460549.1	XP_016460549.1	35211	40741	AT1G56010.2	ANAC21/22	Leave senescence, Strip rust resistence, lateral root development	Xu et al 2014;Podzimska-Sroka et al. 2015, Xie et al 2000
XP_016466638.1	XP_016466638.1	17393	19431	AT5G61430.1	ANAC100	Leave senescence; Regulation of anthocyanin accumulation	Zhou et al 2015; Podzimska-Sroka et al. 2015
XP_016478779.1	XP_016478779.1	42638	45179	AT5G53950.1	CUC2	Shoot apical meristem and cotyledons formation	Aida et al. 1999
XP_016490965.1	XP_016490965.1	79654	81579	AT5G61430.1	ANAC100	Leave senescence; Regulation of anthocyanin accumulation	Zhou et al 2015; Podzimska-Sroka et al. 2015
XP_016504286.1	XP_016504286.1	45570	48264	AT3G18400.1	ANAC58	Hypersensitive to NO for hypocotyl elongation of etiolated seedlings	Castillo et al.2018
XP_016509208.1	XP_016509208.1	45662	48099	AT5G53950.1	CUC2	Shoot apical meristem and cotyledons formation	Aida et al. 1999
