# Supplementary material for: Stress induced dynamic adjustment of conserved miR164:NAC module
Source: Plant Environ Interact. 2020 Aug 10;1(2):134–51. doi: 10.1002/pei3.10027 (PMC10168063; doi:10.1002/pei3.10027)
Supplement: Supplementary file 9 — TableS5 [file PEI3-1-134-s009.pdf]

Table S5. List of predicted miR targeting NAC-TFs using psRNAtarget database

miRNA_Acc.	Target_Acc.	Expectation	Target_start	Target_end	Inhibition
gma-miR1514a-5p	Glyma07g05370.2	0.5	562	582	Cleavage
gma-miR1514a-5p	Glyma07g05370.1	0.5	715	735	Cleavage
gma-miR1514a-5p	Glyma07g05360.1	0.5	712	732	Cleavage
gma-miR1514a-5p	Glyma07g05360.2	0.5	712	732	Cleavage
gma-miR164a	Glyma05g00930.1	0.5	632	652	Cleavage
gma-miR164a	Glyma06g21020.1	0.5	659	679	Cleavage
gma-miR164a	Glyma05g00930.2	0.5	638	658	Cleavage
gma-miR164a	Glyma17g10970.1	0.5	638	658	Cleavage
gma-miR164b	Glyma05g00930.2	0.5	639	658	Cleavage
gma-miR164b	Glyma05g00930.1	0.5	633	652	Cleavage
gma-miR164b	Glyma17g10970.1	0.5	639	658	Cleavage
gma-miR164b	Glyma06g21020.1	0.5	660	679	Cleavage
gma-miR164c	Glyma05g00930.1	0.5	633	652	Cleavage
gma-miR164c	Glyma05g00930.2	0.5	639	658	Cleavage
gma-miR164c	Glyma17g10970.1	0.5	639	658	Cleavage
gma-miR164c	Glyma06g21020.1	0.5	660	679	Cleavage
gma-miR164d	Glyma05g00930.2	0.5	639	658	Cleavage
gma-miR164d	Glyma17g10970.1	0.5	639	658	Cleavage
gma-miR164d	Glyma05g00930.1	0.5	633	652	Cleavage
gma-miR164d	Glyma06g21020.1	0.5	660	679	Cleavage
gma-miR164e	Glyma06g21020.1	0.5	659	679	Cleavage
gma-miR164e	Glyma05g00930.2	0.5	638	658	Cleavage
gma-miR164e	Glyma17g10970.1	0.5	638	658	Cleavage
gma-miR164e	Glyma05g00930.1	0.5	632	652	Cleavage
gma-miR164f	Glyma05g00930.2	0.5	638	658	Cleavage
gma-miR164f	Glyma17g10970.1	0.5	638	658	Cleavage
gma-miR164f	Glyma05g00930.1	0.5	632	652	Cleavage
gma-miR164f	Glyma06g21020.1	0.5	659	679	Cleavage
gma-miR164g	Glyma05g00930.1	0.5	632	652	Cleavage
gma-miR164g	Glyma05g00930.2	0.5	638	658	Cleavage
gma-miR164g	Glyma17g10970.1	0.5	638	658	Cleavage
gma-miR164g	Glyma06g21020.1	0.5	659	679	Cleavage
gma-miR164h	Glyma06g21020.1	0.5	659	679	Cleavage
gma-miR164h	Glyma05g00930.1	0.5	632	652	Cleavage
gma-miR164h	Glyma05g00930.2	0.5	638	658	Cleavage
gma-miR164h	Glyma17g10970.1	0.5	638	658	Cleavage
gma-miR164i	Glyma05g00930.2	0.5	638	658	Cleavage
gma-miR164i	Glyma17g10970.1	0.5	638	658	Cleavage
gma-miR164i	Glyma05g00930.1	0.5	632	652	Cleavage
gma-miR164i	Glyma06g21020.1	0.5	659	679	Cleavage
gma-miR164j	Glyma05g00930.2	0.5	638	658	Cleavage
gma-miR164j	Glyma17g10970.1	0.5	638	658	Cleavage
gma-miR164j	Glyma06g21020.1	0.5	659	679	Cleavage
gma-miR164j	Glyma05g00930.1	0.5	632	652	Cleavage
gma-miR164k	Glyma06g21020.1	0.5	659	679	Cleavage
gma-miR164k	Glyma05g00930.2	0.5	638	658	Cleavage
gma-miR164k	Glyma17g10970.1	0.5	638	658	Cleavage
gma-miR164k	Glyma05g00930.1	0.5	632	652	Cleavage
gma-miR164a	Glyma06g35660.1	1	800	820	Cleavage

gma-miR164a	Glyma12g26190.1	1	767	787	Cleavage
gma-miR164a	Glyma12g35530.1	1	701	721	Cleavage
gma-miR164a	Glyma13g34950.1	1	716	736	Cleavage
gma-miR164a	Glyma08g18470.1	1	602	622	Cleavage
gma-miR164a	Glyma15g40510.1	1	602	622	Cleavage
gma-miR164b	Glyma12g26190.1	1	768	787	Cleavage
gma-miR164b	Glyma13g34950.1	1	717	736	Cleavage
gma-miR164b	Glyma12g35530.1	1	702	721	Cleavage
gma-miR164b	Glyma06g35660.1	1	801	820	Cleavage
gma-miR164b	Glyma08g18470.1	1	603	622	Cleavage
gma-miR164b	Glyma15g40510.1	1	603	622	Cleavage
gma-miR164c	Glyma12g26190.1	1	768	787	Cleavage
gma-miR164c	Glyma13g34950.1	1	717	736	Cleavage
gma-miR164c	Glyma12g35530.1	1	702	721	Cleavage
gma-miR164c	Glyma06g35660.1	1	801	820	Cleavage
gma-miR164c	Glyma15g40510.1	1	603	622	Cleavage
gma-miR164c	Glyma08g18470.1	1	603	622	Cleavage
gma-miR164d	Glyma12g26190.1	1	768	787	Cleavage
gma-miR164d	Glyma13g34950.1	1	717	736	Cleavage
gma-miR164d	Glyma12g35530.1	1	702	721	Cleavage
gma-miR164d	Glyma06g35660.1	1	801	820	Cleavage
gma-miR164d	Glyma15g40510.1	1	603	622	Cleavage
gma-miR164d	Glyma08g18470.1	1	603	622	Cleavage
gma-miR164e	Glyma06g35660.1	1	800	820	Cleavage
gma-miR164e	Glyma12g35530.1	1	701	721	Cleavage
gma-miR164e	Glyma12g26190.1	1	767	787	Cleavage
gma-miR164e	Glyma13g34950.1	1	716	736	Cleavage
gma-miR164e	Glyma15g40510.1	1	602	622	Cleavage
gma-miR164e	Glyma08g18470.1	1	602	622	Cleavage
gma-miR164f	Glyma13g34950.1	1	716	736	Cleavage
gma-miR164f	Glyma06g35660.1	1	800	820	Cleavage
gma-miR164f	Glyma12g35530.1	1	701	721	Cleavage
gma-miR164f	Glyma12g26190.1	1	767	787	Cleavage
gma-miR164f	Glyma15g40510.1	1	602	622	Cleavage
gma-miR164f	Glyma08g18470.1	1	602	622	Cleavage
gma-miR164g	Glyma12g35530.1	1	701	721	Cleavage
gma-miR164g	Glyma13g34950.1	1	716	736	Cleavage
gma-miR164g	Glyma06g35660.1	1	800	820	Cleavage
gma-miR164g	Glyma12g26190.1	1	767	787	Cleavage
gma-miR164g	Glyma15g40510.1	1	602	622	Cleavage
gma-miR164g	Glyma08g18470.1	1	602	622	Cleavage
gma-miR164h	Glyma12g26190.1	1	767	787	Cleavage
gma-miR164h	Glyma13g34950.1	1	716	736	Cleavage
gma-miR164h	Glyma06g35660.1	1	800	820	Cleavage
gma-miR164h	Glyma12g35530.1	1	701	721	Cleavage
gma-miR164h	Glyma15g40510.1	1	602	622	Cleavage
gma-miR164h	Glyma08g18470.1	1	602	622	Cleavage
gma-miR164i	Glyma12g26190.1	1	767	787	Cleavage
gma-miR164i	Glyma12g35530.1	1	701	721	Cleavage
gma-miR164i	Glyma13g34950.1	1	716	736	Cleavage
gma-miR164i	Glyma06g35660.1	1	800	820	Cleavage

gma-miR164i	Glyma08g18470.1	1	602	622	Cleavage
gma-miR164i	Glyma15g40510.1	1	602	622	Cleavage
gma-miR164j	Glyma12g35530.1	1	701	721	Cleavage
gma-miR164j	Glyma13g34950.1	1	716	736	Cleavage
gma-miR164j	Glyma06g35660.1	1	800	820	Cleavage
gma-miR164j	Glyma12g26190.1	1	767	787	Cleavage
gma-miR164j	Glyma15g40510.1	1	602	622	Cleavage
gma-miR164j	Glyma08g18470.1	1	602	622	Cleavage
gma-miR164k	Glyma12g26190.1	1	767	787	Cleavage
gma-miR164k	Glyma13g34950.1	1	716	736	Cleavage
gma-miR164k	Glyma12g35530.1	1	701	721	Cleavage
gma-miR164k	Glyma06g35660.1	1	800	820	Cleavage
gma-miR164k	Glyma08g18470.1	1	602	622	Cleavage
gma-miR164k	Glyma15g40510.1	1	602	622	Cleavage
gma-miR1514b-5p	Glyma07g05370.2	1.5	562	582	Cleavage
gma-miR1514b-5p	Glyma07g05370.1	1.5	715	735	Cleavage
gma-miR1514b-5p	Glyma07g05360.1	1.5	712	732	Cleavage
gma-miR1514b-5p	Glyma07g05360.2	1.5	712	732	Cleavage
gma-miR164a	Glyma04g33270.1	1.5	656	676	Cleavage
gma-miR164b	Glyma04g33270.1	1.5	657	676	Cleavage
gma-miR164c	Glyma04g33270.1	1.5	657	676	Cleavage
gma-miR164d	Glyma04g33270.1	1.5	657	676	Cleavage
gma-miR164e	Glyma04g33270.1	1.5	656	676	Cleavage
gma-miR164f	Glyma04g33270.1	1.5	656	676	Cleavage
gma-miR164g	Glyma04g33270.1	1.5	656	676	Cleavage
gma-miR164h	Glyma04g33270.1	1.5	656	676	Cleavage
gma-miR164i	Glyma04g33270.1	1.5	656	676	Cleavage
gma-miR164j	Glyma04g33270.1	1.5	656	676	Cleavage
gma-miR164k	Glyma04g33270.1	1.5	656	676	Cleavage
gma-miR9756	Glyma08g08010.1	2	511	532	Cleavage
gma-miR9756	Glyma05g24910.2	2	508	529	Cleavage
gma-miR9756	Glyma05g24910.1	2	508	529	Cleavage
gma-miR5775	Glyma02g05620.1	2.5	806	826	Cleavage
gma-miR1514a-5p	Glyma16g01911.1	3	694	714	Cleavage
gma-miR1515a	Glyma07g31220.1	3	734	755	Cleavage
gma-miR1515b	Glyma07g31220.1	3	734	755	Cleavage
gma-miR1518	Glyma05g35090.1	3	801	821	Cleavage
gma-miR1518	Glyma08g04610.1	3	768	788	Cleavage
gma-miR167a	Glyma19g02850.1	3	730	750	Cleavage
gma-miR167b	Glyma19g02850.1	3	730	750	Cleavage
gma-miR167c	Glyma19g02850.1	3	730	750	Cleavage
gma-miR167d	Glyma19g02850.1	3	730	750	Cleavage
gma-miR167e	Glyma19g02850.1	3	730	750	Cleavage
gma-miR167f	Glyma19g02850.1	3	730	750	Cleavage
gma-miR167g	Glyma19g02850.1	3	729	750	Cleavage
gma-miR167j	Glyma19g02850.1	3	730	750	Cleavage
gma-miR319a	Glyma19g02580.1	3	978	997	Cleavage
gma-miR319b	Glyma19g02580.1	3	978	997	Cleavage
gma-miR319e	Glyma19g02580.1	3	978	997	Cleavage
gma-miR319g	Glyma19g02580.1	3	976	997	Cleavage
gma-miR319h	Glyma19g02580.1	3	977	997	Cleavage

gma-miR319j	Glyma19g02580.1	3	977	997	Cleavage
gma-miR319k	Glyma19g02580.1	3	977	997	Cleavage
gma-miR319l	Glyma19g02580.1	3	976	997	Cleavage
gma-miR319m	Glyma19g02580.1	3	977	997	Cleavage
gma-miR390a-5p	Glyma04g34530.1	3	52	72	Cleavage
gma-miR390a-5p	Glyma08g47520.1	3	127	147	Cleavage
gma-miR390a-5p	Glyma12g18996.1	3	55	75	Cleavage
gma-miR390a-5p	Glyma10g20830.2	3	55	75	Cleavage
gma-miR390f	Glyma08g47520.1	3	127	147	Cleavage
gma-miR390f	Glyma04g34530.1	3	52	72	Cleavage
gma-miR390f	Glyma12g18996.1	3	55	75	Cleavage
gma-miR390f	Glyma10g20830.2	3	55	75	Cleavage
gma-miR390g	Glyma04g34530.1	3	52	72	Cleavage
gma-miR390g	Glyma08g47520.1	3	127	147	Cleavage
gma-miR390g	Glyma12g18996.1	3	55	75	Cleavage
gma-miR390g	Glyma10g20830.2	3	55	75	Cleavage
gma-miR396g	Glyma19g00640.2	3	684	704	Cleavage
gma-miR396g	Glyma05g09110.7	3	240	260	Cleavage
gma-miR396g	Glyma05g09110.3	3	492	512	Cleavage
gma-miR396g	Glyma05g09110.4	3	486	506	Cleavage
gma-miR396g	Glyma05g09110.6	3	492	512	Cleavage
gma-miR396g	Glyma05g09110.8	3	240	260	Cleavage
gma-miR396g	Glyma05g09110.5	3	501	521	Cleavage
gma-miR396g	Glyma05g09110.2	3	501	521	Cleavage
gma-miR5780d	Glyma20g32690.2	3	943	964	Cleavage
gma-miR5780d	Glyma20g32690.3	3	895	916	Cleavage
osa-miR164a	LOC_Os06g23650.1	1	794	814	Cleavage
osa-miR164a	LOC_Os12g41680.1	1	692	712	Cleavage
osa-miR164a	LOC_Os06g46270.1	1	668	688	Cleavage
osa-miR164b	LOC_Os12g41680.1	1	692	712	Cleavage
osa-miR164b	LOC_Os06g46270.1	1	668	688	Cleavage
osa-miR164b	LOC_Os06g23650.1	1	794	814	Cleavage
osa-miR164d	LOC_Os06g46270.1	1	668	688	Cleavage
osa-miR164d	LOC_Os12g41680.1	1	692	712	Cleavage
osa-miR164d	LOC_Os06g23650.1	1	794	814	Cleavage
osa-miR164e	LOC_Os06g46270.1	1	668	688	Cleavage
osa-miR164e	LOC Os06g23650.1	1	794	814	Cleavage
osa-miR164e	LOC_Os12g41680.1	1	692	712	Cleavage
osa-miR164f	LOC_Os06g46270.1	1	668	688	Cleavage
osa-miR164f	LOC_Os06g23650.1	1	794	814	Cleavage
osa-miR164f	LOC_Os12g41680.1	1	692	712	Cleavage
osa-miR2102-5p	LOC_Os04g59470.1	1	602	621	Cleavage
osa-miR164a	LOC_Os02g36880.1	1.5	776	796	Cleavage
osa-miR164a	LOC_Os02g36880.2	1.5	776	796	Cleavage
osa-miR164a	LOC Os02g36880.3	1.5	776	796	Cleavage
osa-miR164a	LOC_Os02g36880.4	1.5	773	793	Cleavage
osa-miR164a	LOC_Os04g38720.1	1.5	686	706	Cleavage
osa-miR164b	LOC_Os02g36880.1	1.5	776	796	Cleavage
osa-miR164b	LOC_Os02g36880.2	1.5	776	796	Cleavage
osa-miR164b	LOC_Os02g36880.3	1.5	776	796	Cleavage
osa-miR164b	LOC_Os04g38720.1	1.5	686	706	Cleavage

osa-miR164b	LOC_Os02g36880.4	1.5	773	793	Cleavage
osa-miR164c	LOC_Os06g23650.1	1.5	794	814	Cleavage
osa-miR164c	LOC_Os12g41680.1	1.5	692	712	Cleavage
osa-miR164c	LOC_Os06g46270.1	1.5	668	688	Cleavage
osa-miR164d	LOC_Os02g36880.4	1.5	773	793	Cleavage
osa-miR164d	LOC_Os04g38720.1	1.5	686	706	Cleavage
osa-miR164d	LOC_Os02g36880.1	1.5	776	796	Cleavage
osa-miR164d	LOC_Os02g36880.3	1.5	776	796	Cleavage
osa-miR164d	LOC_Os02g36880.2	1.5	776	796	Cleavage
osa-miR164e	LOC_Os04g38720.1	1.5	686	706	Cleavage
osa-miR164e	LOC_Os02g36880.1	1.5	776	796	Cleavage
osa-miR164e	LOC_Os02g36880.3	1.5	776	796	Cleavage
osa-miR164e	LOC_Os02g36880.2	1.5	776	796	Cleavage
osa-miR164e	LOC_Os02g36880.4	1.5	773	793	Cleavage
osa-miR164f	LOC_Os04g38720.1	1.5	686	706	Cleavage
osa-miR164f	LOC_Os02g36880.4	1.5	773	793	Cleavage
osa-miR164f	LOC_Os02g36880.1	1.5	776	796	Cleavage
osa-miR164f	LOC_Os02g36880.3	1.5	776	796	Cleavage
osa-miR164f	LOC_Os02g36880.2	1.5	776	796	Cleavage
osa-miR5075	LOC_Os05g34830.1	1.5	18	38	Cleavage
osa-miR820a	LOC_Os01g48446.1	1.5	120	140	Cleavage
osa-miR820b	LOC_Os01g48446.1	1.5	120	140	Cleavage
osa-miR820c	LOC_Os01g48446.1	1.5	120	140	Cleavage
osa-miR164a	LOC_Os08g10080.1	2	686	706	Cleavage
osa-miR164b	LOC_Os08g10080.1	2	686	706	Cleavage
osa-miR164c	LOC_Os02g36880.4	2	773	793	Cleavage
osa-miR164c	LOC_Os02g36880.1	2	776	796	Cleavage
osa-miR164c	LOC_Os02g36880.3	2	776	796	Cleavage
osa-miR164c	LOC_Os02g36880.2	2	776	796	Cleavage
osa-miR164c	LOC_Os04g38720.1	2	686	706	Cleavage
osa-miR164d	LOC_Os08g10080.1	2	686	706	Cleavage
osa-miR164e	LOC_Os08g10080.1	2	686	706	Cleavage
osa-miR164f	LOC_Os08g10080.1	2	686	706	Cleavage
osa-miR2097-3p	LOC_Os11g04360.1	2	1228	1249	Cleavage
osa-miR5075	LOC_Os12g43530.1	2	297	317	Cleavage
osa-miR164c	LOC_Os08g10080.1	2.5	686	706	Cleavage
osa-miR5075	LOC_Os03g12120.1	2.5	150	170	Cleavage
osa-miR5075	LOC_Os08g02160.1	2.5	43	63	Cleavage
osa-miR5809	LOC_Os03g56580.1	2.5	930	949	Cleavage
osa-miR166i-3p	LOC_Os05g48850.1	3	200	220	Cleavage
osa-miR1846a-5p	LOC_Os07g48550.1	3	337	357	Cleavage
osa-miR1846b-5p	LOC_Os07g48550.1	3	337	357	Cleavage
osa-miR1846c-5p	LOC_Os07g48550.1	3	337	357	Cleavage
osa-miR1848	LOC_Os05g37080.1	3	355	375	Cleavage
osa-miR2925	LOC_Os01g48446.1	3	489	507	Cleavage
osa-miR2925	LOC_Os09g12380.1	3	109	127	Cleavage
osa-miR2926	LOC_Os03g61650.1	3	82	101	Translation
osa-miR5075	LOC_Os03g61319.1	3	543	563	Cleavage
osa-miR5075	LOC_Os03g61249.1	3	543	563	Cleavage
osa-miR5075	LOC_Os01g70110.1	3	613	633	Cleavage
osa-miR5075	LOC_Os05g43960.1	3	321	341	Translation

osa-miR5075	LOC_Os03g42630.1	3	619	639	Cleavage
osa-miR5075	LOC_Os07g27330.1	3	319	339	Cleavage
osa-miR5075	LOC_Os12g07790.1	3	403	423	Cleavage
osa-miR5150-3p	LOC_Os03g02800.1	3	596	619	Translation
osa-miR5493	LOC_Os02g56600.1	3	278	298	Translation
osa-miR5827	LOC_Os06g01230.2	3	919	939	Cleavage
osa-miR5827	LOC_Os06g01230.1	3	919	939	Cleavage
nta-miR164a	XP_016509208.1	0.5	686	706	Cleavage
nta-miR164a	XP_016478779.1	0.5	686	706	Cleavage
nta-miR164b	XP_016509208.1	0.5	686	706	Cleavage
nta-miR164b	XP_016478779.1	0.5	686	706	Cleavage
nta-miR164a	XP_016460549.1	1	659	679	Cleavage
nta-miR164a	XP_016460548.1	1	662	682	Cleavage
nta-miR164a	XP_016436636.1	1	686	706	Cleavage
nta-miR164b	XP_016460549.1	1	659	679	Cleavage
nta-miR164b	XP_016436636.1	1	686	706	Cleavage
nta-miR164b	XP_016460548.1	1	662	682	Cleavage
nta-miR164c	XP_016460548.1	1	662	682	Cleavage
nta-miR164c	XP_016436636.1	1	686	706	Cleavage
nta-miR164c	XP_016460549.1	1	659	679	Cleavage
nta-miR164a	XP_016451616.1	1.5	614	634	Cleavage
nta-miR164a	XP_016439815.1	1.5	647	667	Cleavage
nta-miR164a	XP_016439814.1	1.5	647	667	Cleavage
nta-miR164a	XP_016457898.1	1.5	611	631	Cleavage
nta-miR164a	XP_016451623.1	1.5	611	631	Cleavage
nta-miR164a	XP_016457897.1	1.5	614	634	Cleavage
nta-miR164a	XP_016439816.1	1.5	500	520	Cleavage
nta-miR164a	XP_016466638.1	1.5	620	640	Cleavage
nta-miR164a	XP_016463159.1	1.5	611	631	Cleavage
nta-miR164b	XP_016451616.1	1.5	614	634	Cleavage
nta-miR164b	XP_016439815.1	1.5	647	667	Cleavage
nta-miR164b	XP_016439814.1	1.5	647	667	Cleavage
nta-miR164b	XP_016457898.1	1.5	611	631	Cleavage
nta-miR164b	XP_016439816.1	1.5	500	520	Cleavage
nta-miR164b	XP_016466638.1	1.5	620	640	Cleavage
nta-miR164b	XP_016463159.1	1.5	611	631	Cleavage
nta-miR164b	XP_016451623.1	1.5	611	631	Cleavage
nta-miR164b	XP_016457897.1	1.5	614	634	Cleavage
nta-miR164c	XP_016509208.1	1.5	686	706	Cleavage
nta-miR164c	XP_016478779.1	1.5	686	706	Cleavage
nta-miR164c	XP_016437736.1	1.5	659	679	Cleavage
nta-miR164c	XP_016490965.1	1.5	659	679	Cleavage
nta-miR164a	XP_016490965.1	2	659	679	Cleavage
nta-miR164a	XP_016437736.1	2	659	679	Cleavage
nta-miR164b	XP_016490965.1	2	659	679	Cleavage
nta-miR164b	XP_016437736.1	2	659	679	Cleavage
nta-miR164c	XP_016443693.1	2	656	676	Cleavage
nta-miR164c	XP_016440125.1	2	656	676	Cleavage
nta-miR164a	XP_016440125.1	2.5	656	676	Cleavage
nta-miR164a	XP_016443693.1	2.5	656	676	Cleavage
nta-miR164b	XP_016440125.1	2.5	656	676	Cleavage

nta-miR164b	XP_016443693.1	2.5	656	676	Cleavage
nta-miR164c	XP_016463159.1	2.5	611	631	Cleavage
nta-miR164c	XP_016439815.1	2.5	647	667	Cleavage
nta-miR164c	XP_016439814.1	2.5	647	667	Cleavage
nta-miR164c	XP_016457898.1	2.5	611	631	Cleavage
nta-miR164c	XP_016457897.1	2.5	614	634	Cleavage
nta-miR164c	XP_016451623.1	2.5	611	631	Cleavage
nta-miR164c	XP_016439816.1	2.5	500	520	Cleavage
nta-miR164c	XP_016466638.1	2.5	620	640	Cleavage
nta-miR164c	XP_016451616.1	2.5	614	634	Cleavage
nta-miR396a	XP_016451616.1	3	106	126	Cleavage
nta-miR396a	XP_016451623.1	3	106	126	Cleavage
nta-miR396a	XP_016466638.1	3	115	135	Cleavage
nta-miR396a	XP_016463159.1	3	106	126	Cleavage
nta-miR396a	XP_016457897.1	3	106	126	Cleavage
nta-miR396a	XP_016457898.1	3	106	126	Cleavage
nta-miR396b	XP_016457897.1	3	106	126	Cleavage
nta-miR396b	XP_016451623.1	3	106	126	Cleavage
nta-miR396b	XP_016466638.1	3	115	135	Cleavage
nta-miR396b	XP_016457898.1	3	106	126	Cleavage
nta-miR396b	XP_016463159.1	3	106	126	Cleavage
nta-miR396b	XP_016451616.1	3	106	126	Cleavage
nta-miR396c	XP_016457897.1	3	106	126	Cleavage
nta-miR396c	XP_016451623.1	3	106	126	Cleavage
nta-miR396c	XP_016451616.1	3	106	126	Cleavage
nta-miR396c	XP_016466638.1	3	115	135	Cleavage
nta-miR396c	XP_016457898.1	3	106	126	Cleavage
nta-miR396c	XP_016463159.1	3	106	126	Cleavage
nta-miR6158a	XP_016442161.1	3	199	220	Cleavage
nta-miR6158a	XP_016504286.1	3	220	241	Cleavage
nta-miR6158a	XP_016442160.1	3	220	241	Cleavage
nta-miR6158b	XP_016442161.1	3	199	220	Cleavage
nta-miR6158b	XP_016504286.1	3	220	241	Cleavage
nta-miR6158b	XP_016442160.1	3	220	241	Cleavage
nta-miR6158c	XP_016504286.1	3	220	241	Cleavage
nta-miR6158c	XP_016442161.1	3	199	220	Cleavage
nta-miR6158c	XP_016442160.1	3	220	241	Cleavage
